# Catalytic asymmetric direct aldol reaction of α-alkyl azlactones and aliphatic aldehydes†
†Electronic supplementary information (ESI) available: Experimental procedures and characterization for new compounds are provided. See DOI: 10.1039/c5sc02116b


**DOI:** 10.1039/c5sc02116b

**Published:** 2015-08-04

**Authors:** Yang Zheng, Li Deng

**Affiliations:** a Department of Chemistry , Brandeis University , Waltham , Massachusetts 02454-9110 , USA . Email: deng@brandeis.edu

## Abstract

An unprecedented highly diastereoselective and enantioselective aldol reaction of α-alkyl azlactones and aliphatic aldehydes was achieved with cinchona alkaloid catalysts. To our knowledge, this reaction provides the first useful catalytic asymmetric access toward β-hydroxy-α-amino acids bearing alkyl substituents, which are structural motifs embedded in many natural products.

## Abstract 2

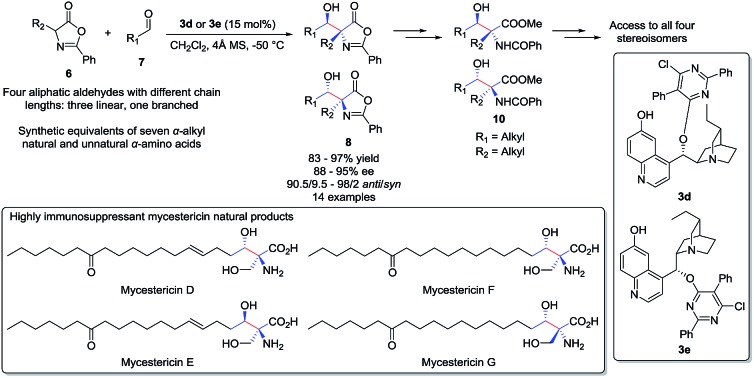
An unprecedented diastereoselective and enantioselective catalytic direct aldol reaction generating β-hydroxy-α-amino acid derivatives with alkyl-substituted adjacent quaternary-tertiary stereocenters.

## 

Optically active β-hydroxy-α-amino acids are an important class of amino acids as they are structural motifs in many biologically active natural products such as vancomycin,^1^ katanosins,^2^ cyclosporin,^3^ myriocin,4a,b mycestericins,4c,d sphingosine and threonine (Fig. 1). Furthermore, these amino acids are also useful chiral building blocks in organic synthesis as precursors to β-lactams,^5^ β-halo-α-amino acids,^6^ and aziridines.^7^ A variety of catalytic asymmetric approaches for the synthesis of β-hydroxy-α-amino acids has been reported.^8^–^14^ In a pioneering study,8*a* Ito, Hayashi and coworkers reported a gold-catalyzed highly diastereoselective and enantioselective aldol reaction for the generation of β-hydroxy-α-amino acids containing tertiary α-carbons. Since then other groups have also reported asymmetric direct aldol reactions with chiral transition-metal catalysts,8c–h organocatalysts^9^ and aldolases^10^ for the synthesis of β-hydroxy-α-amino acids and their derivatives. In addition, Sharpless asymmetric aminohydroxylation,^11^ transition-metal-catalyzed asymmetric hydrogenation,^12^ palladium-catalyzed allylic alkylation^13^ and chiral phosphoric acid-catalyzed addition to oxocarbenium ion^14^ have been utilized to achieve the same goal.

**Fig. 1:**
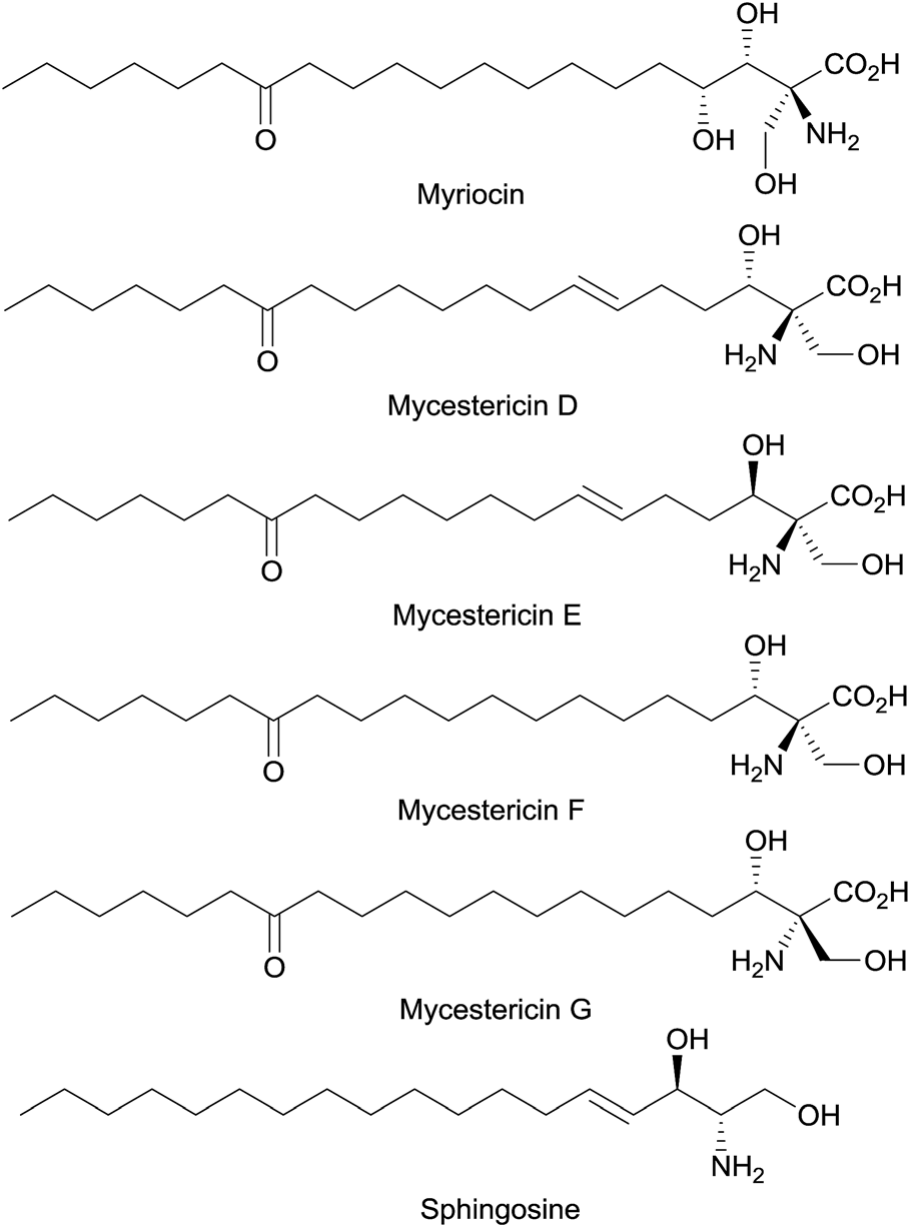
Mycestericins: potent immunosuppressant natural products

We became interested in the development of catalytic asymmetric synthesis of β-hydroxy-α-amino acids because biologically interesting natural products such as mycestericins contain a chiral β-hydroxy-α-amino acid motif that could not be constructed from existing catalytic asymmetric aldol reactions. In particular this motif presents both a tertiary β-stereocenter and a quaternary α-stereocenter with alkyl substituents. In principle, an efficient catalytic asymmetric aldol reaction of α-alkyl enolates or the equivalents with aliphatic aldehydes could provide a direct access to this structural motif.^15^ However, to our knowledge, such an asymmetric transformation was not available. Herein, we report the first efficient catalytic asymmetric direct aldol reaction of α-alkyl azlactones **6** and aliphatic aldehydes **7** (Scheme 1), which provides, to our knowledge, the first useful asymmetric catalytic access toward β-hydroxy-α-amino acids bearing alkyl substituents at both the tertiary β-stereocenter and the quaternary α-stereocenter. The high *anti*-diastereoselectivity in combination with a broad substrate scope allows the reaction to complement existing methods to form a general strategy for the asymmetric synthesis of β-hydroxy-α-amino acids.

**Scheme 1:**
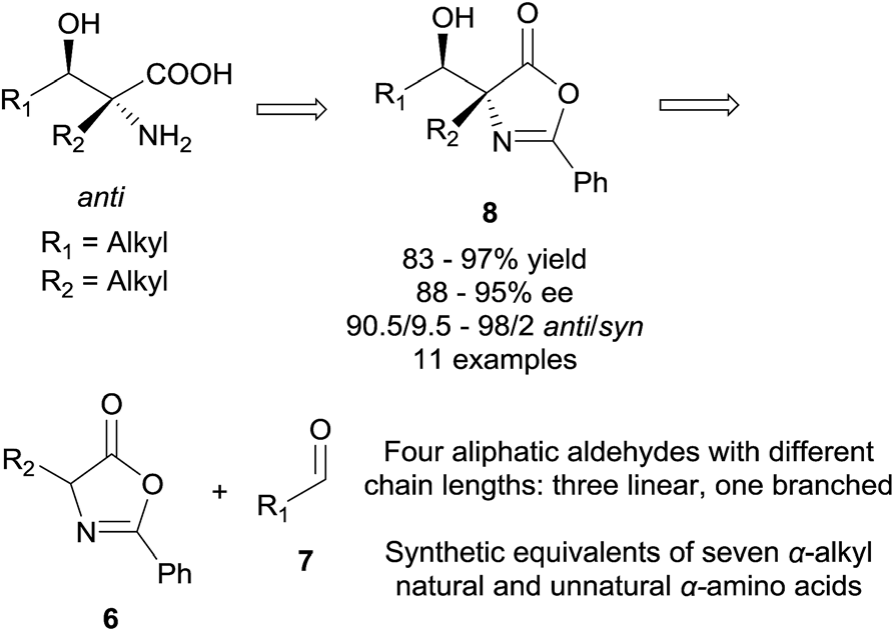
Reaction design

We initiated our study by reacting azlactone **6a** and aldehyde **7a** in the presence of a stoichiometric amount of triethylamine. After considerable experiments, we found that a reaction could be reasonably fast and clean at –20 °C in chloroform. We next investigated the possibility of promoting an asymmetric variant of this reaction with cinchona alkaloid-derived catalysts (Fig. 2). Upon first screening of a series cinchona alkaloid derivatives, (entries 1–9, Table 1), we identified the 6′-OH cinchona alkaloid **3d** as the most promising catalyst in terms of affording high diastereoselectivity and enantioselectivity (entry 6, Table 1). Catalyst **3e**, the pseudo-enantiomer of **3d**, gave comparable results with an expected reverse sense of asymmetric induction (entry 7, Table 1). Following these results, we carried out the **3d**-promoted aldol reaction in a variety of solvents with azlactone **6a** at a significantly decreased concentration of 0.5 M (entry 10–15). We found that the reaction at the reduced concentration proceeded in higher diastereo- and enantioselectivity (entry 10 *vs.* 6). Moreover, the reaction in dichloromethane occurred in a slightly higher diastereoselectivity than and the same enantioselectivity as the reaction in chloroform (entries 11–10, Table 1). Both the diastereoselectivity and enantioselectivity afforded by catalyst **3d** could be improved significantly when the reaction was performed at significantly reduced temperature and concentration (entry 16 *vs.* 11), although a higher catalyst loading and an extended reaction time were required for the reaction to proceed to completion. Importantly, under these conditions, a highly diastereoselective and enantioselective aldol reaction was established to generate the desired aldol product **8aa** in 92% isolated yield, 94% ee and 97.5/2.5 *anti*/*syn* ratio. It should be noted that no product resulted from the self-aldol reaction by aldehyde **7a** was detected by NMR analysis.

**Fig. 2:**
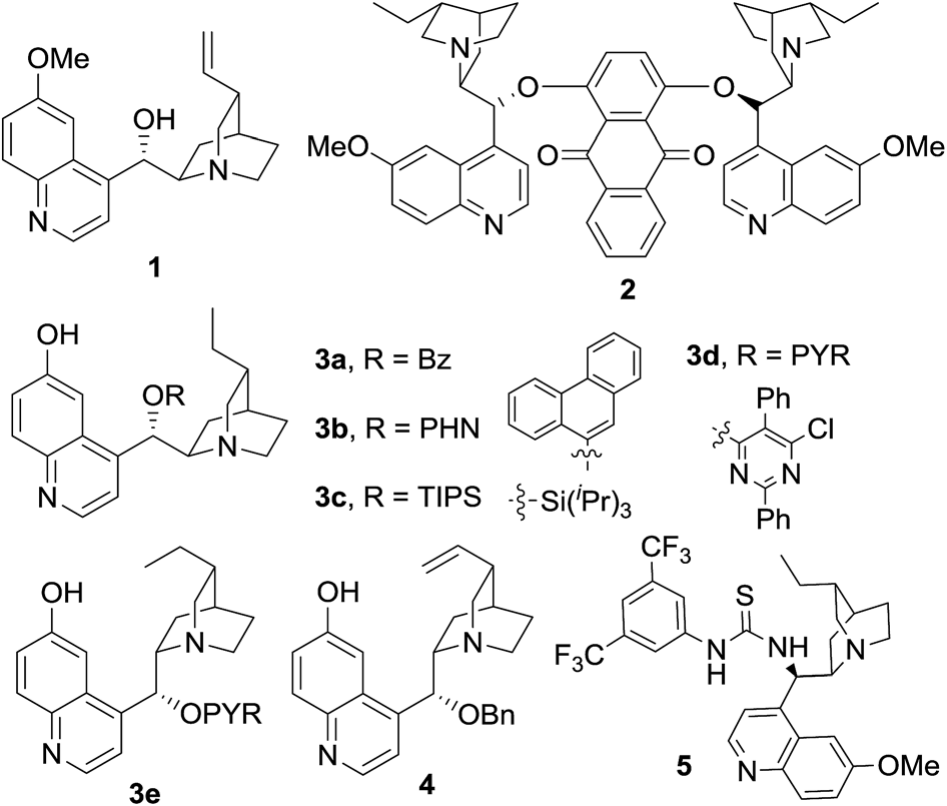
Cinchona alkaloid catalysts

**Table 1:** Catalytic asymmetric aldol reaction of azlactone **6a** and aldehyde **7a**^*a*^

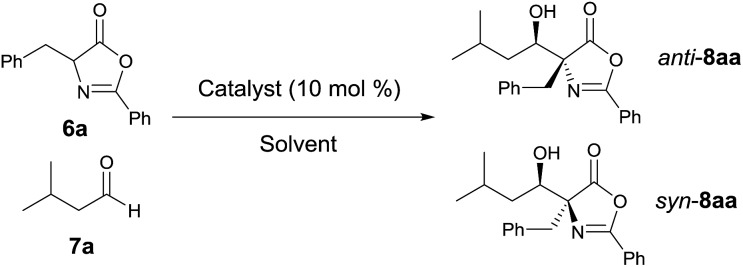							
Entry	Catalyst	Solvent	Temp (°C)	Time	Conv.^*c*^ (%)	ee^*c*^ ^*d*^ (%)	*anti*/*syn*^*c*^
1	**1**	CHCl_3_ (2 M)	–20	15 h	>95	29/22	43.5/56.5
2	**2**	CHCl_3_ (2 M)	–20	15 h	93	43/12	40.5/59.5
3	**3a**	CHCl_3_ (2 M)	–20	15 h	>95	57/25	81/19
4	**3b**	CHCl_3_ (2 M)	–20	15 h	>95	51/19	73/27
5	**3c**	CHCl_3_ (2 M)	–20	15 h	92	–7/18	71/29
6	**3d**	CHCl_3_ (2 M)	–20	15 h	>95	75/13	88/12
7	**3e**	CHCl_3_ (2 M)	–20	15 h	>95	–73/16	90/10
8	**4**	CHCl_3_ (2 M)	–20	15 h	91	–64/–6	82/18
9	**5**	CHCl_3_ (2 M)	–20	15 h	>95	–71/22	66/34
10	**3d**	CHCl_3_ (0.5 M)	–20	34 h	95	86/–11	91/9
11	**3d**	CH_2_Cl_2_ (0.5 M)	–20	34 h	93	86/–39	93.5/6.5
12	**3d**	PhCH_3_ (0.5 M)	–20	34 h	80	48/–28	80/20
13	**3d**	THF (0.5 M)	–20	34 h	>95	50/–28	79.5/20.5
14	**3d**	Et_2_O (0.5 M)	–20	34 h	>95	53/–28	83/17
15	**3d**	CH_3_CN (0.5 M)	–20	34 h	>95	72/–18	88/12
16^*e*^ ^,^^*f*^	**3d**	CH_2_Cl_2_ (0.1 M)	–50	88 h	>95 (92)^*b*^	94/ND	97.5/2.5

^*a*^Reactions were carried out with 0.1 mmol of **6a** and 0.15 mmol of **7a**.

^*b*^Isolated yield.

^*c*^Determined by chrial HPLC analysis.

^*d*^ee (*anti*/*syn*).

^*e*^10 mg of 4 Å molecular sieves were added.

^*f*^15 mol% of **3d**.

Applying the optimized reaction conditions for the model reaction, we investigated the substrate scope of this asymmetric aldol reaction (Table 2). The reactions of aldehyde **7a** and azlactones **6a–g** bearing different α-alkyl substituents gave consistently excellent yields, enantioselectivity and *anti*-selective diastereoselectivity (entries 1–7, Table 2). The catalyst could also accommodate variations in aliphatic aldehydes as shown by its high efficiency in the promotion of asymmetric aldol reactions involving a series of aliphatic aldehydes (entries 8–11, Table 2). The tolerance of aldehyde **7d**, which bears a linear C12 alkyl chain, is noteworthy. With catalyst **3e**, the reaction provide equally efficient access to the other enantiomer of the aldol product, as shown in the formation of aldol adduct *ent*-**8ba**, *ent*-**8bc** and *ent*-**8dc** (entries 2, 9, 10, Table 2). As detailed in the ESI,† the relative and absolute configurations of aldol products **8** were determined by 1D NOESY experiment and a modified Mosher's method, respectively.^16^

**Table 2:** Scope of reaction^*a*^ ^*b*^ ^*e*^

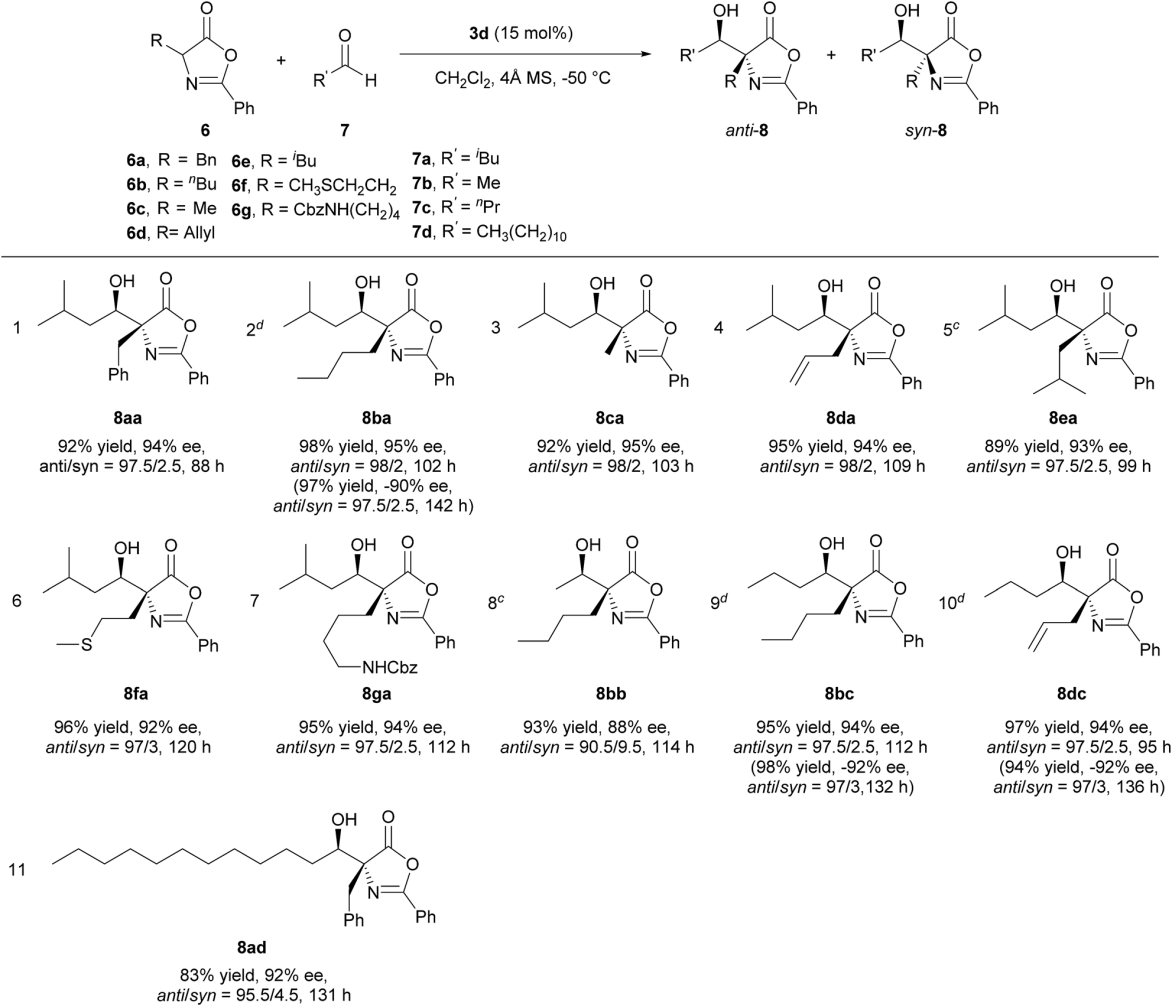

^*a*^Unless noted, reactions were carried out with 0.1 mmol of **6**, 0.15 mmol of **7**, 0.015 mmol of **3d**, 10 mg of 4 Å molecular sieves in 1 mL of dichloromethane.

^*b*^ee value and *anti*/*syn* ratio determined by chiral HPLC analysis.

^*c*^0.2 mmol of **7b**.

^*d*^Results in parentheses obtained using **3e** (15 mol%) as catalyst.

^*e*^See ESI for determination of relative and absolute configurations.

To demonstrate the potential synthetic utility of the chiral aldol adduct **8**, ring opening transformations converting **8** into useful β-hydroxy-α-amino acid derivative **10** must be developed. We found that **8** were liable toward retro-aldol initiated decompositions under a variety of reaction conditions. After extensive experimental explorations, we were able to establish a high yield, three-step protocol to convert **8** into β-hydroxy-α-aminoester **10** (Scheme 2). Critical to the development of this useful conversion was the experimental discovery that the THP protected β-hydroxy-α-alkylazlactones **9**, unlike **8**, is inert toward retro-aldol decompositions.^17^ It should be noted that the four-step enantioselective preparations of β-hydroxy-α-aminoester **10** from azlactones **6** and aldehydes **7** require only a single purification for the isolation of **10**, both intermediates **8** and **9** were used for the next step without subjecting to purifications. To establish enantioselective access to all four stereoisomers of β-hydroxy-α-amino acid derivative **10**, we developed a one-pot conversion of *anti*-β-hydroxy-α-amino acid **10da** into the corresponding *syn*-β-hydroxy-α-amino acid *syn*-**12da** involving the treatment of *anti*-**10da** with thionyl chloride followed by HCl in THF (Scheme 2).

**Scheme 2:**
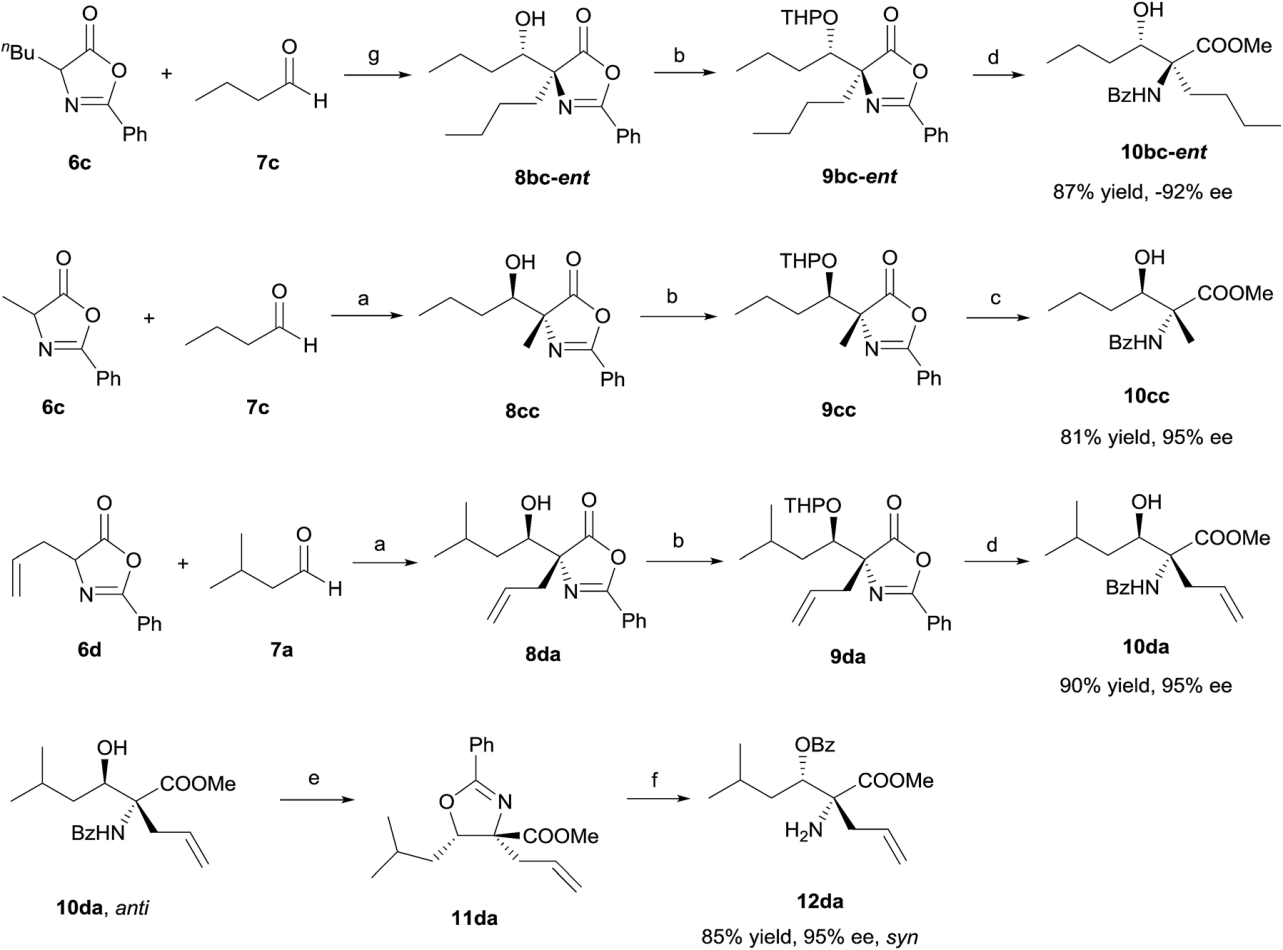
Transformation of aldol product **8**. Reagents and conditions: (a) **3d** (15 mol%), CH_2_Cl_2_, 4 Å MS, –50 °C; (b) PPTS, DHP, CH_2_Cl_2_, rt; then K_2_CO_3_, Na_2_SO_4_, MeOH, rt; (c) 2 N HCl, MeOH, rt; (d) HCl in MeOH (∼1.25 M), rt; (e) SOCl_2_, THF, rt; (f) 2 N HCl, THF, rt; (g) **3e** (15 mol%), CH_2_Cl_2_, 4 Å MS, –50 °C. PPTS = pyridinum-*p*-toluenesulfonate; DHP = 3,4-dihydro-2-*H*-pyran.

## Conclusions

In summary, we have developed a highly enantioselective and diastereoselective direct aldol reaction of α-alkyl azlactones with aliphatic aldehydes catalyzed by cinchona alkaloid catalysts **3d** and **3e**. To our knowledge, this is the first efficient asymmetric direct aldol reaction of azlactones and aliphatic aldehydes. Providing an efficient catalytic asymmetric access to β-hydroxy-α-amino acids bearing alkyl substituents at both the tertiary β-stereocenter and the quaternary α-stereocenter, this new catalytic asymmetric aldol reaction should find applications in natural product synthesis and medicinal chemistry.^18^

## Supplementary Material

Supplementary information

## References

[cit1] Williams D. H. (1984). Acc. Chem. Res..

[cit2] Kato T., Hinoo H., Terui Y., Kikuchi J., Shoji J. (1988). J. Antibiot..

[cit3] Schreiber S. L. (1991). Science.

[cit4] Fujita T., Inoue K., Yamamoto S., Ikumoto T., Sasaki S., Toyama R., Chiba K., Hoshino Y., Okumoto T. (1994). J. Antibiot..

[cit5] Lotz B. T., Miller J. (1993). J. Org. Chem..

[cit6] Pansare S. V., Vederas J. C. (1987). J. Org. Chem..

[cit7] Tanner D. (1994). Angew. Chem., Int. Ed..

[cit8] Ito Y., Sawamura M., Hayashi T. (1986). J. Am. Chem. Soc..

[cit9] Horikawa M., Busch-Peterson J., Corey E. J. (1999). Tetrahedron Lett..

[cit10] Vassilev V. P., Uchiyama T., Kajimoto T., Wong C.-H. (1995). Tetrahedron Lett..

[cit11] Tao B., Schlingloff G., Sharpless K. B. (1998). Tetrahedron Lett..

[cit12] Noyori R., Ikeda T., Ohkuma T., Widhalm M., Kitamura M., Takaya H., Akutagawa S., Sayo N., Saito T., Taketomi T., Kumobayashi H. (1989). J. Am. Chem. Soc..

[cit13] Trost B. M., Ariza X. (1997). Angew. Chem., Int. Ed..

[cit14] Terada M., Tanaka H., Sorimachi K. (2009). J. Am. Chem. Soc..

[cit15] Shibata K., Shingu K., Vassilev V. P., Nishide K., Fujita T., Node M., Kajimoto T., Wong C.-H. (1996). Tetrahedron Lett..

[cit16] Please see ESI for details

[cit17] Miyashita M., Yoshikoshi A., Grieco P. A. (1977). J. Org. Chem..

[cit18] Strader C. R., Pearce C. J., Oberlies N. H. (2011). J. Nat. Prod..

