# Supplementary material for: Catalytic asymmetric direct aldol reaction of α-alkyl azlactones and aliphatic aldehydes
Source: Chem Sci. 2015 Aug 4;6(11):6510–4. doi: 10.1039/c5sc02116b (PMC4762611; doi:10.1039/c5sc02116b)
Supplement: Supplementary file 1 [file SC-006-C5SC02116B-s001.pdf]

Electronic Supplementary Material (ESI) for Chemical Science.
This journal is © The Royal Society of Chemistry 2015

Supporting Information

Catalytic Asymmetric Direct Aldol Reaction of *α*-Alkyl Azlactones and Aliphatic Aldehydes

Yang Zheng and Li Deng*

Department of Chemistry

Brandeis University

Waltham, Massachusetts 02454-9110, United States

Table of contents:

1. General information……………………………………………………………………..1
2. Materials and methods……………………………………………………………………1
3. Aldol reaction of azlactones and aliphatic aldehydes……………………………………….5
4. Transformation of Aldol products……………………………………………………….13
5. Determination of relative configurations…………………………………………………18
6. Determination of absolute configurations………………………………………………...21
7. References………………………………………………………………………………..23
8. ^{1}H and ^{13}C NMR Spectra, HPLC chromatograms……………………………………….24

1. General information.

^{1}H and ^{13}C NMR spectra were recorded on a Varian instrument (400 MHz and 100 MHz, respectively) and internally referenced to tetramethylsilane signal or residual protio solvent signals. Data for ^{1}H NMR are recorded as follows: chemical shift (δ, ppm), multiplicity (s, singlet; d, doublet; t, triplet; q, quartet; m, multiplet), integration, coupling constant (Hz). Data for ^{13}C NMR are reported in terms of chemical shift (δ, ppm). Infrared spectra were recorded on a Perkin Elmer FT-IR Spectrometer and are reported in frequency of absorption. Low resolution and high resolution mass spectra were recorded on either a Micromass 70-VSE-B instrument (EI, CI) or a Micromass Q-TOF instrument (ESI). Specific rotations were measured on a Jasco Digital Polarimeter.

High performance liquid chromatography (HPLC) analyses were performed on a Hewlett Packard 1100 Series instrument equipped with a quaternary pump, using Daicel Chiralpak AD, AS, AD-H and AS-H columns, Daicel Chiralcel OJ, OJ-H Columns. UV absorption was monitored at 254 nm.

2. Materials and methods

A. Aliphatic aldehydes:

Aldehydes **7a-c** were purchased from Sigma-Aldrich and were distilled before use. Aldehyde **7d** was purchased from Alfa Aesar and was purified by flash column chromatography before use.

B. 4-Alkyl-2-phenyl-5-(*4H*)-oxazolones (azlactones):

All natural and unnatural amino acids were purchased from Sigma Aldrich and used as received. Benzoyl chloride was purchased from Sigma Aldrich and used without out further

purification. EDCI was purchased from TCI and used without further purification. Thionyl chloride was purchased from Sigma Aldrich and distilled before use.

General procedure for the preparation of *N*-benzoyl amino acids:

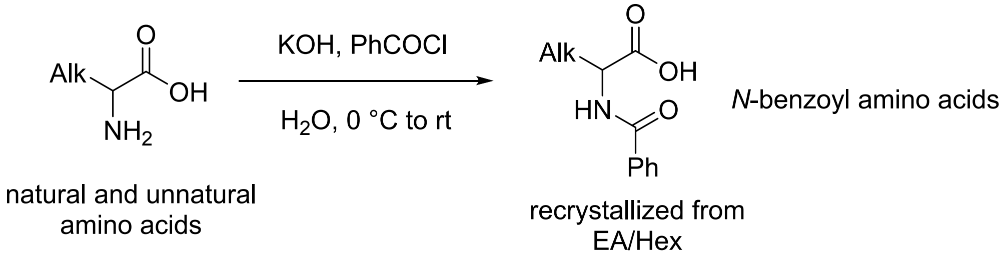

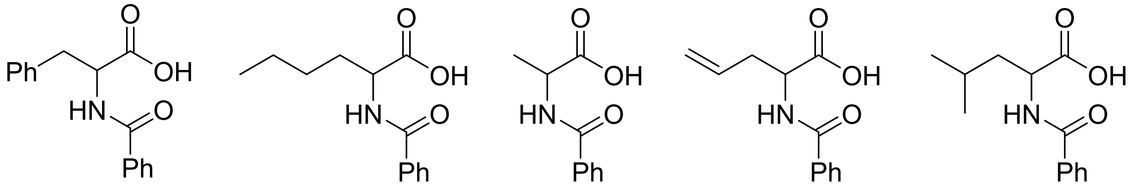

S1a **S1b** **S1c** **S1d** **S1e**

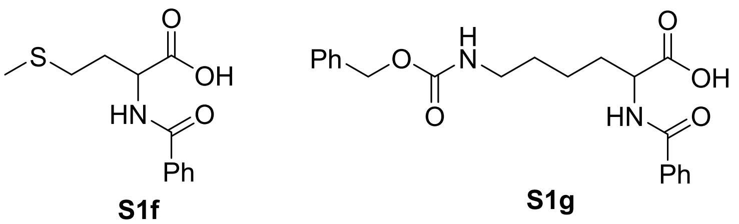

S1f **S1g**

To the mixture of a DL-amino acid (10 mmol) and KOH (23 mmol, 2.3 eq) in water was added benzoyl chloride (11 mmol, 1.1 eq) at 0 °C dropwise. The reaction was allowed to warm to room temperature and was stirred for one day. The reaction mixture was acidified with 6N HCl carefully to adjust the pH to 1 and was then extracted with EA. The combined organic phase was evaporated and the crude solid product was recrystallized from EA/Hex to give the corresponding *N*-benzoyl amino acid. All spectroscopic data were in accordance with reported literature data[1-5].

General procedure for the preparation of azlactones:

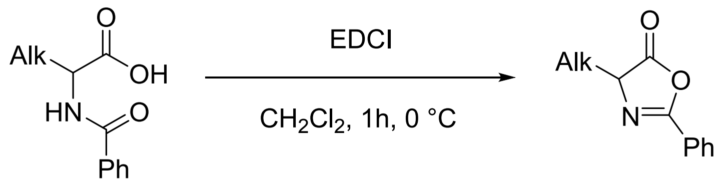

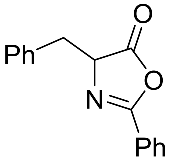

6a

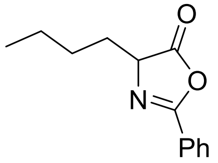

6b

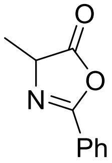

6c

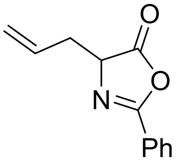

6d

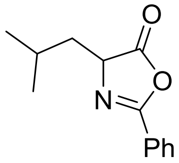

6e

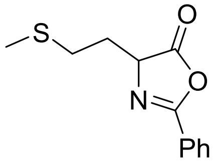

6f

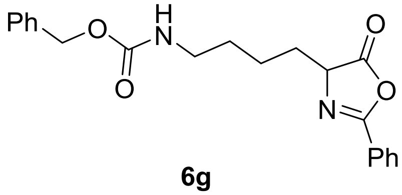

6g

To the stirred suspension of an *N*-benzoyl amino acid (0.5 mmol) in CH_2Cl_2 (5 mL, 0.1 M) cooled in an ice bath was added EDCI in one portion (0.5 mmol, 1 eq). After 1h, the reaction was diluted with 20 mL of Et_2O, washed with water (15 mL x 2) and brine (10 mL). The combined organic phase was dried over $MgSO_4$, filtered and evaporated to give the corresponding azlactone in reasonably pure form without further purification. The spectroscopic data of all azlactones (**6a-h**) were in accordance with reported literature data[6-8].

C. Catalysts

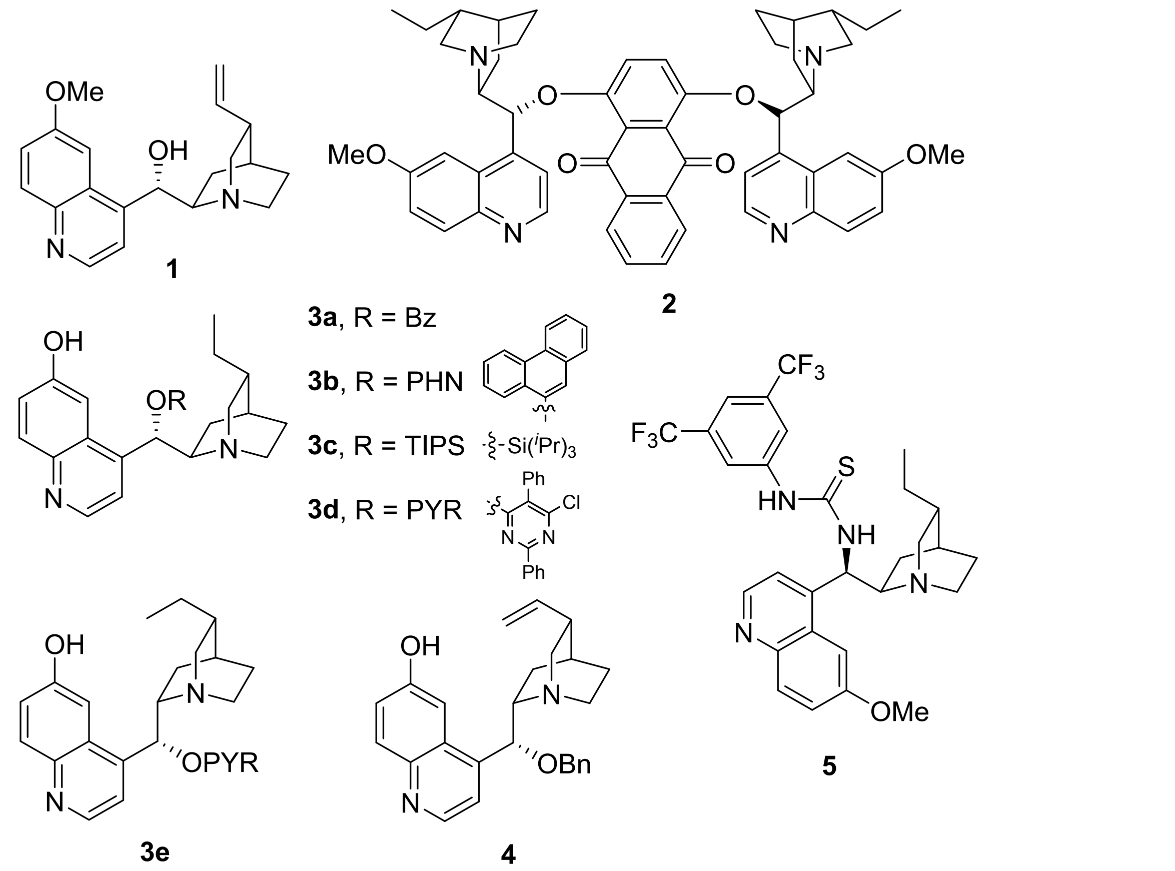

Catalysts **1** and **2** were purchased from Sigma Aldrich and used without further purification. All the other catalysts were synthesized according to literature procedures[9-14].

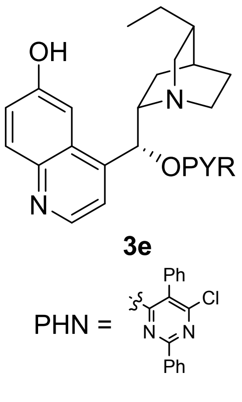

Catalyst **3e** was obtained as a white solid. $[\alpha]_D^{20}$ = +312.3 (c = 1.22, CH_2Cl_2). **^{1}H NMR** (400 MHz, $CDCl_3$): δ = 9.45 (br, 1H), 8.74 – 8.49 (m, 2H), 7.98 (d, *J* = 9.0 Hz, 1H), 7.88 (d, *J* = 6.4 Hz, 2H), 7.59 (t, *J* = 7.3 Hz, 2H), 7.51 (t, *J* = 9.2 Hz, 3H), 7.26 (d, *J* = 9.5 Hz, 1H), 7.23 – 7.14 (m, 2H), 7.04 – 6.92 (m, 3H), 3.18 – 2.98 (m, 3H), 2.74 – 2.56 (m, 1H), 2.27 (d, *J* = 11.8 Hz, 1H), 1.81 – 1.67 (m, 1H), 1.59 (s, 1H), 1.53 – 1.30 (m, 2H), 1.20 – 1.01 (m, 3H), 0.89 – 0.76 (m, 1H), 0.69 (t, *J* = 7.3 Hz, 3H). **^{13}C NMR** (100 MHz, $CDCl_3$): δ = 166.13, 162.84, 159.82, 157.22, 146.46, 144.08, 143.72, 135.26, 132.31, 131.27, 131.08, 130.02, 128.77, 128.74, 128.43, 128.25, 127.41, 123.18, 118.69, 117.27, 107.71, 77.48, 77.16, 76.84, 76.53, 58.60, 58.37, 43.50, 36.89, 27.35, 26.73, 25.01, 20.54, 11.94. **IR**

(neat): ν = 3054, 2957, 2953, 2877, 2360, 2338, 1619, 1571, 1515, 1407, 1377, 1264,1210, 1030, 999, 846, 809, 735, 700 cm^{-1}. **HRMS** (ESI/$[M+H]^+$) = Calcd. for $C_{26}H_{29}N_2O_3$ m/z = 577.2370, found m/z = 577.2368.

D. The other materials:

Molecular Sieves (4Å, purchased from Alfa Aesar) were dried in a round-bottom flask under vacuum with a Bunsen burner. Triethylamine, dihydropyran (DHP) and pyridinium *p*-toluenesulfonate (PPTS) were purchased from Alfa Aesar and used without further purification. HCl in MeOH (~1.25 M) was purchased from Sigma Aldrich and was used without further purification. Thionyl chloride was purchased from Sigma Aldrich and distilled before use. 4-(Dimethylamino)pyridine (DMAP) was purchased from Sigma Aldrich and used as received.

3. Aldol reaction of azlactones and aliphatic aldehydes

General procedure for racemic reactions (Table 1 & 2):

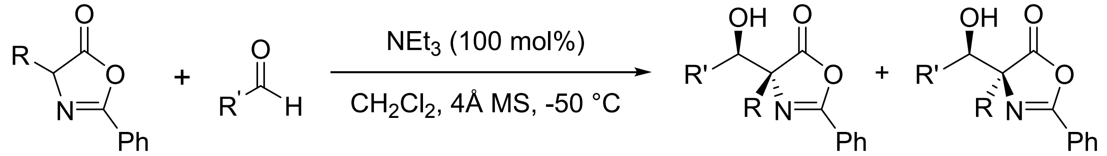

6 **7** *anti*-**8** *syn*-**8**

Freshly prepared azlactone **6** (0.5 mmol), freshly distilled or purified aldehyde **7** (1 mmol) and freshly dried 4Å Molecular Sieves (50 mg) were added to a vial (22 mL), followed by CH_2Cl_2 (5 mL). The vial was gently shaken and then placed in a -50 °C freezer. After 15 minutes, triethylamine (70 µL) was added by a micro syringe. The vial was gently shaken again and was placed back to the -50 °C freezer. The reaction progress was monitored by TLC. Usually it takes 3 days to complete. The reaction mixture was passed through a short pad of silica gel with washing by Et_2O. The filtrate was concentrated to give a crude product which was suitable

for HPLC analysis and further transformations. All crude products were stored in a -50 °C freezer due to instability.

General procedure for asymmetric reactions (Table 1 & 2):

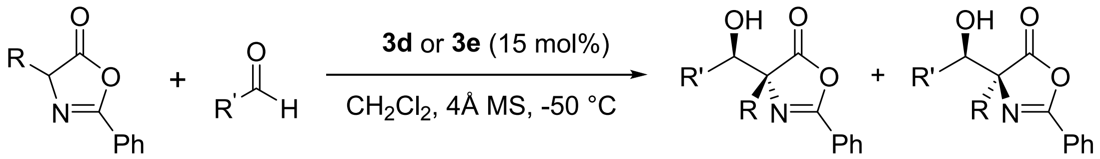

6 7 *anti*-8 *syn*-8

Freshly prepared azlactone **6** (0.1mmol), catalyst **3d** or **3e** (8.7 mg, 0.015 mmol) and freshly dried 4Å Molecular Sieves (10 mg) were added to a vial (2 mL), followed by CH_2Cl_2 (1 mL). The vial was gently shaken and then placed in a -50 °C freezer. After 15 minutes, freshly distilled or purified aldehyde **7** (0.15 mmol) was added by a micro syringe. The vial was gently shaken again and was placed back to the -50 °C freezer. The reaction progress was monitored by TLC (All reaction times in Table 1 and 2 were not optimized). The reaction mixture was passed through a short pad of silica gel with washing by Et_2O. The filtrate was concentrated to give the crude product, which was subject to vacuum to remove solvent and aldehyde residues to give product **8** in reasonably pure form. Product **8ad** was an exception, which was purified by a short deactivated silica gel column (Hex to 1/20 = EA/Hex). All products were stored in a -50 °C freezer due to instability.

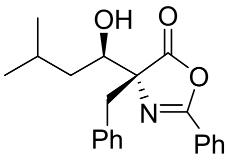

8aa

92% yield, 94% ee,
anti/*syn* = 97.5/2.5, 88 h

Product **8aa** was obtained as a colorless oil (31.2 mg) from a reaction catalyzed by **3d**. Enantiomeric excess and diastereometric ratio were determined by HPLC analysis: Daicel Chiralcel AS-H, Hexanes/IPA = 95/5, 1.0 ml/min, λ = 254 nm, t (*anti*, minor) = 13.91 min, t (*anti*, major) = 16.59 min, t (*syn*, major) = 25.53, t (*syn*, minor) = 39.98. $[\alpha]_D^{20}$ = +129.3 (c = 0.31, CH_2Cl_2). **^{1}H NMR** (400 MHz, $CDCl_3$): δ = 7.78 (d, *J* = 7.7 Hz, 2H), 7.54 – 7.45 (m, 1H), 7.38 (t, *J* = 7.7 Hz, 2H), 7.19 – 7.04 (m, 5H), 4.07 (dd, *J* = 10.3, 5.5 Hz, 1H), 3.38 (d, *J* = 13.4 Hz, 1H), 3.25 (d, *J* = 13.4 Hz, 1H), 2.66 (d, *J* = 6.6 Hz, 1H), 2.00 – 1.84 (m, 1H), 1.73 –

1.62 (m, 1H), 1.36 – 1.26 (m, 1H), 0.96 (dd, *J* = 11.3, 6.6 Hz, 6H). **^{13}C NMR** (100 MHz, CDCl3): δ = 178.00, 161.37, 134.21, 132.84, 130.26, 128.79, 128.26, 127.97, 127.29, 125.36, 78.93, 73.12, 40.15, 40.12, 24.63, 24.00, 21.26. **IR** (neat): ν = 3242, 2949, 2925, 2601, 2496, 2360, 1818, 1724, 1649, 1602, 1581, 1496, 1450, 1367, 1385, 1322, 1337, 1292, 1238, 1212, 1171, 1141, 1086, 1058, 977, 924, 902, 883, 858, 808, 776, 743, 698, 643, 620, 611 cm^{-1}. **HRMS** (ESI/[M+H]$^+$) = Calcd. for $C_{21}H_{23}NO_3$ m/z = 338.1756, found m/z = 338.1752.

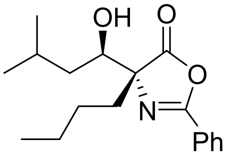

8ba

98% yield, 95% ee,
anti/*syn* = 98/2, 102 h

Product **8ba** was obtained as a colorless oil (29.6 mg) from a reaction catalyzed by **3d**. Enantiomeric excess and diastereometric ratio were determined by HPLC analysis: Daicel Chiralpak AD-H connected with AD, Hexanes/IPA = 95/5, 0.8 ml/min, λ = 254 nm, t (*anti*, minor) = 17.02 min, t (*syn*, major) = 18.38 min, t (*anti*, major) = 19.24, t (*syn*, minor) = 22.86. $[\alpha]_D^{20}$ = +13.1 (c = 0.36, CH_2Cl_2). **^{1}H NMR** (400 MHz, $CDCl_3$): δ = 7.96 (d, J = 8.0 Hz, 2H), 7.57 (t, J = 7.3 Hz, 1H), 7.46 (t, J = 7.7 Hz, 2H), 3.95 (d, J = 10.8 Hz, 1H), 2.67 (s, 1H), 2.08 – 1.79 (m, 3H), 1.72 – 1.58 (m, 1H), 1.37 – 1.04 (m, 5H), 0.94 (dd, J = 12.4, 6.6 Hz, 6H), 0.85 (t, J = 7.3 Hz, 3H). **^{13}C NMR** (100 MHz, $CDCl_3$) δ = 178.71, 161.56, 132.97, 128.94, 128.14, 125.55, 77.95, 73.06, 39.50, 33.74, 25.99, 24.56, 24.00, 22.74, 21.21, 13.95. **IR** (neat): ν = 3255, 2959, 2944, 2863, 1820, 1642, 1464, 1451, 1365, 1342, 1323, 1289, 1261, 1184, 1153, 1077, 1067, 986, 963, 889, 854, 840, 797, 778, 734, 697, 684, 614 cm^{-1}. **HRMS** (ESI/[M+H]$^+$) = Calcd. for $C_{18}H_{26}NO_3$ m/z = 304.1913, found m/z = 304.1909.

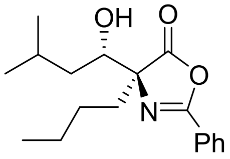

ent-**8ba**

97% yield, -90% ee,
anti/*syn* = 97.5/2.5, 142 h

Product *ent*-**8ba** was obtained as a colorless oil (29.5 mg) from a reaction catalyzed by **3e**. Enantiomeric excess and diastereometric ratio were determined by HPLC analysis: Daicel Chiralpak AD-H connected with AD, Hexanes/IPA = 95/5, 0.8 ml/min, λ = 254 nm, t (*anti*, major) = 17.57 min, t (*syn*, minor) = 18.85 min, t (*anti*, minor) = 19.66, t (*syn*, major) = 23.30. $[\alpha]_D^{20}$ = +10.7 (c = 0.37, CH_2Cl_2). All the other data were the same as product **8ba**.

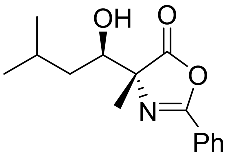

8ca

92% yield, 95% ee, *anti*/*syn* = 98/2, 103 h

Product **8ca** was obtained as a colorless oil (24 mg) from a reaction catalyzed by **3d**. Enantiomeric excess and diastereometric ratio were determined by HPLC analysis: Daicel Chiralpak AD-H, Hexanes/IPA = 99/1, 1.0 ml/min, λ = 254 nm, t (*anti*, minor) = 27.84 min, t (*anti*, major) = 31.96 min, t (*anti*, major) = 37.02, t (*syn*, minor) = 38.29. $[\alpha]_D^{20}$ **=** +10.1 (c = 0.33, CH_2Cl_2). **^{1}H NMR** (400 MHz, $CDCl_3$): δ = 8.00 (d, J = 7.4 Hz, 1H), 7.59 (t, J = 7.4 Hz, 1H), 7.49 (t, J = 7.6 Hz, 2H), 3.90 (dd, J = 10.5, 4.0 Hz, 1H), 2.20 (s, 1H), 1.95 – 1.78 (m, 1H), 1.69 – 1.58 (m, 1H), 1.55 (s, 3H), 1.29 – 1.22 (m, 1H), 0.94 (dd, J = 12.4, 6.6 Hz, 6H). **^{13}C NMR** (100 MHz, $CDCl_3$): δ = 179.00, 161.47, 133.00, 128.93, 128.11, 125.61, 73.62, 73.49, 39.35, 24.59, 23.96, 21.20, 20.48. **IR** (neat): ν = 3290, 2955, 2870, 2359, 2337, 1819, 1739, 1649, 1602, 1581, 1495, 1451, 1368, 1323, 1292, 1216, 1173, 1109, 1094, 1069, 1031, 1000, 957, 929, 885, 849, 780, 738, 696, 613 cm^{-1}. **HRMS** (ESI/$[M+H]^+$) = Calcd. for $C_{15}H_{20}NO_3$ m/z = 262.1443, found m/z = 262.1435.

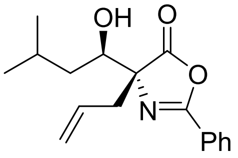

8da

95% yield, 94% ee, *anti*/*syn* = 98/2, 109 h

Product **8ca** was obtained as a light yellow oil (27.4 mg) from a reaction catalyzed by **3d**. Enantiomeric excess and diastereometric ratio were determined by HPLC analysis: Daicel Chiralpak AD-H, Hexanes/IPA = 96/4, 1.0 ml/min, λ = 254 nm, t (*anti*, minor) = 16.41 min, t (*syn*, major) = 18.02 min, t (*anti*, major) = 19.49, t (*syn*, minor) = 26.28. $[\alpha]_D^{20}$ **=** +61 (c = 0.72, CH_2Cl_2). **^{1}H NMR** (400 MHz, $CDCl_3$): δ = 8.00 (d, J = 7.8 Hz, 2H), 7.59 (t, J = 7.4 Hz, 1H), 7.49 (t, J = 7.6 Hz, 2H), 5.62 (td, J = 17.0, 7.4 Hz, 1H), 5.19 (d, J = 17.0 Hz, 1H), 5.10 (d, J = 10.1 Hz, 1H), 3.97 (d, J = 10.3 Hz, 1H), 2.83 (dd, J = 13.6, 6.7 Hz, 1H), 2.67 (dd, J = 13.7, 8.0 Hz, 1H), 2.16 (d, J = 21.5 Hz, 1H), 1.96 – 1.79 (m, 1H), 1.60 (ddd, J = 14.6, 11.1, 3.8 Hz, 1H), 1.25 – 1.15 (m, 1H), 0.93 (dd, J = 11.1, 6.6 Hz, 6H). **^{13}C NMR** (100 MHz, $CDCl_3$): δ = 177.98, 161.62, 133.03, 130.58, 128.93, 128.17, 125.50, 120.80, 77.91, 72.69, 39.69, 38.24, 24.55, 23.99, 21.22. **IR** (neat): ν = 3318, 2955, 2910, 2870, 2359, 1816, 1739, 1648, 1580, 1495, 1647, 1451, 1368, 1322, 1289, 1217, 1178, 1042, 1026, 970, 925, 900, 850, 779, 700,

689, 668 cm^{-1}. **HRMS** (ESI/$[M+H]^+$) = Calcd. for $C_{17}H_{22}NO_3$ m/z = 288.1600, found m/z = 288.1598.

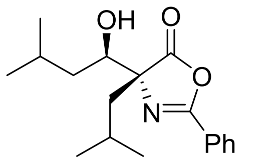

8ea

89% yield, 93% ee, *anti*/*syn* = 97.5/2.5, 99 h

Product **8ea** was obtained as a colorless yellow oil (27 mg) from a reaction catalyzed by **3d**. Enantiomeric excess and diastereometric ratio were determined by HPLC analysis: Daicel Chiralpak AD-H, Hexanes/IPA = 96/4, 1.0 ml/min, λ = 254 nm, t (*anti*, minor) = 12.65 min, t (*anti*, major) = 15.16 min, t (*syn*, major) = 17.11, t (*syn*, minor) = 25.16. $[\alpha]^{20}_{D}$ = +52.7 (c = 0.34, CH_2Cl_2). **^{1}H NMR** (400 MHz, $CDCl_3$): δ = 8.03 – 7.95 (m, 2H), 8.04 – 7.97 (m, 2H), 7.59 (t, J = 7.4 Hz, 1H), 7.49 (t, J = 7.5 Hz, 2H), 3.88 (dd, J = 10.3, 5.5 Hz, 1H), 2.37 – 2.14 (m, 1H), 2.05 (dd, J = 14.0, 5.1 Hz, 1H), 1.92 – 1.77 (m, 2H), 1.70 – 1.52 (m, 2H), 1.30 – 1.19 (m, 1H), 0.95 (d, J = 6.7 Hz, 3H), 0.91 (dd, J = 6.5, 3.9 Hz, 6H), 0.83 (d, J = 6.6 Hz, 3H). **^{13}C NMR** (100 MHz, $CDCl_3$): δ = 179.21, 161.34, 132.95, 128.97, 128.11, 125.62, 77.48, 77.16, 76.85, 74.01, 42.61, 38.89, 24.99, 24.59, 24.33, 24.02, 22.96, 21.18. **IR** (neat): ν = 3289, 2955, 2927, 2870, 1817, 1649, 1580, 1495, 1467, 1450, 1387, 1368, 1323, 1289, 1166, 1078, 1043, 1024, 970, 885, 855, 779, 698, 654, 623, 613, 602 cm^{-1}. **HRMS** (ESI/$[M+H]^+$) = Calcd. for $C_{18}H_{26}NO_3$ m/z = 304.1913, found m/z = 304.1913.

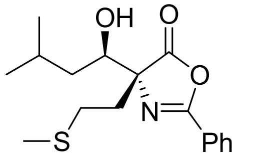

8fa

96% yield, 92% ee, *anti*/*syn* = 97/3, 120 h

Product **8fa** was obtained as a light yellow oil (31 mg) from a reaction catalyzed by **3d**. Enantiomeric excess and diastereometric ratio were determined by HPLC analysis: Daicel Chiralpak AD-H, Hexanes/IPA = 96.5/3.5, 1.0 ml/min, λ = 254 nm, t (*anti*, minor) = 32.10 min, t (*syn*, major) = 34.33 min, t (*syn*, minor) = 44.90, t (*anti*, major) = 47.92. $[\alpha]^{20}_{D}$ = +29.8 (c = 0.4, CH_2Cl_2). **^{1}H NMR** (400 MHz, $CDCl_3$): δ = 7.98 (d, J = 7.6 Hz, 2H), 7.58 (t, J = 7.4 Hz, 1H), 7.48 (t, J = 7.7 Hz, 2H), 3.92 (dd, J = 10.6, 4.2 Hz, 1H), 2.56 (d, J = 6.0 Hz, 1H), 2.51 – 2.23 (m, 4H), 2.04 (s, 3H), 1.94 – 1.76 (m, 1H), 1.68 – 1.58 (m, 1H), 1.25 – 1.15 (m, 1H), 0.93 (dd, J = 14.1, 6.6 Hz, 6H). **^{13}C NMR** (100 MHz, $CDCl_3$): δ = 178.59, 162.23, 133.13, 128.96, 128.18, 125.48, 76.63, 73.30, 39.25, 32.71, 28.91, 24.53, 23.96, 21.19, 15.21. **IR** (neat): ν =

3460, 2956, 2911, 2869, 2360, 2337, 1812, 1739, 1649, 1580, 1495, 1451, 1367, 1321, 1292, 1216, 1139, 1070, 1066, 1009, 1001, 963, 908, 881, 850, 780, 730, 695, 647, 609 cm^{-1}. **HRMS** (ESI/$[M+H]^+$) = Calcd. for $C_{17}H_{24}NO_3S$ m/z = 322.1477, found m/z = 322.1478.

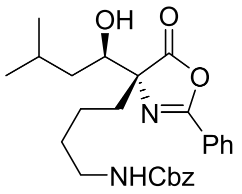

8ga

95% yield, 94% ee, *anti*/*syn* = 97.5/2.5, 112 h

Product **8ga** was obtained as a colorless oil (47 mg) from a reaction catalyzed by **3d**. Enantiomeric excess and diastereometric ratio were determined by HPLC analysis: Daicel Chiralpak AD-H, Hexanes/IPA = 87/13, 1.0 ml/min, λ = 254 nm, t (*anti*, minor) = 26.73 min, t (*anti*, major) = 32.71 min, t (*syn*, minor) = 36.01, t (*syn*, major) = 37.89. $[\alpha]_D^{20}$ = +12.1 (c = 0.56, CH_2Cl_2). **^{1}H NMR** (400 MHz, $CDCl_3$): δ = 7.98 (d, J = 7.4 Hz, 2H), 7.57 (t, J = 7.4 Hz, 1H), 7.47 (t, J = 7.6 Hz, 2H), 7.38 – 7.27 (m, 5H), 5.05 (s, 2H), 4.79 (s, 1H), 3.92 (d, J = 10.3 Hz, 1H), 3.26 – 2.97 (m, 2H), 2.63 (s, 1H), 2.11 – 1.75 (m, 3H), 1.67 – 1.55 (m, 1H), 1.55 – 1.39 (m, 2H), 1.24 – 1.04 (m, 3H), 0.91 (dd, J = 12.2, 6.6 Hz, 6H). **^{13}C NMR** (100 MHz, $CDCl_3$): δ = 178.57, 161.60, 156.41, 136.57, 133.06, 128.95, 128.59, 128.18, 128.14, 125.43, 77.75, 72.94, 66.71, 40.63, 39.59, 33.40, 29.81, 24.50, 23.94, 21.17, 21.05. **IR** (neat): v = 3334, 2954, 2910, 2867, 1813, 1697, 1650, 1580, 1526, 1496, 1452, 1368, 1321, 1288, 1254, 1178, 1134, 1044, 1022, 972, 882, 850, 777, 736, 694, 606 cm^{-1}. **HRMS** (ESI/$[M+H]^+$) = Calcd. for $C_{26}H_{33}N_2O_5$ m/z = 453.2389, found m/z = 453.2390.

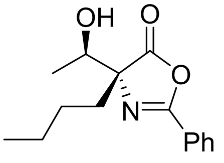

8bb

93% yield, 88% ee, *anti*/*syn* = 90.5/9.5, 114 h

Product **8bb** was obtained as a colorless oil (24.2 mg) from a reaction catalyzed by **3d**. Enantiomeric excess and diastereometric ratio were determined by HPLC analysis: Daicel Chiralcel OJ-H connected with OJ, Hexanes/IPA = 96/4, 0.8 ml/min, λ = 254 nm, t (*anti*, minor) = 40.78 min, t (*anti*, major) = 59.11 min, t (*syn*, minor) = 63.58, t (*syn*, minor) = 73.38. $[\alpha]_D^{20}$ = +1.2 (c = 1.2, CH_2Cl_2). **^{1}H NMR** (400 MHz, $CDCl_3$): δ = 8.01 (d, J = 7.3 Hz, 2H), 7.59 (t, J = 7.3 Hz, 1H), 7.49 (t, J = 7.6 Hz, 2H), 4.16 – 3.97 (m, 1H), 2.37 (s, 1H), 2.07 – 1.85 (m, 2H), 1.41 – 1.02 (m, 7H), 0.86 (t, J = 7.2 Hz, 3H). **^{13}C NMR** (100 MHz, $CDCl_3$): δ = 178.54, 161.73, 133.03, 128.95, 128.17, 125.56, 77.89, 71.18, 33.56, 26.08,

22.73, 16.98, 13.94. **IR** (neat): v = 3392, 2957, 2930, 2872, 2360, 2321, 1813, 1739, 1650, 1602, 1580, 1520, 1494, 1451, 1378, 1321, 1291, 1230, 1198, 1164, 1074, 1037, 1022, 954, 913, 881, 780, 694, 671, 605 cm^{-1}. **HRMS** (ESI/$[M+H]^+$) = Calcd. for $C_{15}H_{20}NO_3$ m/z = 262.1443, found m/z = 262.1443.

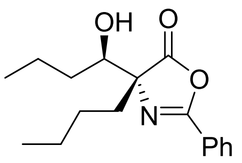

8bc
95% yield, 94% ee,
anti/*syn* = 97.5/2.5, 112 h

Product **8bc** was obtained as a colorless oil (27.5 mg) from a reaction catalyzed by **3d**. Enantiomeric excess and diastereometric ratio were determined by HPLC analysis: Daicel Chiralpak AD-H connected with AD, Hexanes/IPA = 95/5, 0.8 ml/min, λ = 254 nm, t (*anti*, minor) = 62.88 min, t (*anti*, major) = 83.69 min, t (*syn*, major) = 87.09, t (*syn*, minor) = 90.83. $[\alpha]_D^{20}$ = +17.1 (c = 0.35, CH_2Cl_2). ^{1}H NMR (400 MHz, $CDCl_3$): δ = 8.01 (d, *J* = 7.5 Hz, 2H), 7.59 (t, *J* = 7.4 Hz, 1H), 7.49 (t, *J* = 7.6 Hz, 2H), 3.93 – 3.75 (m, 1H), 2.31 – 2.19 (m, 1H), 2.05 (td, *J* = 12.9, 4.8 Hz, 1H), 2.00 – 1.90 (m, 1H), 1.72 – 1.02 (m, 8H), 0.93 (t, *J* = 7.2 Hz, 3H), 0.86 (t, *J* = 7.3 Hz, 3H). **^{13}C NMR** (100 MHz, $CDCl_3$): δ = 178.81, 161.52, 132.97, 128.93, 128.14, 125.58, 77.93, 77.48, 77.16, 76.84, 74.72, 33.68, 32.90, 25.98, 22.74, 19.49, 13.94, 13.92. **IR** (neat): v = 3444, 2958, 2930, 2872, 2337, 1815, 1739, 1649, 1602, 1581, 1495, 1451, 1377, 1322, 1290, 1229, 1217, 1157, 1076, 1041, 1023, 966, 880, 855, 804, 778, 732, 696, 612 cm^{-1}. **HRMS** (ESI/$[M+H]^+$) = Calcd. for $C_{17}H_{24}NO_3$ m/z = 290.1756, found m/z = 290.1760.

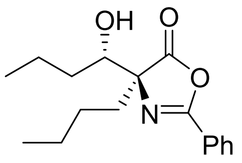

ent-**8bc**
98% yield, -92% ee,
anti/*syn* = 97/3,132 h

Product *ent*-**8bc** was obtained as a colorless oil (28.3 mg) from a reaction catalyzed by **3d**. Enantiomeric excess and diastereometric ratio were determined by HPLC analysis: Daicel Chiralpak AD-H connected with AD, Hexanes/IPA = 95/5, 0.8 ml/min, λ = 254 nm, t (*anti*, major) = 68.47 min, t (*anti*, minor) = 94.59 min, t (*syn*, major) = 97.69, t (*syn*, minor) = 101.46. All the other data were the same as product **8bc**.

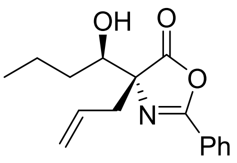

8dc

97% yield, 94% ee,
anti/*syn* = 97.5/2.5, 95 h

Product **8dc** was obtained as a yellow oil (26.5 mg) from a reaction catalyzed by **3d**. Enantiomeric excess and diastereometric ratio were determined by HPLC analysis: Daicel Chiralcel OJ-H, Hexanes/IPA = 97/3, 1.0 ml/min, λ = 254 nm, t (*anti*, minor) = 18.09 min, t (*anti*, major) = 21.01 min, t (*syn*, minor) = 26.48, t (*syn*, minor) = 34.97. $[\alpha]_D^{20}$ = +26.9 (c = 0.33, CH_2Cl_2). **^{1}H NMR** (400 MHz, $CDCl_3$): δ = 8.02 – 7.95 (m, 2H), 7.59 (t, *J* = 6.8 Hz, 1H), 7.53 – 7.44 (m, 2H), 5.62 (td, *J* = 17.0, 7.8 Hz, 1H), 5.19 (d, *J* = 17.0 Hz, 1H), 5.10 (d, *J* = 10.1 Hz, 1H), 3.93 – 3.82 (m, 1H), 2.85 (dd, *J* = 13.6, 6.5 Hz, 1H), 2.68 (dd, *J* = 13.6, 7.9 Hz, 1H), 2.45 – 2.19 (m, 1H), 1.69 – 1.51 (m, 2H), 1.51 – 1.30 (m, 2H), 0.93 (t, *J* = 7.2 Hz, 3H). **^{13}C NMR** (100 MHz, $CDCl_3$): δ = 178.00, 161.61, 133.04, 130.59, 128.92, 128.16, 125.47, 120.80, 77.92, 77.48, 77.16, 76.84, 74.28, 38.17, 33.07, 19.41, 13.91. **IR** (neat): ν = 3400, 2960, 2900, 2848, 1815, 1648, 1580, 1494, 1451, 1322, 1289, 1179, 1156, 1042, 1024, 969, 926, 889, 779, 690 cm^{-1}. **HRMS** (ESI/$[M+H]^+$) = Calcd. for $C_{16}H_{20}NO_3$ m/z = 274.1443, found m/z = 274.1446.

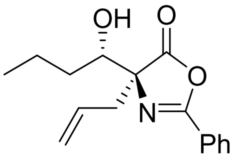

ent-**8dc**

94% yield, -92% ee,
anti/*syn* = 97/3, 136 h

Product *ent*-**8dc** was obtained as a yellow oil (25.8 mg) from a reaction catalyzed by **3d**. Enantiomeric excess and diastereometric ratio were determined by HPLC analysis: Daicel Chiralcel OJ-H, Hexanes/IPA = 97/3, 1.0 ml/min, λ = 254 nm, t (*anti*, major) = 18.21 min, t (*anti*, minor) = 20.54 min, t (*syn*, major) = 25.48, t (*syn*, minor) = 35.65. $[\alpha]_D^{20}$ = -26.2 (c = 0.33, CH_2Cl_2). All the other data were the same as product **8dc**.

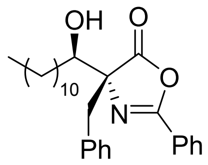

8ad

83% yield, 92% ee,
anti/*syn* = 95.5/4.5, 131 h

Product **8ad** was obtained as a colorless oil (36 mg) from a reaction catalyzed by **3d**. Enantiomeric excess and diastereometric ratio were determined by HPLC analysis: Daicel Chiralcel AS-H, Hexanes/IPA = 96.5/3.5, 1.0 ml/min, λ = 254 nm, t (*anti*, minor) = 16.62 min, t (*anti*, major) = 20.50 min, t (*syn*, major) = 29.36, t (*syn*, minor) = 44.78. $[\alpha]_D^{20}$ = +74 (c = 0.45, CH_2Cl_2). **^{1}H NMR** (400 MHz, $CDCl_3$): δ = 7.81 (d, *J* = 7.5 Hz, 2H), 7.51 (t, *J* = 7.5 Hz, 1H), 7.39 (t, *J* = 7.6 Hz, 2H), 7.17 – 7.05 (m, 5H), 3.91

(dd, J = 12.5, 6.7 Hz, 1H), 3.39 (d, J = 13.4 Hz, 1H), 3.23 (d, J = 13.4 Hz, 1H), 2.16 (d, J = 7.2 Hz, 1H), 1.65 – 1.47 (m, 5H), 1.22 (s, 20H), 0.85 (t, J = 6.7 Hz, 3H). **^{13}C NMR** (100 MHz, $CDCl_3$): δ = 178.05, 161.35, 134.23, 132.84, 130.27, 128.78, 128.25, 127.97, 127.27, 125.37, 78.94, 77.48, 77.16, 76.84, 74.97, 40.10, 32.04, 31.48, 29.75, 29.72, 29.70, 29.48, 26.32, 22.82, 14.27. **IR** (neat): ν = 3293, 2922, 2852, 1816, 1714, 1650, 1581, 1495, 1452, 1322, 1292, 1237, 1171, 1056, 973, 893, 777, 696, 611 cm^{-1}. **HRMS** (ESI/[M+H]$^+$) = Calcd. for $C_{16}H_{20}NO_3$ m/z = 436.2852, found m/z = 436.2852.

4. Transformation of Aldol products

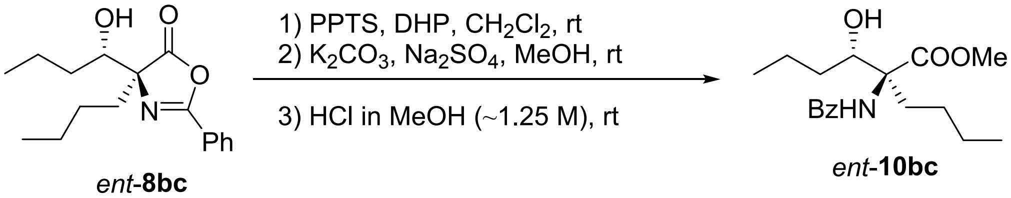

ent-**8bc**

ent-**10bc**
87% yield, -92% ee

Aldol product *ent*-**8bc** was made according to the general procedure reported in previous section. To a 4 mL vial containing Aldol product *ent*-**8bc** (from 0.1 mmol scale reaction) were added dihydropyran (DHP, 0.14 mL, 1.5 mmol, 15 eq), CH_2Cl_2 (0.5 mL) and pyridinium *p*-toluenesulfonate (PPTS, 25.1 mg, 0.1 mmol, 1 eq) sequentially. The reaction mixture was stirred at room temperature for 20 h. After protection with DHP was complete, anhydrous Na_2SO_4 (100 mg), anhydrous MeOH (0.5 mL) and K_2CO_3 (34.6 mg, 0.25 mmol, 2.5 eq) were added to the reaction mixture. After 16 h, HCl in MeOH (1 mL, ~1.25 M) was added. After stirring at room temperature for 19.5 h, the reaction was concentrated and purified by flash column chromatography (1/10 = EA/Hex to 1/5) to give *ent*-**10bc** as a colorless oil (28 mg, 87% yield based on a 0.1 mmol Aldol reaction). All reaction times were not optimized. Racemic product was obtained according to the same procedure. Enantiomeric excess of *ent*-**10bc** was determined by HPLC analysis: Daicel Chiralpak AD-H, Hexanes/IPA = 90/10, 1.0 ml/min, λ = 254 nm, t (*anti*, major) = 9.17 min, t (*anti*, minor) = 10.23 min. $[\alpha]_D^{20}$ = -11.1 (c = 0.35, CH_2Cl_2). **^{1}H NMR** (400 MHz, $CDCl_3$): δ = 7.85 (d, J = 7.4 Hz, 2H), 7.70 (s, 1H), 7.57 (t, J = 7.3 Hz, 1H), 7.49 (t, J = 7.5 Hz, 2H), 5.67 (d, J = 10.9 Hz, 1H), 4.06 – 3.96 (m, 1H), 3.86 (s, 3H), 2.66 (td, J

= 14.0, 3.9 Hz, 1H), 2.01 – 1.87 (m, 1H), 1.61 (tt, *J* = 11.4, 5.9 Hz, 1H), 1.46 – 1.17 (m, 6H), 1.13 – 0.92 (m, 2H), 0.86 (q, *J* = 7.0 Hz, 6H). **^{13}C NMR** (100 MHz, $CDCl_3$): δ = 174.21, 167.93, 133.78, 132.29, 128.92, 127.29, 75.36, 71.10, 53.63, 36.33, 32.22, 26.56, 22.66, 19.32, 14.05, 14.04. **IR** (neat): v = 3396, 2957, 2931, 2872, 1729, 1645, 1602, 1579, 1516, 1485, 1440, 1339, 1290, 1249, 1215, 1185, 1128, 1074, 1016, 960, 914, 866, 822, 711, 690, 664 cm^{-1}. **HRMS** (ESI/[M+H]$^+$) = Calcd. for $C_{18}H_{28}NO_4$ m/z = 322.2018, found m/z = 322.2011.

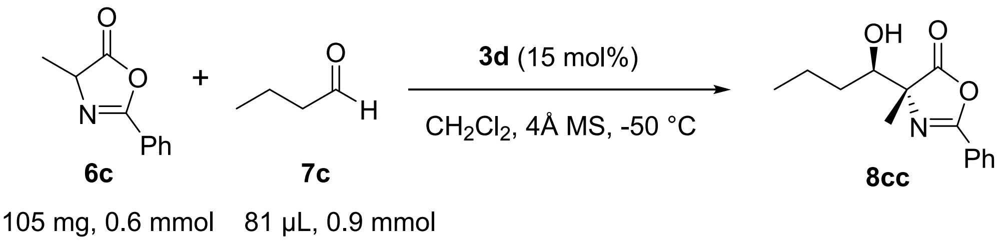

Aldol product **8cc** was made from a 0.6 mmol reaction (4 d) according to the general procedure reported in the previous section. It was used directly for the following steps.

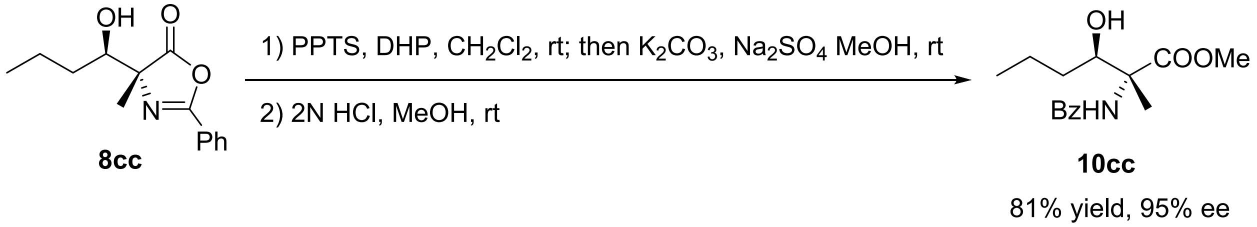

To a 22 mL vial containing Aldol product **8cc** (from 0.6 mmol scale reaction) were added dihydropyran (DHP, 0.82 mL, 9 mmol, 15 eq), CH_2Cl_2 (3 mL) and pyridinium *p*-toluenesulfonate (PPTS, 151 mg, 0.6 mmol, 1 eq) sequentially. The reaction mixture was stirred at room temperature for 9 h. After protection with DHP was complete, anhydrous Na_2SO_4 (600 mg), anhydrous MeOH (3 mL) and K_2CO_3 (207 mg, 1.5 mmol, 2.5 eq) were added to the reaction mixture. After 6.5 h, the reaction mixture was passed through a short silica gel column and concentrated. Then 2N HCl (1 mL) and MeOH (4 mL) was added. After stirring at room temperature for 17 h, the reaction was concentrated and purified by flash column chromatography (1/5 = EA/Hex to 2/5) to give **10cc** as a colorless oil (135 mg, 81%

yield based on a 0.6 mmol Aldol reaction). All reaction times were not optimized. Racemic product was obtained according to the same procedure. Enantiomeric excess of **10cc** was determined by HPLC analysis: Daicel Chiralpak AD-H, Hexanes/IPA = 90/10, 1.0 ml/min, λ = 254 nm, t (*anti*, minor) = 12.87 min, t (*anti*, major) = 13.80 min. $[\alpha]_D^{20}$ = -8.4 (c = 0.45, CH_2Cl_2). **^{1}H NMR** (400 MHz, $CDCl_3$): δ = 7.83 (d, *J* = 7.6 Hz, 2H), 7.64 (s, 1H), 7.56 (t, *J* = 7.3 Hz, 1H), 7.48 (t, *J* = 7.4 Hz, 2H), 5.18 (d, *J* = 10.6 Hz, 1H), 3.98 (t, *J* = 10.1 Hz, 1H), 3.86 (s, 3H), 1.74 (s, 3H), 1.69 – 1.53 (m, 1H), 1.46 – 1.27 (m, 2H), 1.24 – 1.10 (m, 1H), 0.88 (t, *J* = 7.0 Hz, 3H). **^{13}C NMR** (100 MHz, $CDCl_3$): δ = 174.96, 167.95, 133.89, 132.31, 128.94, 127.36, 75.13, 66.15, 53.70, 35.91, 20.80, 19.53, 14.15. **IR** (neat): v = 3394, 2964, 2875, 1731, 1655, 1622, 1575, 1537, 1490, 1463, 1441, 1379, 1325, 1236, 1160, 1115, 1063, 1027, 999, 973, 941, 917, 874, 829, 804, 715, 694, 613 cm^{-1}. **HRMS** (ESI/[M+H]$^+$) = Calcd. for $C_{15}H_{22}NO_4$ m/z = 280.1549, found m/z = 280.1549.

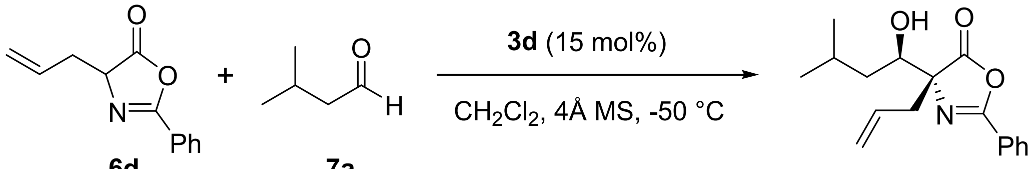

6d 101 mg, 0.5 mmol   **7a** 81 μL, 0.75 mmol   **8da**

Aldol product **8da** was made from a 0.5 mmol reaction (5 d) according to the general procedure reported in the previous section. It was used directly for the following steps.

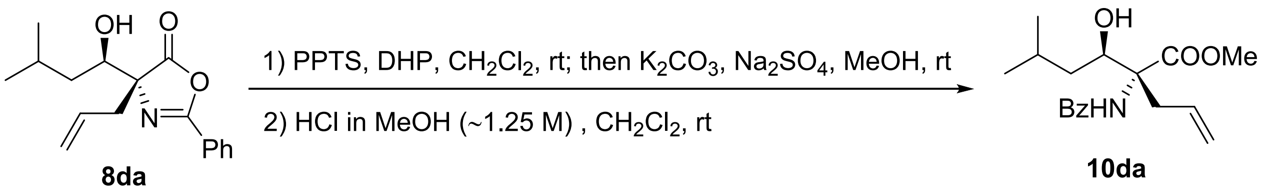

8da   **10da** 90% yield, 95% ee

To a 22 mL vial containing Aldol product **8da** (from 0.5 mmol scale reaction) were added dihydropyran (DHP, 0.68 mL, 7.5 mmol, 15 eq), CH_2Cl_2 (3 mL) and pyridinium *p*-toluenesulfonate (PPTS, 126 mg, 0.5 mmol, 1 eq) sequentially. The reaction mixture was

stirred at room temperature for 17.5 h. After protection with DHP was complete, anhydrous Na_2SO_4 (500 mg), anhydrous MeOH (2.5 mL) and K_2CO_3 (138 mg, 1 mmol, 2 eq) were added to the reaction mixture. After 18 h, the reaction mixture was passed through a short silica gel column and concentrated to remove all volatiles. Then HCl in MeOH (1 mL, ~1.25 M) and CH_2Cl_2 (2 mL) were added. After stirring at room temperature for 6.5 h, the reaction was concentrated and purified by flash column chromatography (1/10 = EA/Hex) to give **10da** as a colorless oil (144 mg, 90% yield based on a 0.5 mmol Aldol reaction). All reaction times were not optimized. Racemic product was obtained according to the same procedure. Enantiomeric excess of **10da** was determined by HPLC analysis: Daicel Chiralpak AD-H, Hexanes/IPA = 90/10, 1.0 ml/min, λ = 254 nm, t (*anti*, minor) = 8.96 min, t (*anti*, major) = 9.76 min. $[\alpha]_D^{20}$ = +28.1 (c = 0.59, CH_2Cl_2). **^{1}H NMR** (400 MHz, $CDCl_3$): δ = 7.83 (d, *J* = 7.2 Hz, 2H), 7.65 – 7.53 (m, 2H), 7.48 (t, *J* = 7.4 Hz, 2H), 5.68 – 5.54 (m, 2H), 5.14 – 5.04 (m, 2H), 4.11 (t, *J* = 10.7 Hz, 1H), 3.85 (s, 3H), 3.40 (dd, *J* = 14.3, 7.8 Hz, 1H), 2.68 (dd, *J* = 14.4, 7.3 Hz, 1H), 2.01 – 1.82 (m, 1H), 1.45 – 1.34 (m, 1H), 0.87 (dd, *J* = 9.7, 6.8 Hz, 6H), 0.81 (d, *J* = 13.2 Hz, 1H). **^{13}C NMR** (100 MHz, $CDCl_3$): δ = 173.44, 168.18, 133.79, 132.33, 132.12, 128.92, 127.34, 119.94, 73.46, 71.02, 53.58, 43.36, 37.06, 24.53, 23.98, 21.55. **IR** (neat): ν = 3396, 2954, 2869, 1731, 1644, 1602, 1579, 1515, 1485, 1439, 1334, 1259, 1224, 1179, 1132, 1089, 1058, 1032, 984, 957, 922, 890, 837, 799, 739, 712, 690, 666, 623 cm^{-1}. **HRMS** (ESI/$[M+H]^+$) = Calcd. for $C_{18}H_{26}NO_4$ m/z = 320.1862, found m/z = 320.1867.

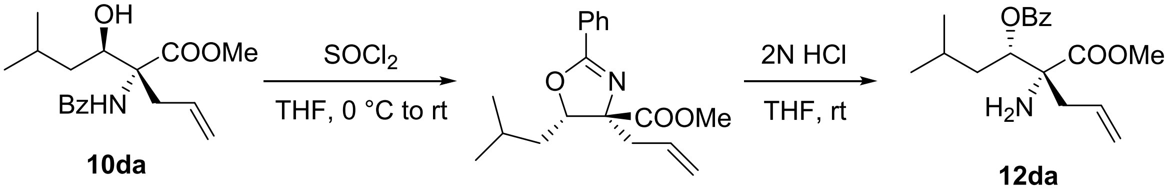

10da *anti*, 95% ee → **11da** → **12da** 85% yield, *syn*

To a 22 mL vial containing product **10da** (52.8 mg, 0.165 mmol) was charged 1.5 mL anhydrous THF, followed by addition of freshly distilled thionyl chloride (120 μL, 1.65 mmol, 10 eq). The reaction was stirred at room temperature for 24 h. Then 1 mL 2N HCl and 0.5 mL THF was added to the reaction mixture. After stirring at room temperature for 22.5 h, saturated $NaHCO_3$ (aq) was added to quench the reaction and render the reaction mixture basic (pH ≈

10), followed by extraction with Et_2O (10 mL x 3). The combined organic layer was washed by brine, dried over $MgSO_4$ and concentrated. The crude was purified by flash column chromatography (1/5 = EA/Hex) to give product **12da** as a pale yellow oil (44.7 mg, 85 % yield over 2 steps). All reaction times were not optimized. $[\alpha]_D^{20}$ = -23.6 (c = 1.00, CH_2Cl_2). **^{1}H NMR** (400 MHz, $CDCl_3$): δ = 8.01 (d, *J* = 7.6 Hz, 2H), 7.57 (t, *J* = 7.4 Hz, 1H), 7.44 (t, *J* = 7.6 Hz, 2H), 5.64 (td, *J* = 16.7, 9.0 Hz, 1H), 5.54 (d, *J* = 10.5 Hz, 1H), 5.24 – 5.13 (m, 2H), 3.65 (s, 3H), 2.65 (dd, *J* = 13.3, 6.2 Hz, 1H), 2.28 (dd, *J* = 13.3, 8.5 Hz, 1H), 1.90 – 1.79 (m, 1H), 1.73 (s, 2H), 1.66 – 1.52 (m, 1H), 1.50 – 1.38 (m, 1H), 0.99 (d, *J* = 6.4 Hz, 3H), 0.92 (d, *J* = 6.6 Hz, 3H). **^{13}C NMR** (100 MHz, $CDCl_3$): δ = 175.62, 165.66, 133.17, 131.86, 129.94, 129.77, 128.52, 120.50, 76.59, 64.39, 52.49, 40.20, 38.34, 24.51, 24.02, 21.68. **IR** (neat): ν = 3074, 2956, 2929, 2871, 2366, 2343, 1728, 1720, 1602, 1451, 1269, 1217, 1177, 1109, 1070, 1026, 997, 925, 855, 815, 712, 667 cm^{-1}. **HRMS** (ESI/$[M+H]^+$) = Calcd. for $C_{18}H_{26}NO_4$ m/z = 320.1862, found m/z = 320.1856.

Determination of enantiomeric excess of product **11da**:

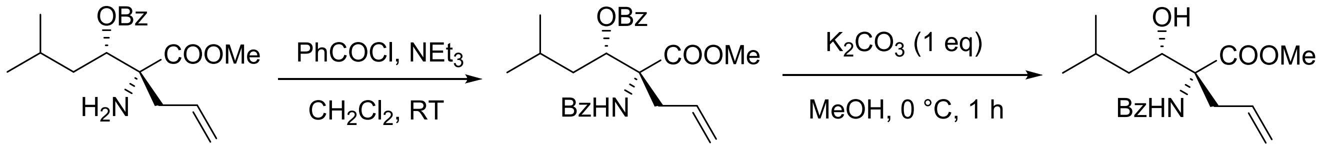

12da **13da** **14da**, 95% ee

To a solution of **12da** (23.2 mg, 0.073 mmol) and benzoyl chloride (12.7 µL, 1.5 eq) in CH_2Cl_2 (1 mL) was added triethylamine (30 µL, 3 eq). The reaction was stirred at room temperature for 17 h. Then 1N HCl was added to quench the reaction, followed by extraction with Et_2O (10 mL x 3). The combine organic layer was washed with sat. $NaHCO_3$ and brine. Then it was dried over $MgSO_4$ and concentrated. The crude was purified by flash column chromatography (1/10 = EA/Hex) to give **13da** as a colorless oil (27.3 mg, 89% yield) which contained small amount of impurities. Half of this compound (13.5 mg) was dissolved in MeOH (1 mL) and cooled in an ice-bath with stirring. Then K_2CO_3 (6.6 mg) was added in one portion to the reaction. After stirring at 0 C for 1 h, the reaction was diluted by water and extracted with Et_2O (5 mL x 3). The combined organic layer was washed with brine, dried over $MgSO_4$ and concentrated. The

crude was purified by preparative thin-layer chromatography to give **14da** as a pale white solid (8.4 mg, 83% yield, 95% ee). Enantiomeric excess of **14da** was determined by HPLC analysis: Daicel Chiralpak AD-H, Hexanes/IPA = 90/10, 1.0 ml/min, λ = 254 nm, t (*anti*, minor) = 7.79 min, t (*anti*, major) = 12.99 min. $[\alpha]_D^{20}$ = -148.1 (c = 0.15, CH_2Cl_2). **^{1}H NMR** (400 MHz, $CDCl_3$): δ = 7.78 (d, *J* = 7.7 Hz, 2H), 7.54 (t, *J* = 7.2 Hz, 1H), 7.46 (t, *J* = 7.4 Hz, 2H), 6.71 (s, 1H), 5.69 (dt, *J* = 15.3, 9.5 Hz, 1H), 5.39 – 5.23 (m, 2H), 4.76 (d, *J* = 11.0 Hz, 1H), 4.13 (t, *J* = 10.6 Hz, 1H), 3.83 (s, 3H), 2.95 (dd, *J* = 13.7, 4.8 Hz, 1H), 2.71 (dd, *J* = 13.7, 9.8 Hz, 1H), 2.03 – 1.87 (m, 1H), 1.44 – 1.32 (m, 1H), 1.32 – 1.20 (m, 2H), 0.93 (t, *J* = 7.0 Hz, 6H). **^{13}C NMR** (100 MHz, $CDCl_3$): δ = 171.96, 168.23, 134.41, 132.48, 132.05, 128.83, 127.15, 121.01, 73.38, 69.01, 53.02, 41.62, 38.84, 24.72, 24.09, 21.59. **IR** (neat): ν = 3460, 3389, 2954, 2865, 1730, 1637, 1603, 1579, 1517, 1481, 1435, 1413, 1330, 1292, 1258, 1231, 1176, 1137, 1092, 1055, 1030, 988, 960, 919, 887, 871, 846, 803, 773, 707, 687, 626 cm^{-1}. **HRMS** (ESI/$[M+H]^+$) = Calcd. for $C_{18}H_{26}NO_4$ m/z = 320.1862, found m/z = 320.1862.

5. Determination of relative configurations

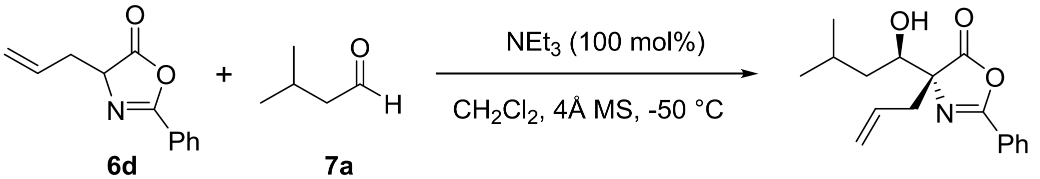

6d 0.53 mmol

7a 0.8 mmol, 1.5 eq

rac-**8da**

Aldol product *rac*-**8da** was made from a 0.53 mmol reaction (4 d) according to the general procedure reported in the previous section. It was used directly for the following steps.

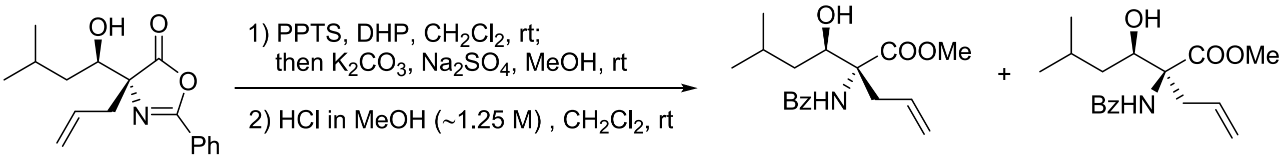

rac-**8da**

anti, *rac*-**10da**

syn, *rac*-**14da**

The reation was carried out by following the same procedure used for compound **10da**, giving *rac*-**10da** as a colorless oil (90.6 mg, 53% yield) and *rac*-**14da** as a pale yellow solid (64.5 mg, 38% yield). All spectroscopic data were the same as those of **10da** and **14da**.

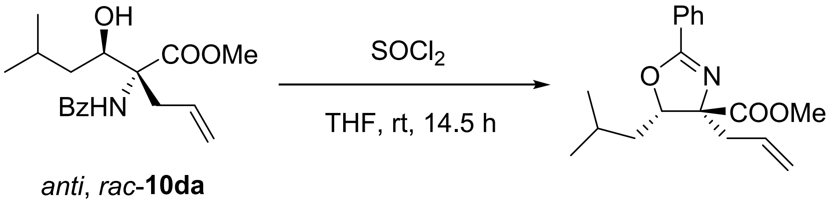

To a solution of rac-**10da** (30.7 mg, 0.096 mmol) in 1 mL anhydrous THF was added freshly distilled thionyl chloride (35 μL, 0.48 mmol, 5 eq) at room temperature. The reaction was stirred at room temperature for 14.5 h. Then saturated $NaHCO_3$ (aq) was added to quench the reaction and render the reaction mixture basic (pH ≈ 10), followed by extraction with Et_2O (10 mL x 3). The combined organic layer was washed by brine, dried over $MgSO_4$ and concentrated. The crude was purified by flash column chromatography (1/5 = EA/Hex) to give product *rac*-**11da** as a brown oil (25.9 mg, 89% yield). **^{1}H NMR** (400 MHz, $CDCl_3$): δ = 8.02 – 7.97 (m, 2H), 7.52 – 7.45 (m, 1H), 7.44 – 7.36 (m, 2H), 5.97 – 5.81 (m, 1H), 5.18 – 5.08 (m, 2H), 4.82 (dd, *J* = 10.9, 2.6 Hz, 1H), 3.78 (s, 3H), 2.65 (dd, *J* = 13.9, 7.6 Hz, 1H), 2.52 (dd, *J* = 13.9, 6.5 Hz, 1H), 2.03 – 1.87 (m, 1H), 1.78 (ddd, *J* = 14.1, 10.9, 5.2 Hz, 1H), 1.61 (ddd, *J* = 14.0, 8.5, 2.7 Hz, 1H), 1.04 (dd, *J* = 6.6, 4.2 Hz, 6H). **^{13}C NMR** (100 MHz, $CDCl_3$): δ = 173.80, 164.23, 132.96, 131.80, 128.67, 128.35, 127.59, 118.85, 83.68, 79.08, 52.65, 38.55, 38.31, 26.08, 23.59, 21.89. **IR** (neat): v = 3073, 2956, 2877, 2341, 1963, 1911, 1730, 1643, 1580, 1495, 1450, 1444, 1354, 1322, 1250, 1228, 1136, 1086, 1069, 1026, 975, 919, 836, 780, 697 cm^{-1}. **HRMS** (ESI/$[M+H]^+$) = Calcd. for $C_{18}H_{26}NO_4$ m/z = 302.1756, found m/z = 302.1763.

Relative configuration of *rac*-**11da** was determined by 1D NOESY experiment. Therefore, relative configuration of *rac*-**10da** was determined to be *anti*. Relative configurations of all the other products were assigned based on these results.

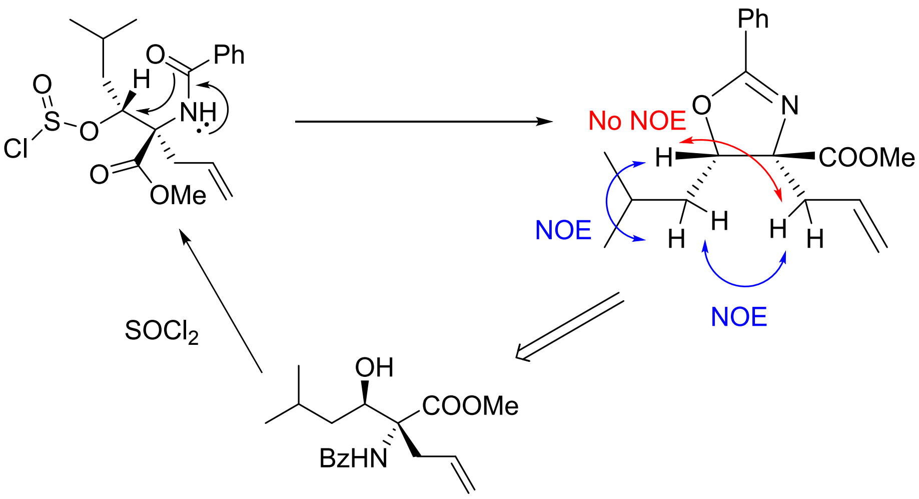

10da

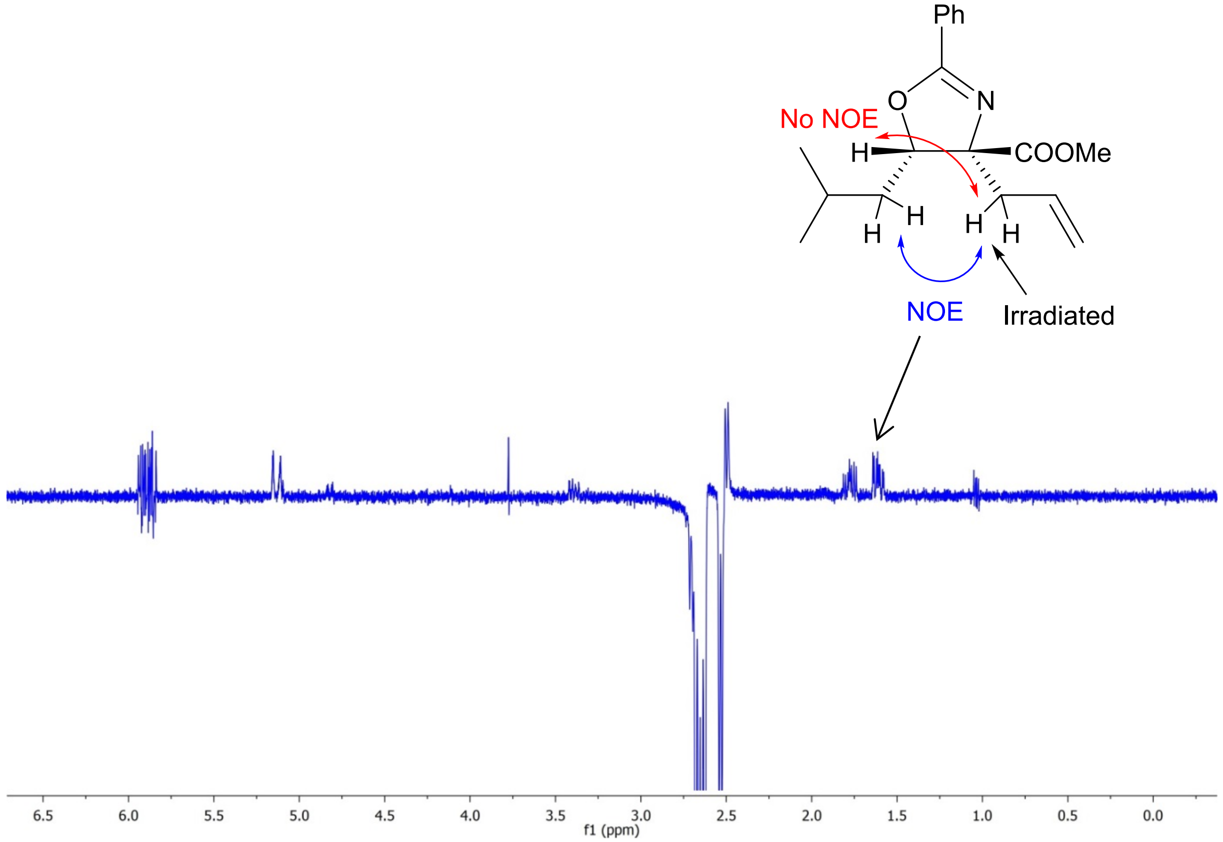

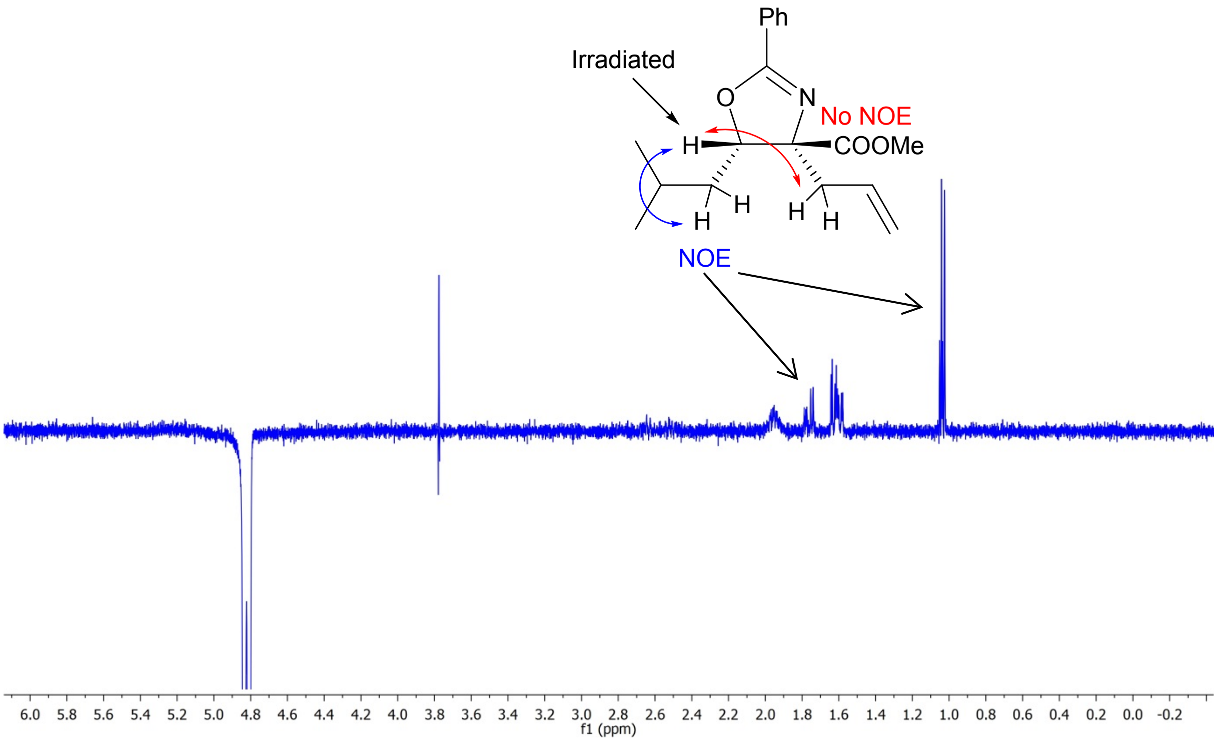

6. Determination of absolute configurations

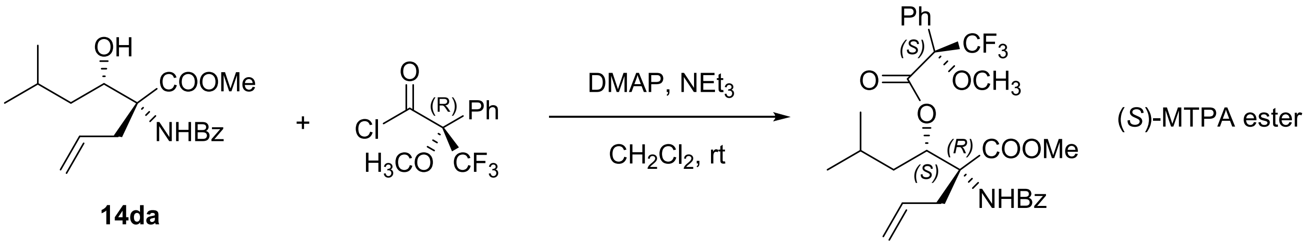

To a solution of **14da** (4.2 mg, 0.013 mmol) in CH_2Cl_2 (0.5 mL) were added (*R*)-(−)-α-Methoxy-α-(trifluoromethyl)phenylacetyl chloride ((*R*)-(−)-MTPA-Cl, Mosher's acid chloride, 4 mg, 0.0156 mmol, 1.2 equiv), triethylamine (18 μL, 0.13 mmol, 10 equiv) and 4-dimethylaminopyridine (DMAP, 3.2 mg, 0.026 mmol, 2 equiv) sequentially at room temperature. After stirring for 14 h, the reaction mixture was concentrated and purified by preparative thin-

layer chromatography (1/5 = EA/Hex) to give *(S)*-**15da** as a colorless oil (4.6 mg, 66% yield). **^{1}H NMR** (400 MHz, $CDCl_3$): δ = 7.72 (d, *J* = 7.7 Hz, 2H), 7.58 (d, *J* = 7.1 Hz, 2H), 7.52 (t, *J* = 7.3 Hz, 1H), 7.48 – 7.33 (m, 5H), 7.03 (s, 1H), 6.09 (d, *J* = 8.9 Hz, 1H), 5.57 (td, *J* = 17.3, 7.8 Hz, 1H), 5.10 – 4.95 (m, 2H), 3.77 (s, 3H), 3.54 (s, 3H), 3.47 (dd, *J* = 13.8, 7.0 Hz, 1H), 2.55 (dd, *J* = 13.7, 7.6 Hz, 1H), 1.66 – 1.53 (m, 2H), 1.53 – 1.45 (m, 1H), 0.90 (d, *J* = 6.3 Hz, 3H), 0.85 (d, *J* = 6.5 Hz, 3H). **HRMS** (ESI/$[M+H]^+$) = Calcd. for $C_{18}H_{26}NO_4$ m/z = 536.2260, found m/z = 536.2265.

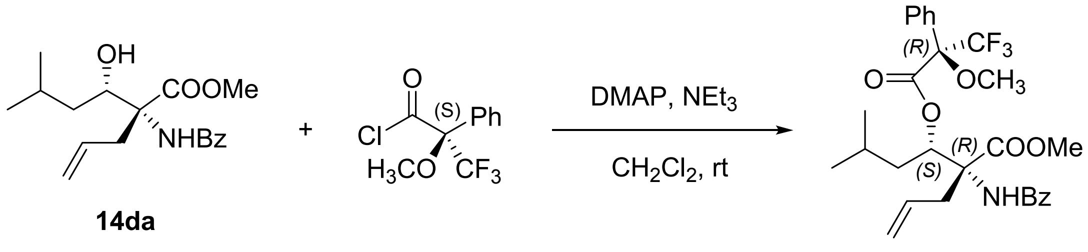

14da

(*R*)-**15da**

(*R*)-MTPA ester

By following the same procedure, product *(R)*-**15da** was synthesized from (*S*)-(−)-MTPA-Cl and obtained as a colorless oil (4.5 mg, 65% yield). **^{1}H NMR** (400 MHz, $CDCl_3$): δ = 7.72 (d, *J* = 7.7 Hz, 2H), 7.58 (d, *J* = 7.1 Hz, 2H), 7.52 (t, *J* = 7.3 Hz, 1H), 7.48 – 7.33 (m, 5H), 7.03 (s, 1H), 6.09 (d, *J* = 8.9 Hz, 1H), 5.57 (td, *J* = 17.3, 7.8 Hz, 1H), 5.10 – 4.95 (m, 2H), 3.77 (s, 3H), 3.54 (s, 3H), 3.47 (dd, *J* = 13.8, 7.0 Hz, 1H), 2.55 (dd, *J* = 13.7, 7.6 Hz, 1H), 1.66 – 1.53 (m, 2H), 1.53 – 1.45 (m, 1H), 0.90 (d, *J* = 6.3 Hz, 3H), 0.85 (d, *J* = 6.5 Hz, 3H). **HRMS** (ESI/$[M+H]^+$) = Calcd. for $C_{18}H_{26}NO_4$ m/z = 536.2260, found m/z = 536.2267.

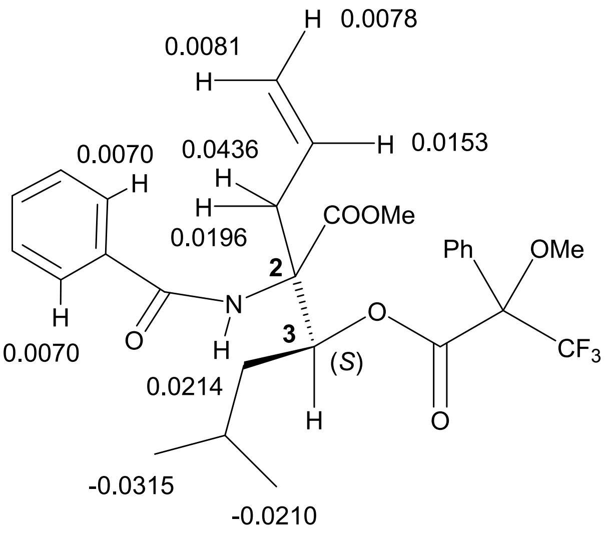

MPTA ester **15da**

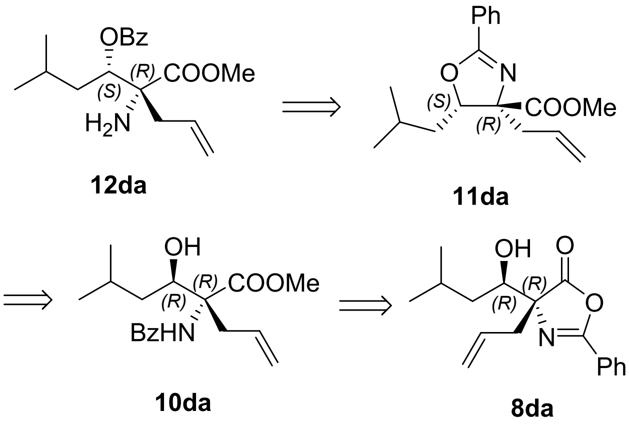

By employing the modified Mosher's method[15-17], the absolute configuration at the C3 position of **15da** was determined to be *S* on the basis of the Δδ values ($\delta_{(S)\text{-MTPA}}$ - $\delta_{(R)\text{-MTPA}}$ in ppm). Based on the relative stereochemistry, the absolute configuration at the C2 position of **15da** was determined to be *R*. Therefore, the absolute configurations of **12da**, **11da**, **10da** and **8da** were all assigned. Absolute configurations of all the other products were assigned based on these results.

7. Reference:

1) Weber, M.; Frey, W.; Peters, R. *Angew. Chem. Int. Ed.* **2013**, *52*, 13223-13227.
2) Zhu, R.; Buchwald, S. L. *J. Am. Chem. Soc.* **2012**, *134*, 12462-12465.
3) Metrano, A. J., Miller, S. J. *J. Org. Chem.* **2014**, *79*, 1542-1554.
4) Witkowska, R.; Kaczmarek, K.; Crisma, M.; Toniolo, C.; Zabrocki *J. Peptide Sci.* **2001**, *7*, 619-625.
5) Yang, C.-T.; Chandrasekharan, P.; He, T.; Poh, Z.; Raju, A.; Chuang, K.-H.; Robins, E. G. *Biomaterials* **2014**, *35*, 327-336.
6) Chen, F. M. F.; Kuroda, K.; Benoiton, N. L. *Synthesis* **1979**, 230-232.
7) Melhado, A. D.; Luparia, M.; Toste, F. D. *J. Am. Chem. Soc.* **2007**, *129*, 12638-12639.
8) Finkbeiner, P; Weckenmann, N. M.; Nachtsheim, B. J. *Org. Lett*. **2014**, *16*, 1326-1329.

9) Li, H.; Wang, Y.; Tang, L.; Deng, L. *J. Am. Chem. Soc.* **2004**, *126*, 9906-9907.
10) Wu, F.; Hong, R.; Khan, J.; Liu, X.; Deng, L. *Angew. Chem. Int. Ed.* **2006**, *45*, 4301-4305.
11) Li, H.; Wang, B. *J. Am. Chem. Soc.* **2006**, *128*, 732-733.
12) Wang, Y.; Li, H.; Wang, Y.-Q.; Liu, Y.; Foxman, B. M. *J. Am. Chem. Soc.* **2007**, *129*, 6364-6365.
13) Bartelson, K. J.; Singh, R. P.; Foxman, B. M.; Deng, L. *Chem. Sci.* **2011**, *2*, 1940-1944.
14) Vakulya, B.; Varga, S.; Csámpai, A.; Soós, T. *Org. Lett.* **2005**, *7*, 1967-1969.
15) Ohtani, I.; Kusumi, T.; Kashman, Y.; Kakisawa, H. *J. Am. Chem. Soc.* **1991**, *113*, 4092-4096.
16) Fujita, T.; Hamamichi, N.; Kiuchi, M.; Matsuzaki, T.; Kitao, Y.; Inoue, K.; Hirose, R.; Yoneta, M.; Sasaki, S.; Chiba, K. *J. Antibiot.* **1996**, *49*, 846-53.
17) Terada, M.; Tanaka, H.; Sorimachi, K. *J. Am. Chem. Soc.* **2009**, *131*, 3430-3431.

8. ^{1}H and ^{13}C NMR Spectra for New Compounds, HPLC Spectra for chiral products

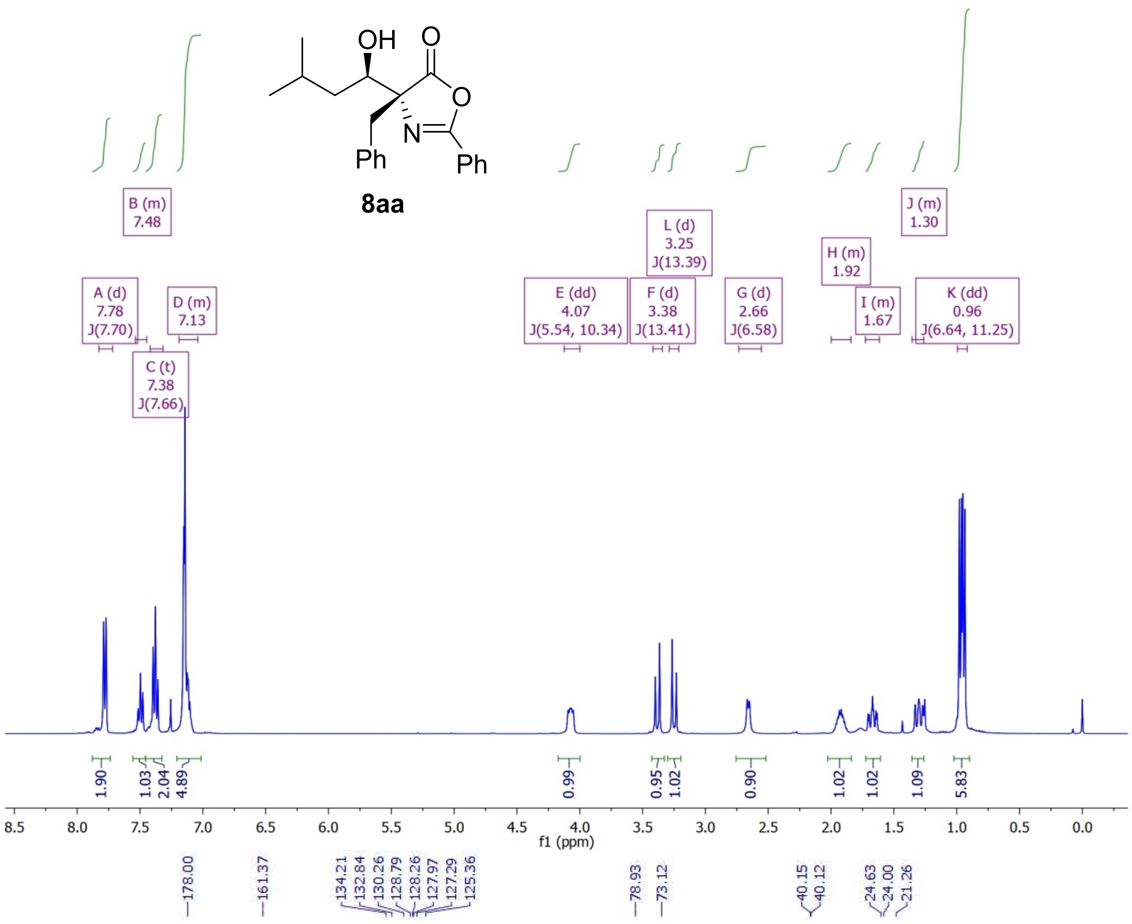

OH
O
N
Ph
Ph
8aa
A (d) 7.78 J(7.70)
B (m) 7.48
C (t) 7.38 J(7.66)
D (m) 7.13
E (dd) 4.07 J(5.54, 10.34)
F (d) 3.38 J(13.41)
L (d) 3.25 J(13.39)
G (d) 2.66 J(6.58)
H (m) 1.92
I (m) 1.67
J (m) 1.30
K (dd) 0.96 J(6.64, 11.25)
1.90
1.03
2.04
4.89
0.99
0.95
1.02
0.90
1.02
1.02
1.09
5.83
f1 (ppm)
178.00
161.37
134.21
132.84
130.26
128.79
128.26
127.97
127.29
125.36
78.93
73.12
40.15
40.12
24.63
24.00
21.26

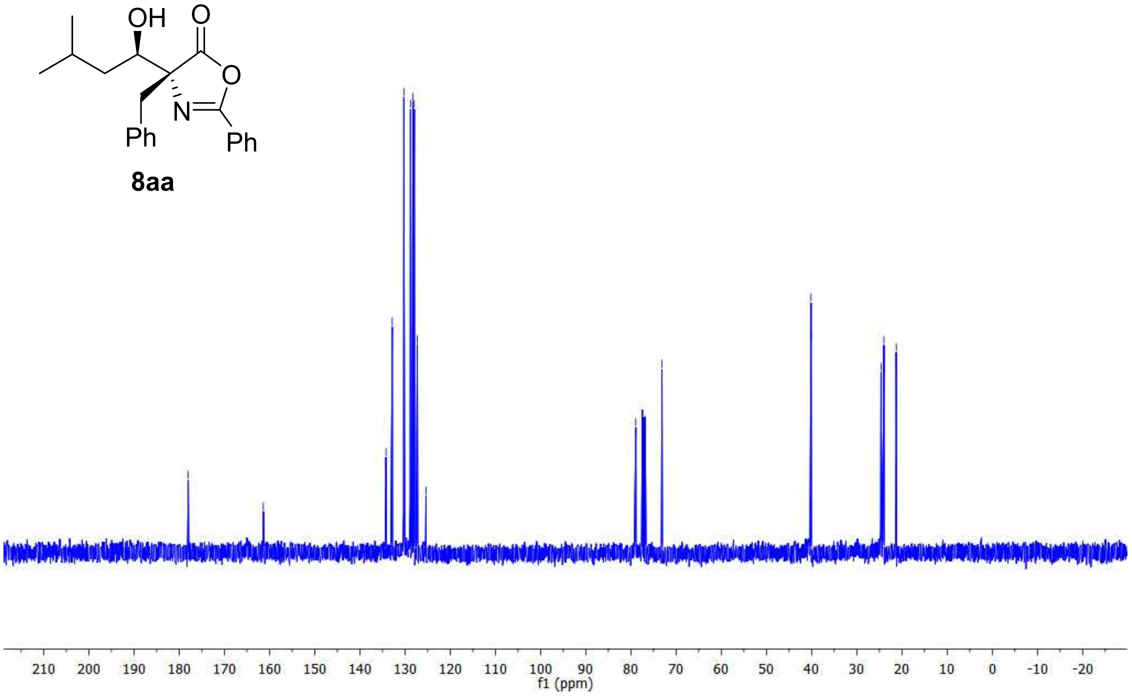

OH
O
N
Ph
Ph
8aa
f1 (ppm)

Data File C:\CHEM32\1\DATA\YZHENG\3-63RAC_1.D
Sample Name: 3-63rac

===

```
Acq. Operator   : yang
Acq. Instrument : Instrument 1                    Location :   -
Injection Date  : 8/1/2011 2:32:52 PM
Acq. Method     : C:\CHEM32\1\METHODS\METHOD-1_LC.M
Last changed    : 8/1/2011 2:00:23 PM by yang
                  (modified after loading)
Analysis Method : C:\CHEM32\1\METHODS\METHOD-1_LC.M
Last changed    : 8/1/2011 5:37:18 PM by yang
                  (modified after loading)
Sample Info     : AS-H, Hex:IPA = 95:5, 1.0 mL/min, 254 nm, 44 bar, Right
```

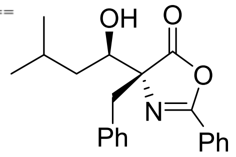

8aa

Racemic

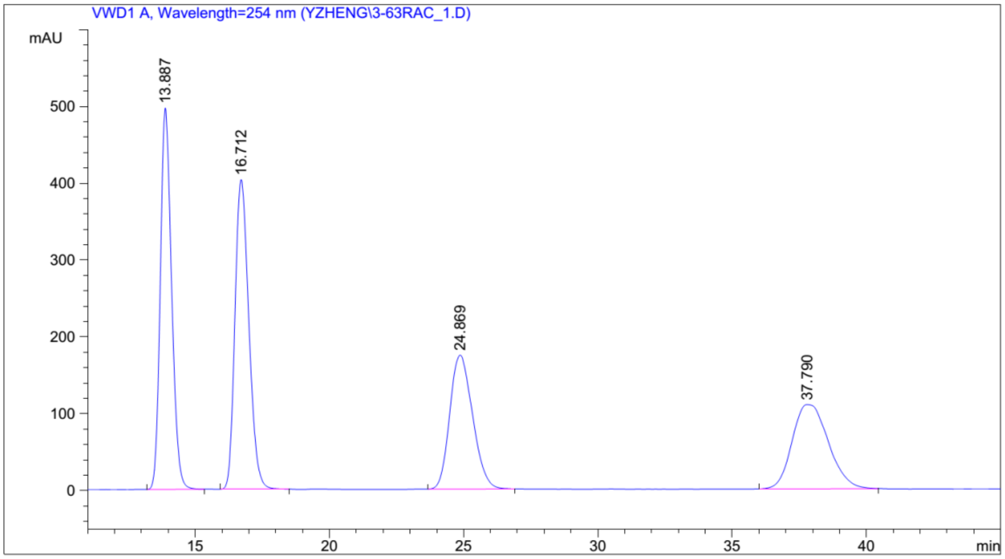

===

Area Percent Report

===

```
Sorted By             :      Signal
Multiplier            :      1.0000
Dilution              :      1.0000
Use Multiplier & Dilution Factor with ISTDs
```

Signal 1: VWD1 A, Wavelength=254 nm

Peak #	RetTime [min]	Type	Width [min]	Area mAU *s	Height [mAU]	Area %
1	13.887	BB	0.4461	1.44565e4	496.35040	29.2101
2	16.712	BB	0.5614	1.44665e4	403.04199	29.2304
3	24.869	BB	0.9422	1.02947e4	174.34625	20.8009
4	37.790	BB	1.4103	1.02737e4	109.66785	20.7586
Totals :				4.94914e4	1183.40650	

===

*** End of Report ***

Instrument 1 8/1/2011 5:37:22 PM yang   Page   1 of 1

Data File C:\CHEM32\1\DATA\YZHENG\3-62.D
Sample Name: 3-62

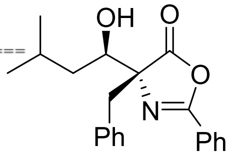

8aa

94% ee, *anti*/*syn* = 97.5/2.5

===

Acq. Operator : yang
Acq. Instrument : Instrument 1 Location : -
Injection Date : 8/1/2011 4:17:13 PM
Acq. Method : C:\CHEM32\1\METHODS\METHOD-1_LC.M
Last changed : 8/1/2011 3:55:52 PM by yang
(modified after loading)
Analysis Method : C:\CHEM32\1\METHODS\WASH.M
Last changed : 8/10/2011 11:12:50 AM by jhl
(modified after loading)
Sample Info : AS-H, Hex:IPA = 95:5, 1.0 mL/min, 254 nm, 44 bar, Right

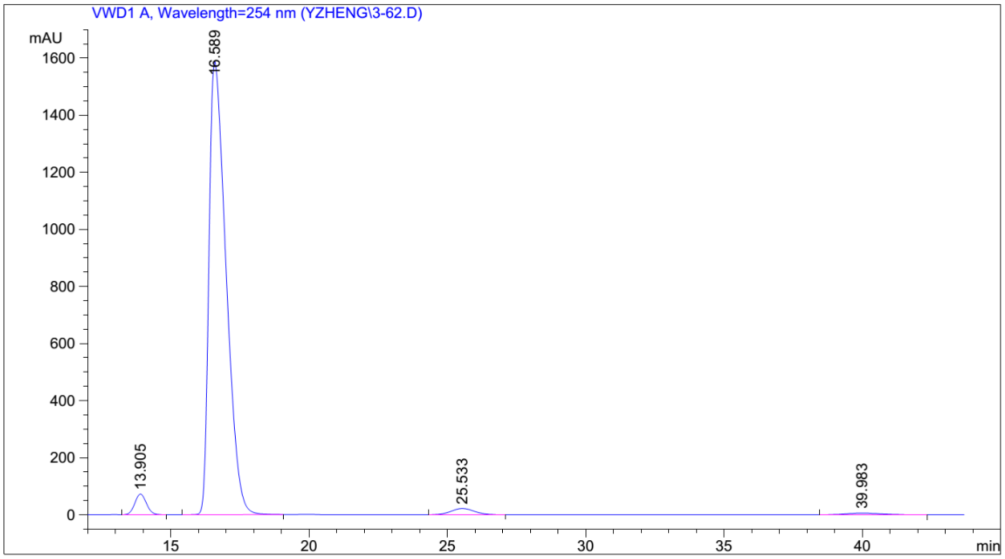

===

Area Percent Report

===

Sorted By : Signal
Multiplier : 1.0000
Dilution : 1.0000
Use Multiplier & Dilution Factor with ISTDs

Signal 1: VWD1 A, Wavelength=254 nm

Peak #	RetTime [min]	Type	Width [min]	Area mAU *s	Height [mAU]	Area %
1	13.905	VB	0.4595	2129.86011	72.44035	2.9648
2	16.589	BB	0.6545	6.78960e4	1588.21118	94.5130
3	25.533	BB	0.8975	1288.76758	21.95257	1.7940
4	39.983	BB	1.1716	523.13538	5.65183	0.7282

Totals : 7.18378e4 1688.25594

===

*** End of Report ***

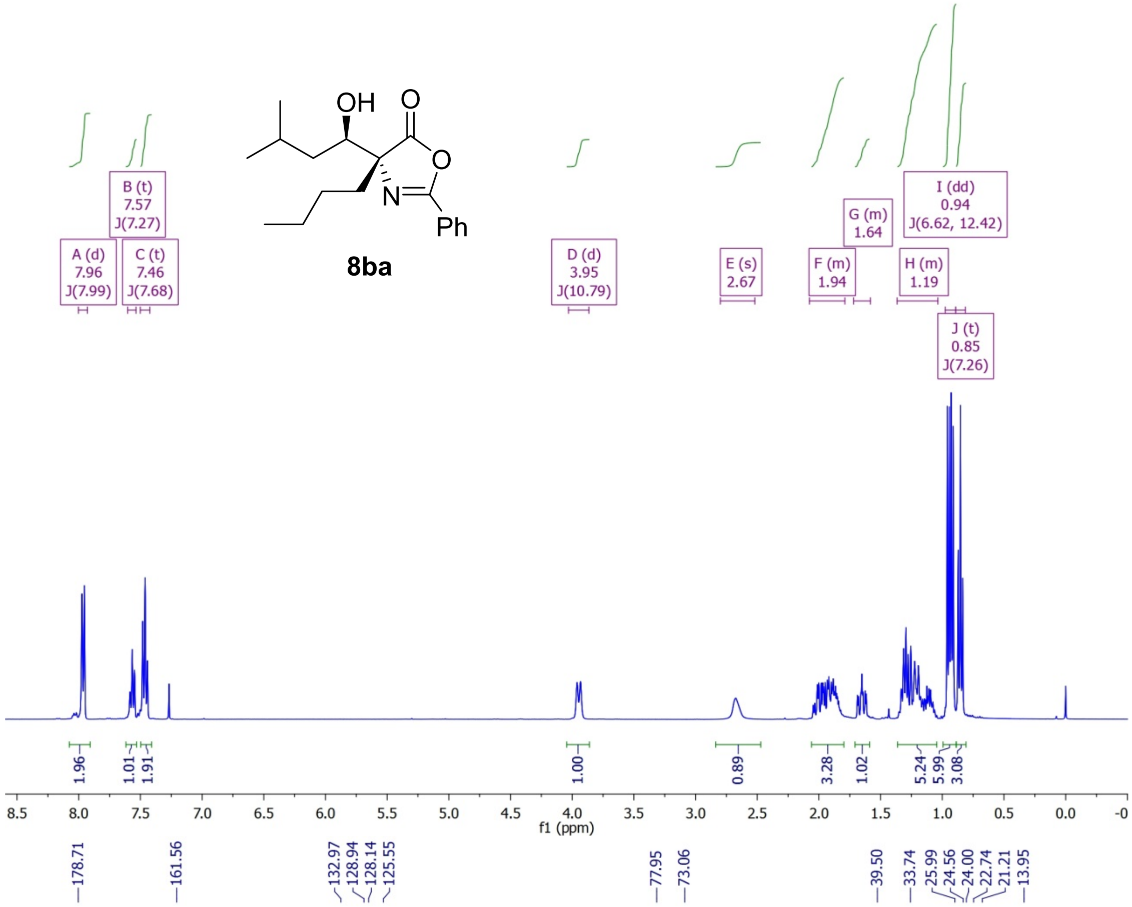
OH O
O
N
Ph
8ba
A (d) 7.96 J(7.99)
B (t) 7.57 J(7.27)
C (t) 7.46 J(7.68)
D (d) 3.95 J(10.79)
E (s) 2.67
F (m) 1.94
G (m) 1.64
H (m) 1.19
I (dd) 0.94 J(6.62, 12.42)
J (t) 0.85 J(7.26)
1.96
1.01
1.91
1.00
0.89
3.28
1.02
5.24
5.99
3.08
8.5 8.0 7.5 7.0 6.5 6.0 5.5 5.0 4.5 4.0 3.5 3.0 2.5 2.0 1.5 1.0 0.5 0.0 -0
f1 (ppm)
178.71
161.56
132.97
128.94
128.14
125.55
77.95
73.06
39.50
33.74
25.99
24.56
24.00
22.74
21.21
13.95

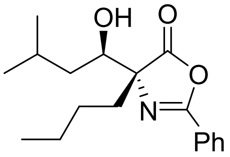
OH O
O
N
Ph

8ba

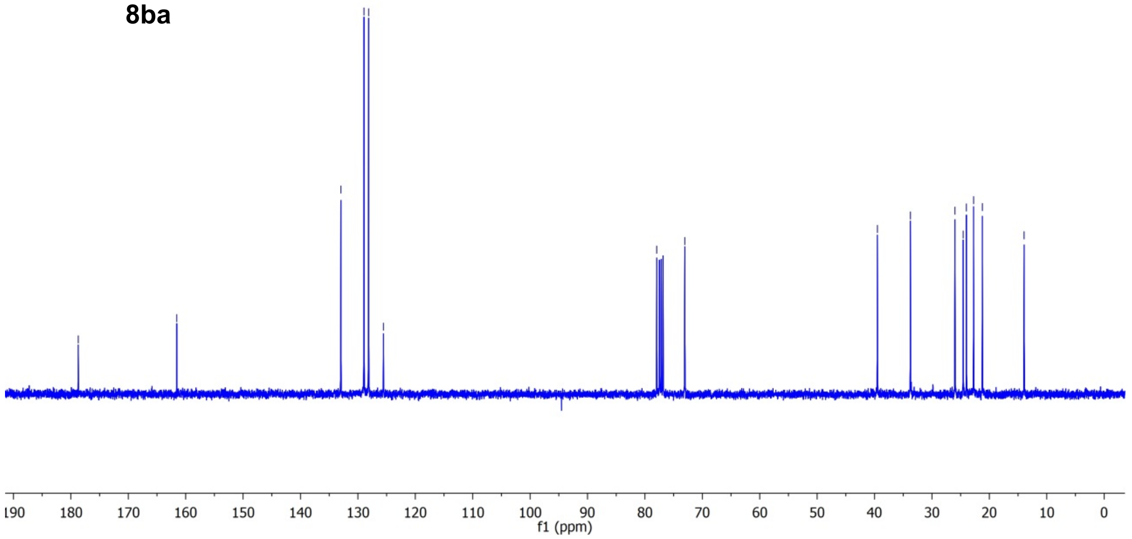
190 180 170 160 150 140 130 120 110 100 90 80 70 60 50 40 30 20 10 0
f1 (ppm)

Data File C:\CHEM32\1\DATA\YZHENG\3-63RAC_1.D
Sample Name: 3-63rac

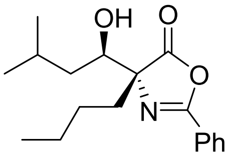

8ba

Racemic

===

Acq. Operator : yang
Acq. Instrument : Instrument 1 Location : -
Injection Date : 8/1/2011 2:32:52 PM
Acq. Method : C:\CHEM32\1\METHODS\METHOD-1_LC.M
Last changed : 8/1/2011 2:00:23 PM by yang
(modified after loading)
Analysis Method : C:\CHEM32\1\METHODS\METHOD-1_LC.M
Last changed : 8/1/2011 5:37:18 PM by yang
(modified after loading)
Sample Info : AS-H, Hex:IPA = 95:5, 1.0 mL/min, 254 nm, 44 bar, Right

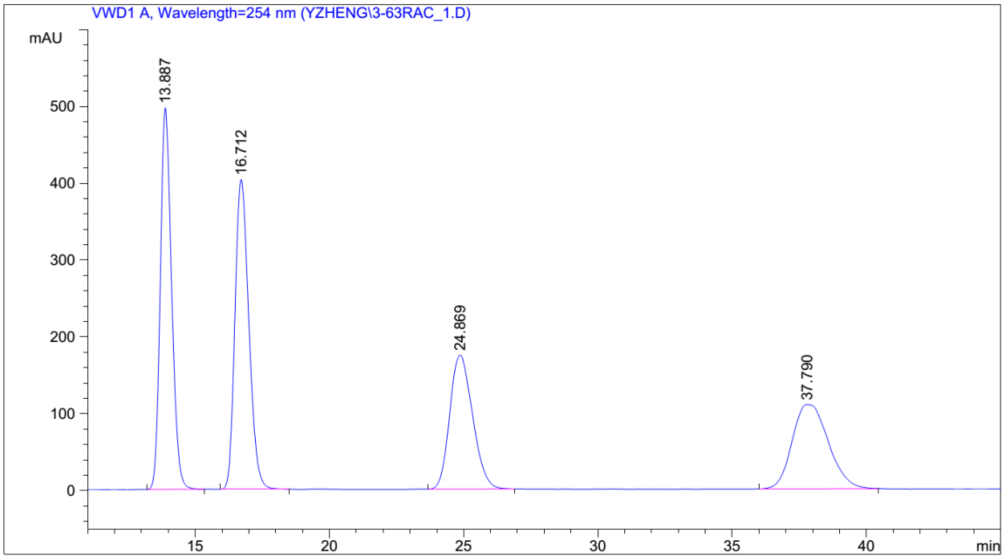

===

Area Percent Report

===

Sorted By : Signal
Multiplier : 1.0000
Dilution : 1.0000
Use Multiplier & Dilution Factor with ISTDs

Signal 1: VWD1 A, Wavelength=254 nm

Peak #	RetTime [min]	Type	Width [min]	Area mAU *s	Height [mAU]	Area %
1	13.887	BB	0.4461	1.44565e4	496.35040	29.2101
2	16.712	BB	0.5614	1.44665e4	403.04199	29.2304
3	24.869	BB	0.9422	1.02947e4	174.34625	20.8009
4	37.790	BB	1.4103	1.02737e4	109.66785	20.7586
Totals :				4.94914e4	1183.40650	

===

*** End of Report ***

Data File C:\CHEM32\1\DATA\YZHENG\3-62.D
Sample Name: 3-62

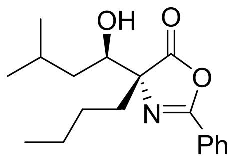

8ba

95% ee, *anti*/*syn* = 98/2

===

Acq. Operator : yang
Acq. Instrument : Instrument 1 Location : -
Injection Date : 8/1/2011 4:17:13 PM
Acq. Method : C:\CHEM32\1\METHODS\METHOD-1_LC.M
Last changed : 8/1/2011 3:55:52 PM by yang
(modified after loading)
Analysis Method : C:\CHEM32\1\METHODS\WASH.M
Last changed : 8/10/2011 11:12:50 AM by jhl
(modified after loading)
Sample Info : AS-H, Hex:IPA = 95:5, 1.0 mL/min, 254 nm, 44 bar, Right

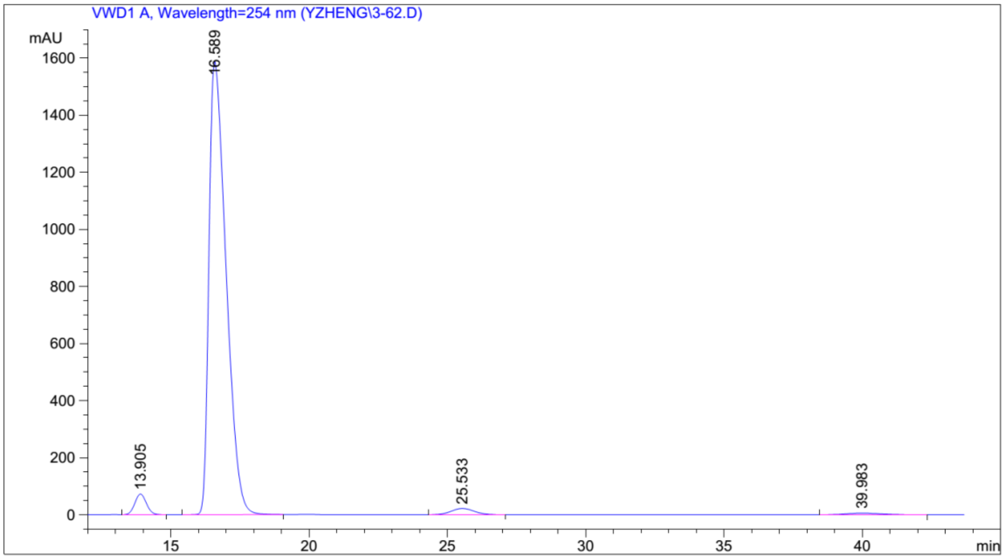

===

Area Percent Report

===

Sorted By : Signal
Multiplier : 1.0000
Dilution : 1.0000
Use Multiplier & Dilution Factor with ISTDs

Signal 1: VWD1 A, Wavelength=254 nm

Peak #	RetTime [min]	Type	Width [min]	Area mAU *s	Height [mAU]	Area %
1	13.905	VB	0.4595	2129.86011	72.44035	2.9648
2	16.589	BB	0.6545	6.78960e4	1588.21118	94.5130
3	25.533	BB	0.8975	1288.76758	21.95257	1.7940
4	39.983	BB	1.1716	523.13538	5.65183	0.7282

Totals : 7.18378e4 1688.25594

===

*** End of Report ***

Data File C:\CHEM32\1\DATA\YZHENG\3-91.D
Sample Name: 3-91

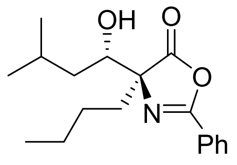

ent-**8ba**

-90% ee, *anti*/*syn* = 97.5/2.5

===

```
Acq. Operator   : yang
Acq. Instrument : Instrument 1                    Location : Vial 51
Injection Date  : 9/12/2011 5:33:19 PM
                                               Inj Volume : 5 µl
Acq. Method     : C:\CHEM32\1\METHODS\YANG.M
Last changed    : 9/12/2011 5:13:01 PM by yang
                  (modified after loading)
Analysis Method : C:\CHEM32\1\METHODS\METHOD1.M
Last changed    : 9/12/2011 6:07:44 PM by xiao
                  (modified after loading)
Sample Info     : AD-H+AD, Hex:IPA = 95:5, 0.8 mL/min, 254 nm, 47 bar, Le
                  ft,
```

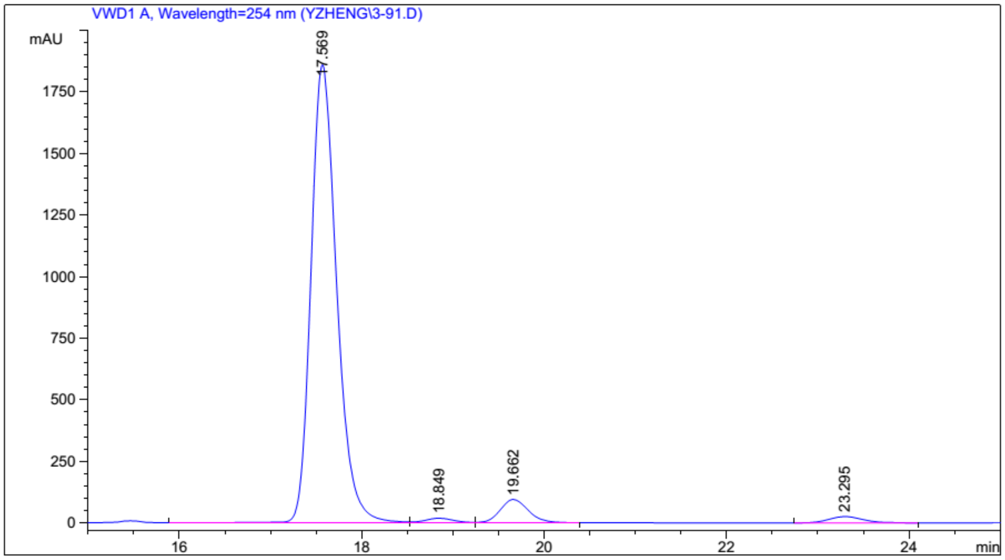

===
Area Percent Report
===

```
Sorted By             :      Signal
Multiplier            :      1.0000
Dilution              :      1.0000
Sample Amount         :      1.00000  [ng/ul]   (not used in calc.)
Use Multiplier & Dilution Factor with ISTDs
```

Signal 1: VWD1 A, Wavelength=254 nm

Peak #	RetTime [min]	Type	Width [min]	Area mAU *s	Height [mAU]	Area %
1	17.569	VV	0.3010	3.63684e4	1859.21899	92.2223
2	18.849	VV	0.3221	391.98923	18.67219	0.9940
3	19.662	VB	0.3268	2006.05396	94.84164	5.0869
4	23.295	BB	0.4001	669.13727	25.92393	1.6968
Totals :				3.94355e4	1998.65674	

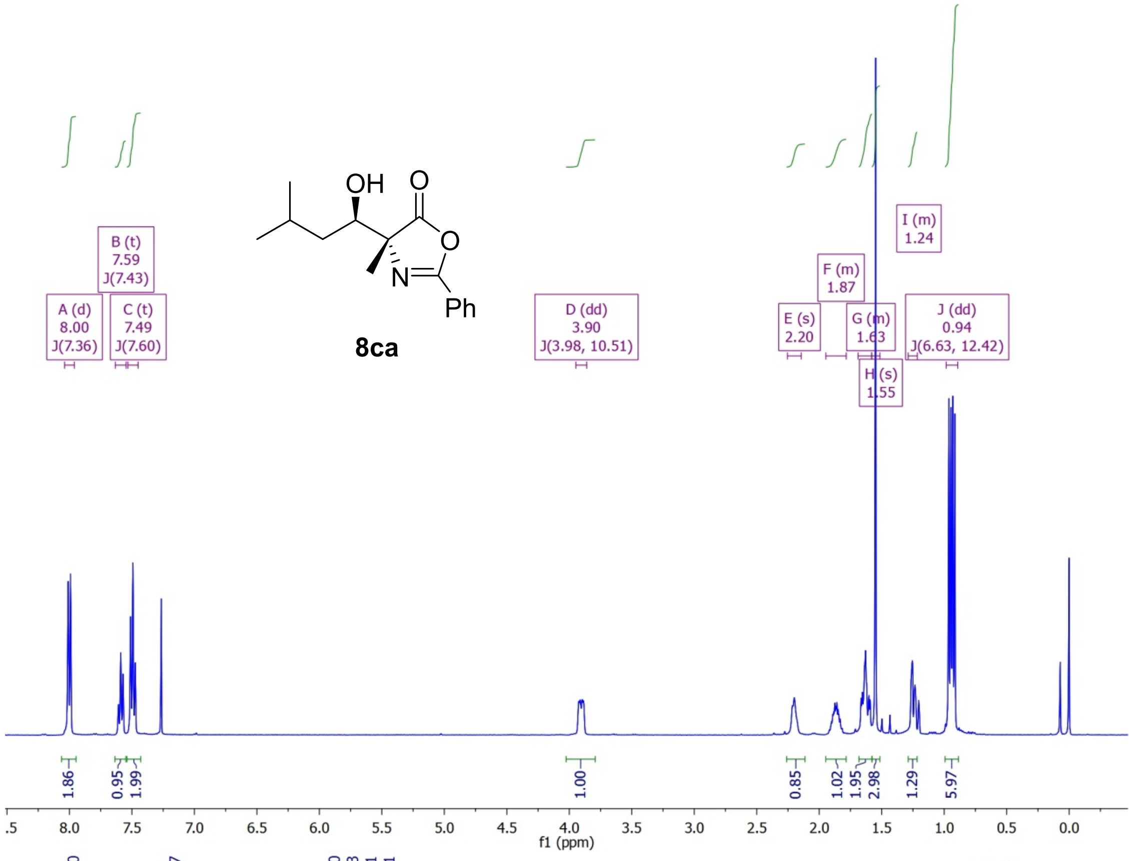
OH O
O
N
Ph
8ca
A (d) 8.00 J(7.36)
B (t) 7.59 J(7.43)
C (t) 7.49 J(7.60)
D (dd) 3.90 J(3.98, 10.51)
E (s) 2.20
F (m) 1.87
G (m) 1.63
H (s) 1.55
I (m) 1.24
J (dd) 0.94 J(6.63, 12.42)
1.86
0.95
1.99
1.00
0.85
1.02
1.95
2.98
1.29
5.97
f1 (ppm)

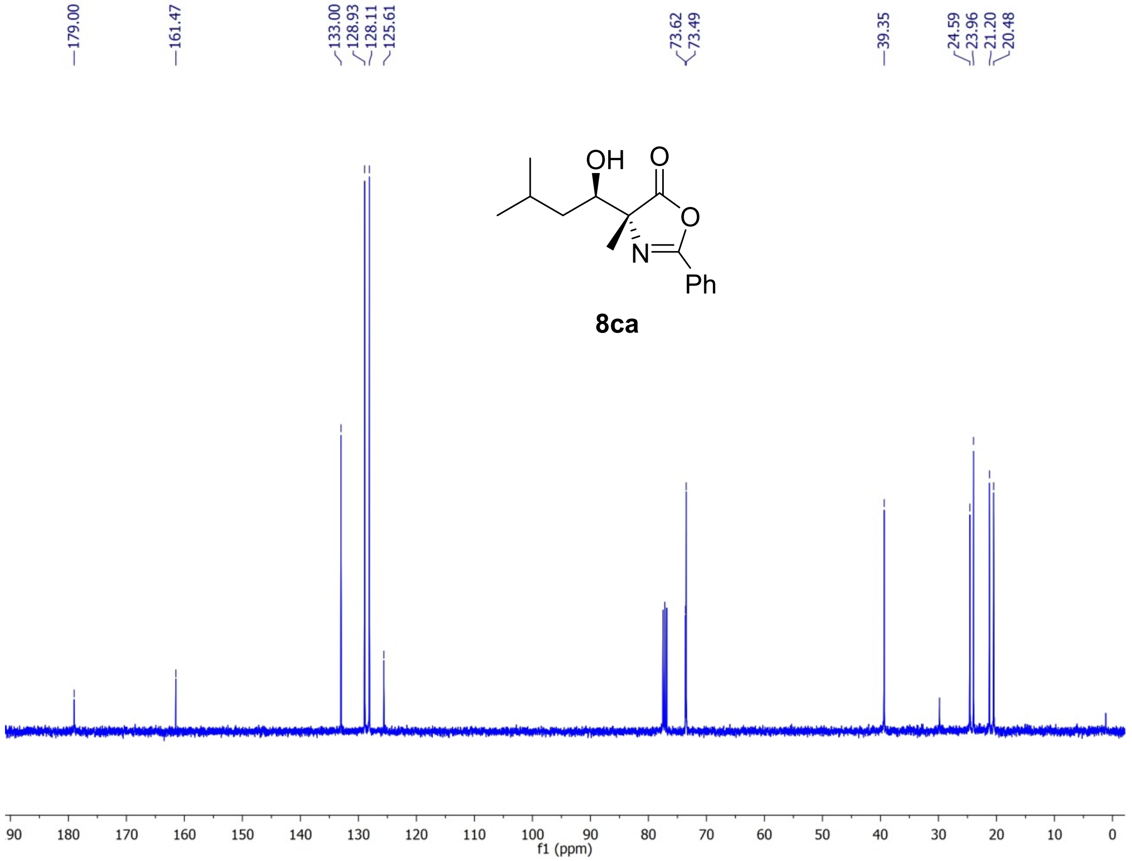
179.00
161.47
133.00
128.93
128.11
125.61
73.62
73.49
39.35
24.59
23.96
21.20
20.48
OH O
O
N
Ph
8ca
f1 (ppm)

Data File C:\CHEM32\1\DATA\YZHENG\3-78RAC_1.D
Sample Name: 3-78rac

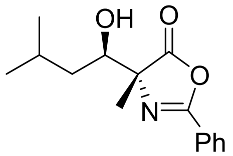

8ca

Racemic

===

Acq. Operator : yang
Acq. Instrument : Instrument 1 Location : Vial 61
Injection Date : 8/15/2011 10:48:35 PM
Inj Volume : 5 µl
Acq. Method : C:\CHEM32\1\METHODS\METHOD1.M
Last changed : 8/15/2011 10:29:10 PM by yang
(modified after loading)
Analysis Method : C:\CHEM32\1\METHODS\METHOD1.M
Last changed : 8/30/2011 2:14:12 PM by xiao
(modified after loading)
Sample Info : AD-H, Hex:IPA = 99:1, 1mL/min, 254 nm, 45 bar, Left

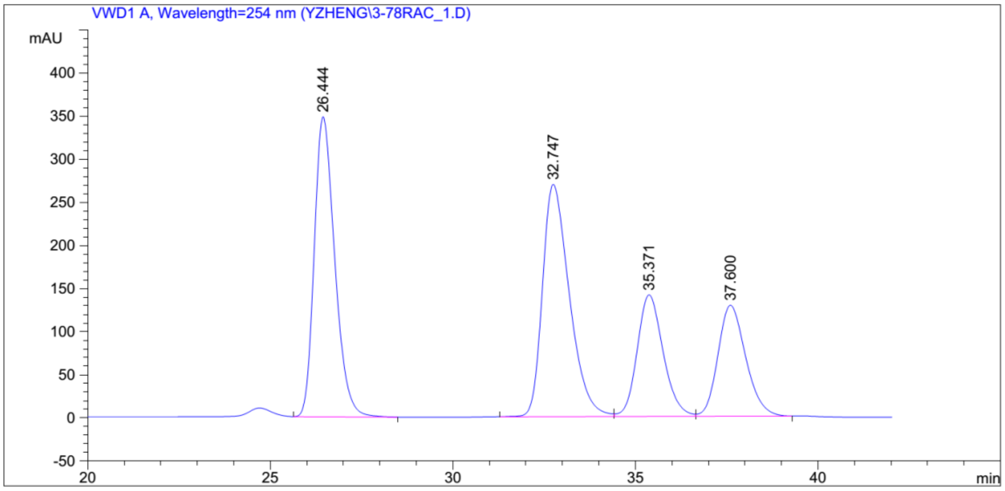

===

Area Percent Report

===

Sorted By : Signal
Multiplier : 1.0000
Dilution : 1.0000
Sample Amount : 1.00000 [ng/ul] (not used in calc.)
Use Multiplier & Dilution Factor with ISTDs

Signal 1: VWD1 A, Wavelength=254 nm

Peak #	RetTime [min]	Type	Width [min]	Area mAU *s	Height [mAU]	Area %
1	26.444	VB	0.6009	1.35843e4	348.07138	33.0314
2	32.747	BV	0.7833	1.37832e4	269.39120	33.5150
3	35.371	VV	0.7583	6964.91602	140.99208	16.9358
4	37.600	VB	0.8142	6792.98926	128.61781	16.5177

Totals : 4.11254e4 887.07248

===

*** End of Report ***

Instrument 1 8/30/2011 2:14:20 PM xiao Page 1 of 1

Data File C:\CHEM32\1\DATA\YZHENG\3-85.D
Sample Name: 3-85

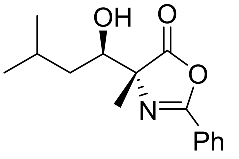

8ca

95% ee, *anti*/*syn* = 98/2

===

Acq. Operator : yang
Acq. Instrument : Instrument 1   Location : Vial 51
Injection Date : 8/30/2011 11:48:31 AM
Inj Volume : 5 µl
Acq. Method : C:\CHEM32\1\METHODS\METHOD1.M
Last changed : 8/30/2011 11:00:10 AM by yang
(modified after loading)
Analysis Method : C:\CHEM32\1\METHODS\METHOD1.M
Last changed : 8/30/2011 2:13:00 PM by xiao
(modified after loading)
Sample Info : AD-H, Hex:IPA = 99:1, 1 mL/min, 254 nm, 44 bar, Left

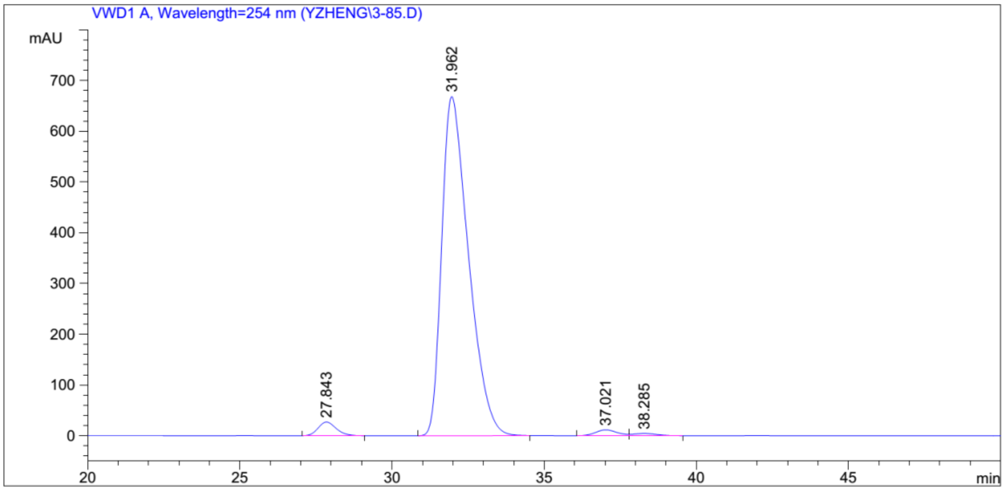

===

Area Percent Report

===

Sorted By : Signal
Multiplier : 1.0000
Dilution : 1.0000
Sample Amount : 1.00000 [ng/ul] (not used in calc.)
Use Multiplier & Dilution Factor with ISTDs

Signal 1: VWD1 A, Wavelength=254 nm

Peak #	RetTime [min]	Type	Width [min]	Area mAU *s	Height [mAU]	Area %
1	27.843	BB	0.6423	1125.41760	26.98795	2.6308
2	31.962	BB	0.9270	4.08507e4	668.41473	95.4950
3	37.021	BV	0.7711	567.44141	11.40712	1.3265
4	38.285	VB	0.7853	234.28592	4.43397	0.5477

Totals : 4.27778e4 711.24377

===

*** End of Report ***

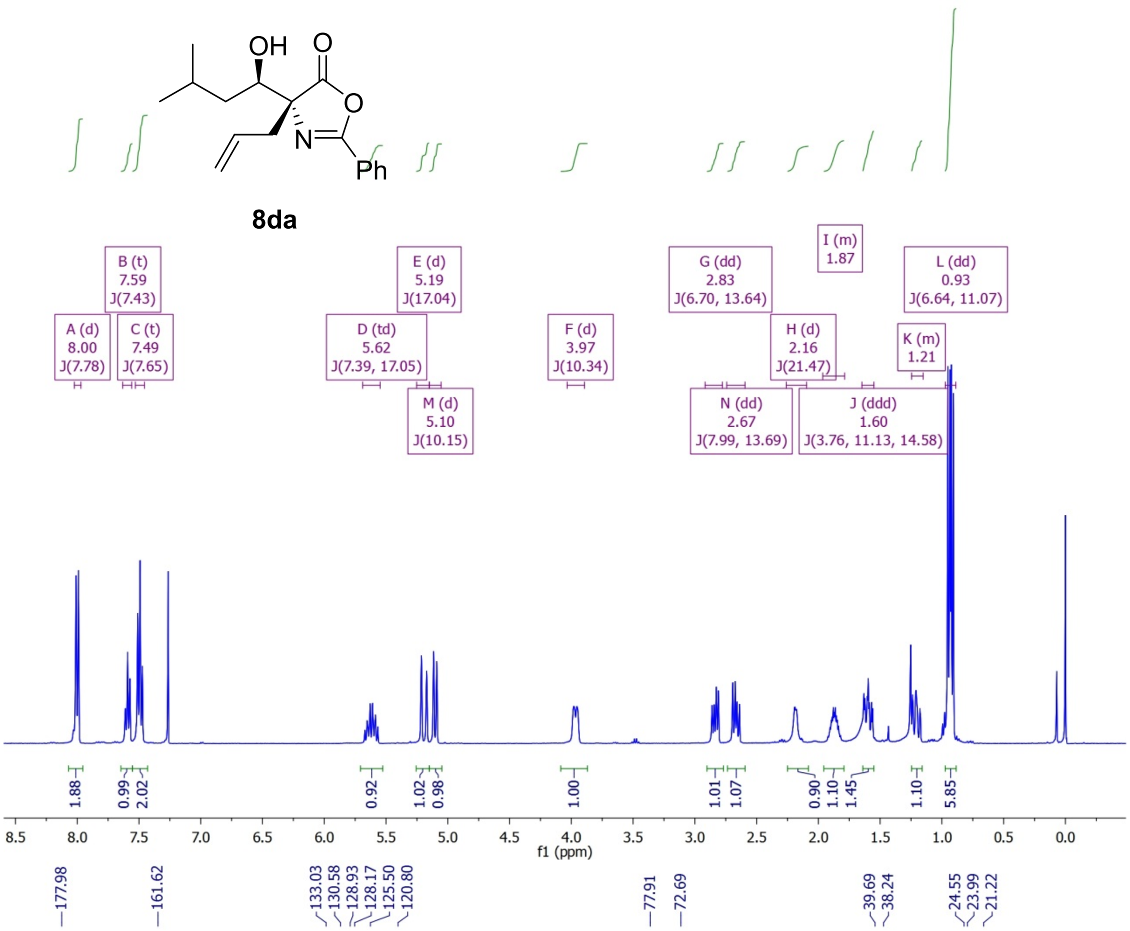
OH
O
O
N
Ph
8da
A (d) 8.00 J(7.78)
B (t) 7.59 J(7.43)
C (t) 7.49 J(7.65)
D (td) 5.62 J(7.39, 17.05)
E (d) 5.19 J(17.04)
M (d) 5.10 J(10.15)
F (d) 3.97 J(10.34)
G (dd) 2.83 J(6.70, 13.64)
N (dd) 2.67 J(7.99, 13.69)
H (d) 2.16 J(21.47)
I (m) 1.87
J (ddd) 1.60 J(3.76, 11.13, 14.58)
K (m) 1.21
L (dd) 0.93 J(6.64, 11.07)
1.88
0.99
2.02
0.92
1.02
0.98
1.00
1.01
1.07
0.90
1.10
1.45
1.10
5.85
8.5 8.0 7.5 7.0 6.5 6.0 5.5 5.0 4.5 4.0 3.5 3.0 2.5 2.0 1.5 1.0 0.5 0.0
f1 (ppm)
177.98
161.62
133.03
130.58
128.93
128.17
125.50
120.80
77.91
72.69
39.69
38.24
24.55
23.99
21.22

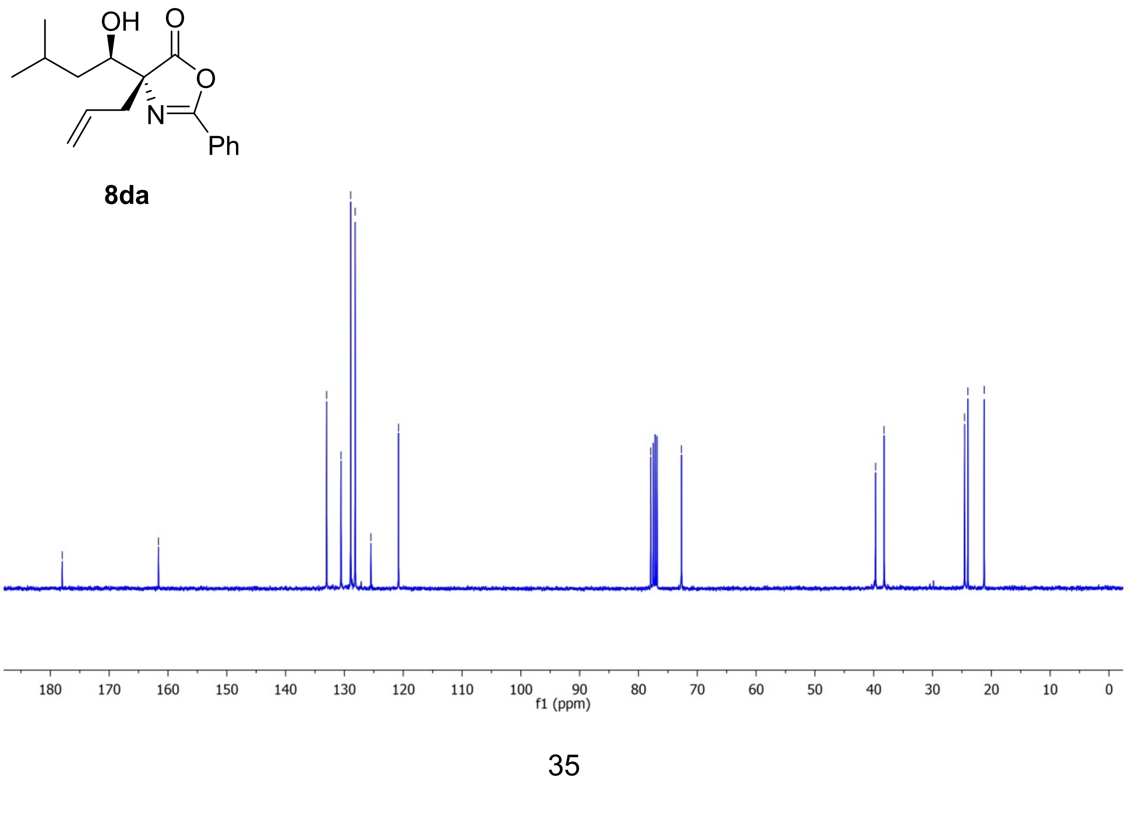
OH
O
O
N
Ph
8da
180 170 160 150 140 130 120 110 100 90 80 70 60 50 40 30 20 10 0
f1 (ppm)

Data File C:\CHEM32\1\DATA\YZHENG\2-68RAC_1.D
Sample Name: 2-68rac

===

Acq. Operator : yang
Acq. Instrument : Instrument 1 Location : -
Injection Date : 1/13/2011 11:35:19 AM
Acq. Method : C:\CHEM32\1\METHODS\METHOD-1_LC.M
Last changed : 1/13/2011 10:39:23 AM by yang
(modified after loading)
Analysis Method : C:\CHEM32\1\METHODS\WASH.M
Last changed : 8/29/2011 10:29:09 AM by yang
(modified after loading)
Sample Info : AD-H, Hex:IPA = 96:4, 1 mL/min, 254 nm, 40 bar, Right

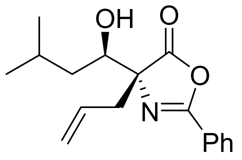

8da

Racemic

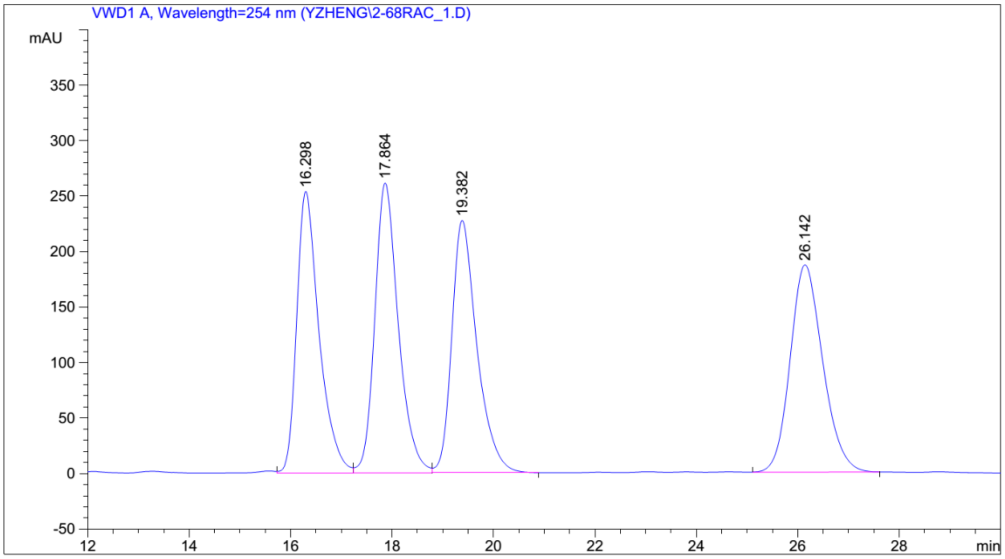

===

Area Percent Report

===

Sorted By : Signal
Multiplier : 1.0000
Dilution : 1.0000
Use Multiplier & Dilution Factor with ISTDs

Signal 1: VWD1 A, Wavelength=254 nm

Peak #	RetTime [min]	Type	Width [min]	Area mAU *s	Height [mAU]	Area %
1	16.298	VV	0.4573	7755.66309	253.59787	23.9816
2	17.864	VV	0.4866	8413.77637	261.07626	26.0166
3	19.382	VB	0.5166	7826.41553	227.39288	24.2004
4	26.142	BB	0.6843	8344.20117	186.77571	25.8014

Totals : 3.23401e4 928.84273

===

*** End of Report ***

Data File C:\CHEM32\1\DATA\YZHENG\2-66_1.D
Sample Name: 2-66

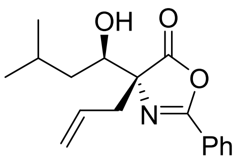

8da

94% ee, *anti*/*syn* = 98/2

===

Acq. Operator : yang
Acq. Instrument : Instrument 1 Location : -
Injection Date : 1/13/2011 12:11:33 PM
Acq. Method : C:\CHEM32\1\METHODS\METHOD-1_LC.M
Last changed : 1/13/2011 12:12:37 PM by yang
(modified after loading)
Analysis Method : C:\CHEM32\1\METHODS\WASH.M
Last changed : 8/29/2011 10:28:01 AM by yang
(modified after loading)
Sample Info : AD-H, Hex:IPA = 96:4, 1 mL/min, 254 nm, 40 bar, Right

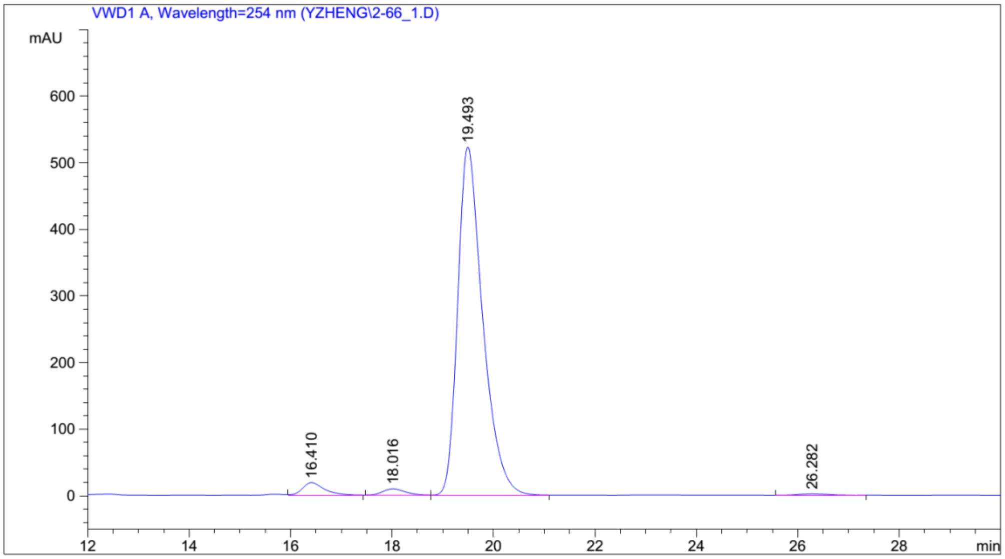

===

Area Percent Report

===

Sorted By : Signal
Multiplier : 1.0000
Dilution : 1.0000
Use Multiplier & Dilution Factor with ISTDs

Signal 1: VWD1 A, Wavelength=254 nm

Peak #	RetTime [min]	Type	Width [min]	Area mAU *s	Height [mAU]	Area %
1	16.410	VB	0.4480	578.97461	19.11654	3.0657
2	18.016	BV	0.4745	304.32053	9.79644	1.6114
3	19.493	VB	0.5146	1.79064e4	522.82092	94.8149
4	26.282	BB	0.6333	95.94662	2.20955	0.5080

Totals : 1.88856e4 553.94345

===

*** End of Report ***

Instrument 1 8/29/2011 10:28:15 AM yang Page 1 of 1

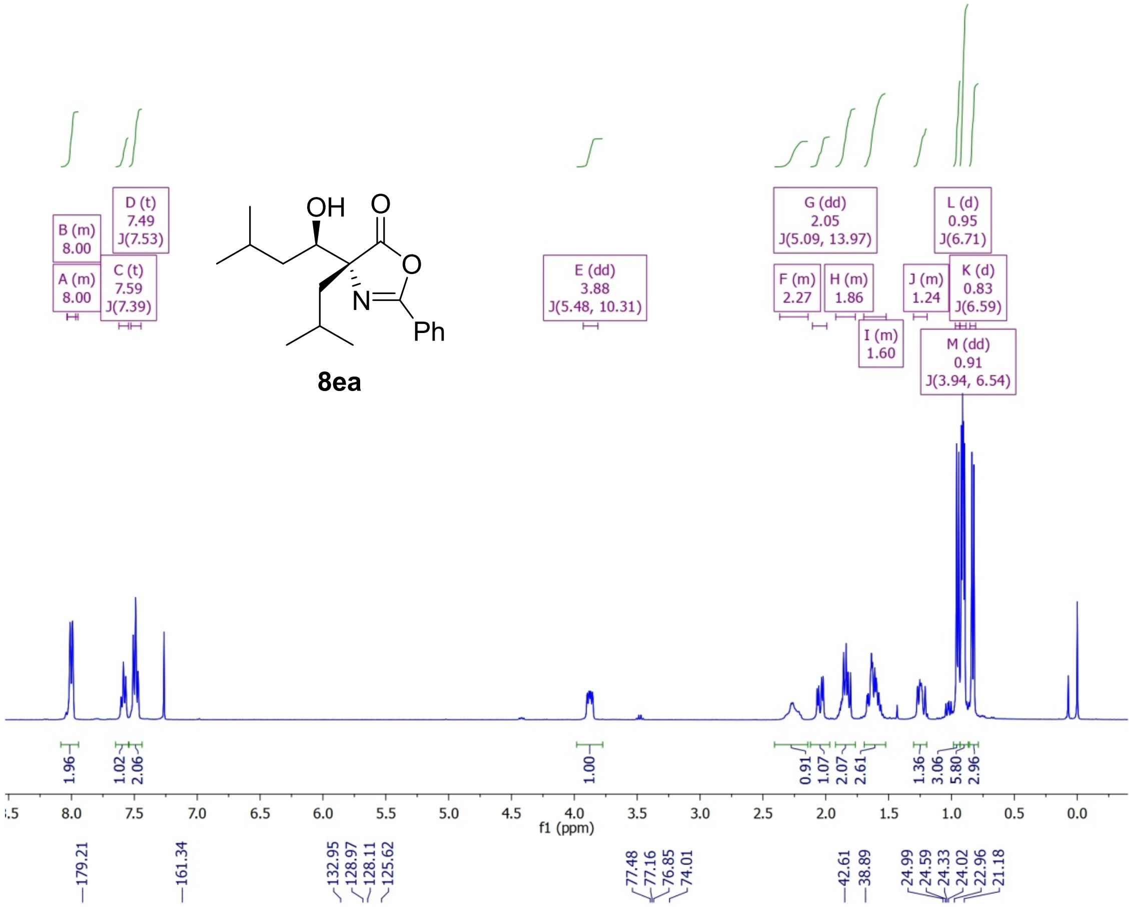

B (m) 8.00
A (m) 8.00
D (t) 7.49 J(7.53)
C (t) 7.59 J(7.39)
OH
O
O
N
Ph
8ea
E (dd) 3.88 J(5.48, 10.31)
G (dd) 2.05 J(5.09, 13.97)
F (m) 2.27
H (m) 1.86
I (m) 1.60
J (m) 1.24
L (d) 0.95 J(6.71)
K (d) 0.83 J(6.59)
M (dd) 0.91 J(3.94, 6.54)
1.96
1.02
2.06
1.00
0.91
1.07
2.07
2.61
1.36
3.06
5.80
2.96
f1 (ppm)
179.21
161.34
132.95
128.97
128.11
125.62
77.48
77.16
76.85
74.01
42.61
38.89
24.99
24.59
24.33
24.02
22.96
21.18

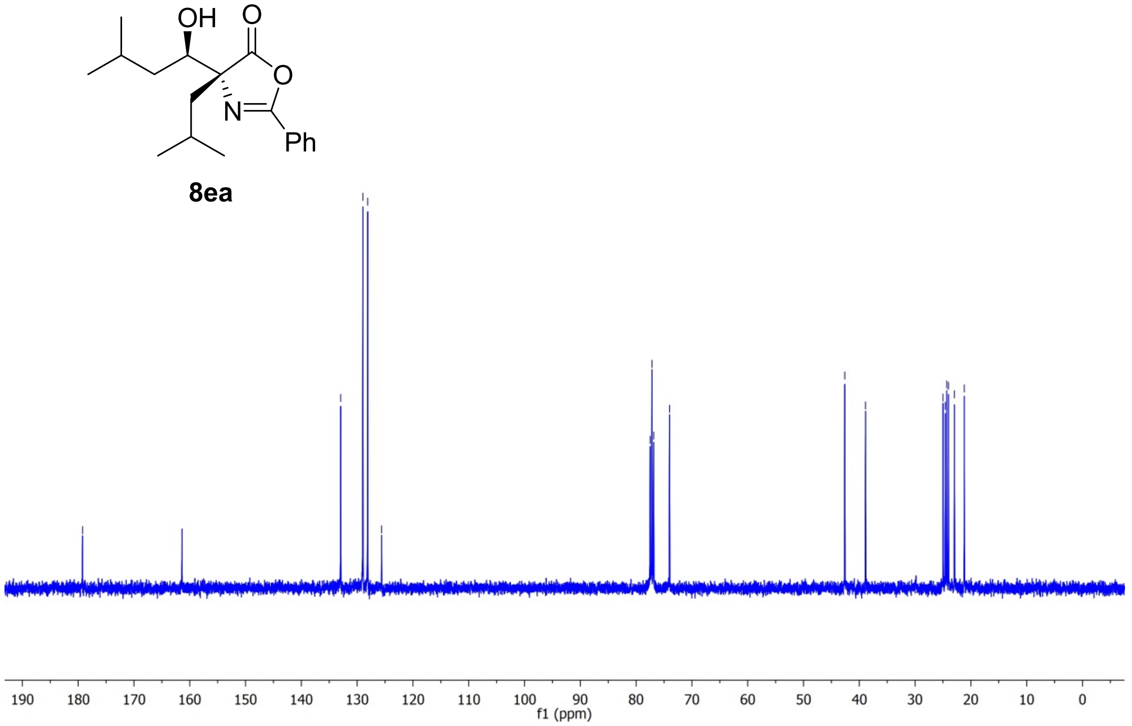

OH
O
O
N
Ph
8ea
f1 (ppm)

```
Data File C:\CHEM32\1\DATA\YZHENG\3-60RAC_1.D
 Sample Name: 3-60rac

    =====================================================================
    Acq. Operator   : yang
    Acq. Instrument : Instrument 1                    Location :   -
    Injection Date  : 7/28/2011 11:21:48 AM
    Acq. Method     : C:\CHEM32\1\METHODS\METHOD-1_LC.M
    Last changed    : 7/28/2011 10:55:57 AM by yang
                      (modified after loading)
    Analysis Method : C:\CHEM32\1\METHODS\METHOD-1_LC.M
    Last changed    : 8/1/2011 12:58:37 PM by yang
                      (modified after loading)
    Sample Info     : AD-H, Hex:IPA = 96:4, 1.0 mL/min, 254 nm, 39 bar, Right
```

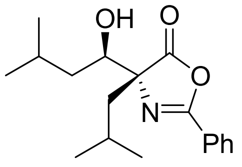

8ea

Racemic

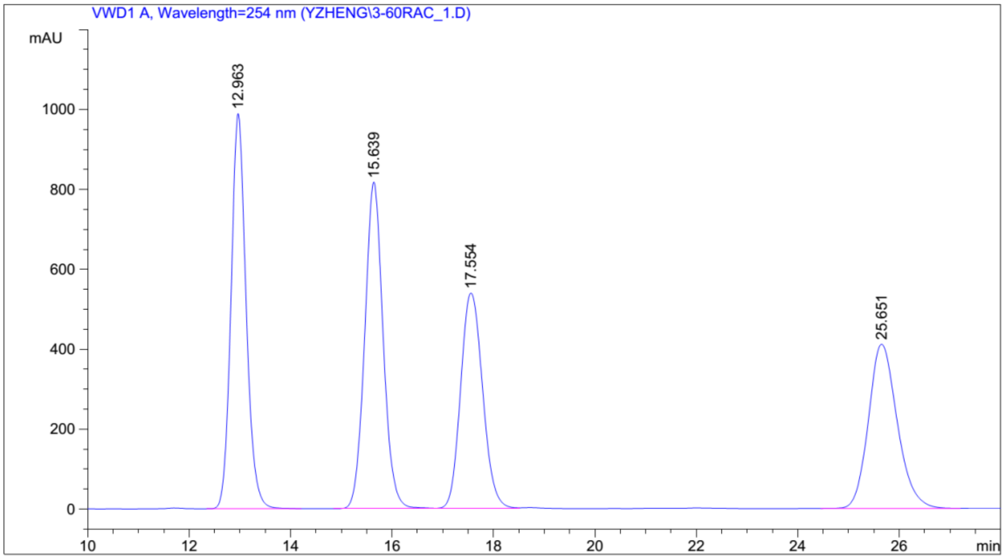


```
    =====================================================================
                             Area Percent Report
    =====================================================================

    Sorted By             :      Signal
    Multiplier            :      1.0000
    Dilution              :      1.0000
    Use Multiplier & Dilution Factor with ISTDs


    Signal 1: VWD1 A, Wavelength=254 nm
```

Peak #	RetTime [min]	Type	Width [min]	Area mAU *s	Height [mAU]	Area %
1	12.963	BB	0.3132	2.03779e4	989.14178	27.7510
2	15.639	BV	0.3821	2.05489e4	817.36218	27.9840
3	17.554	VV	0.4744	1.62889e4	539.76801	22.1826
4	25.651	BB	0.5942	1.62153e4	411.04782	22.0823
Totals :				7.34310e4	2757.31979	

```
    =====================================================================
                             *** End of Report ***
```

Data File C:\CHEM32\1\DATA\YZHENG\3-59_1.D
Sample Name: 3-59

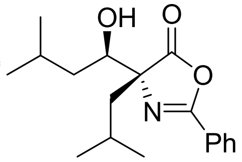

8ea

93% ee, *anti*/*syn* = 97.5/2.5

===

```
Acq. Operator   : yang
Acq. Instrument : Instrument 1                    Location :   -
Injection Date  : 7/29/2011 12:00:11 AM
Acq. Method     : C:\CHEM32\1\METHODS\METHOD-1_LC.M
Last changed    : 7/28/2011 11:37:54 PM by yang
                  (modified after loading)
Analysis Method : C:\CHEM32\1\METHODS\METHOD-1_LC.M
Last changed    : 8/1/2011 12:56:49 PM by yang
                  (modified after loading)
Sample Info     : AD-H, Hex:IPA = 96:4, 1.0 mL/min, 254 nm, 39 bar, Right
```

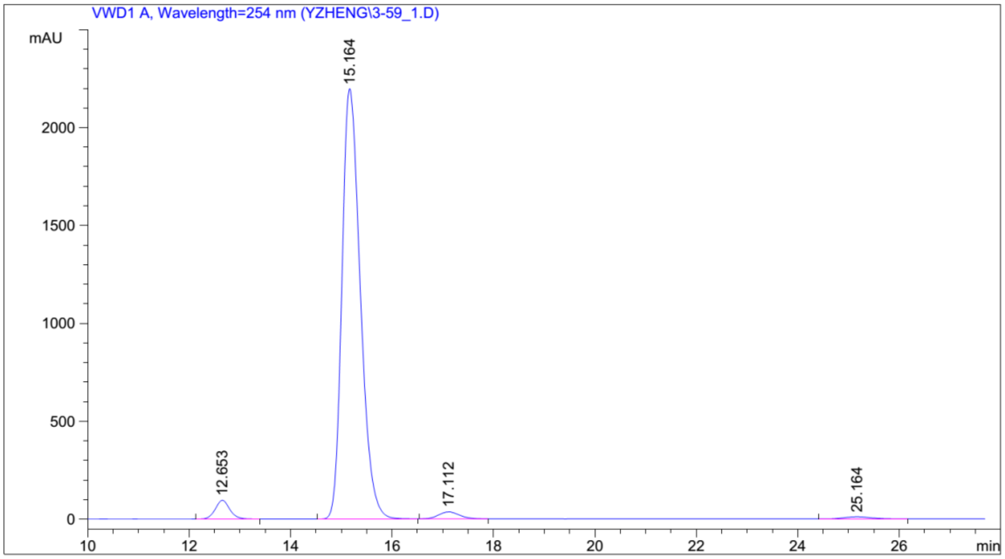

===

Area Percent Report

===

```
Sorted By             :      Signal
Multiplier            :      1.0000
Dilution              :      1.0000
Use Multiplier & Dilution Factor with ISTDs
```

Signal 1: VWD1 A, Wavelength=254 nm

Peak #	RetTime [min]	Type	Width [min]	Area mAU *s	Height [mAU]	Area %
1	12.653	BB	0.3011	1925.33533	96.53149	3.2858
2	15.164	BB	0.3891	5.51910e4	2197.30835	94.1898
3	17.112	BB	0.4435	1045.66577	36.33644	1.7845
4	25.164	BB	0.5818	433.50134	11.44081	0.7398

Totals : 5.85955e4 2341.61710

===

*** End of Report ***

Instrument 1 8/1/2011 12:58:37 PM yang   Page 1 of 1

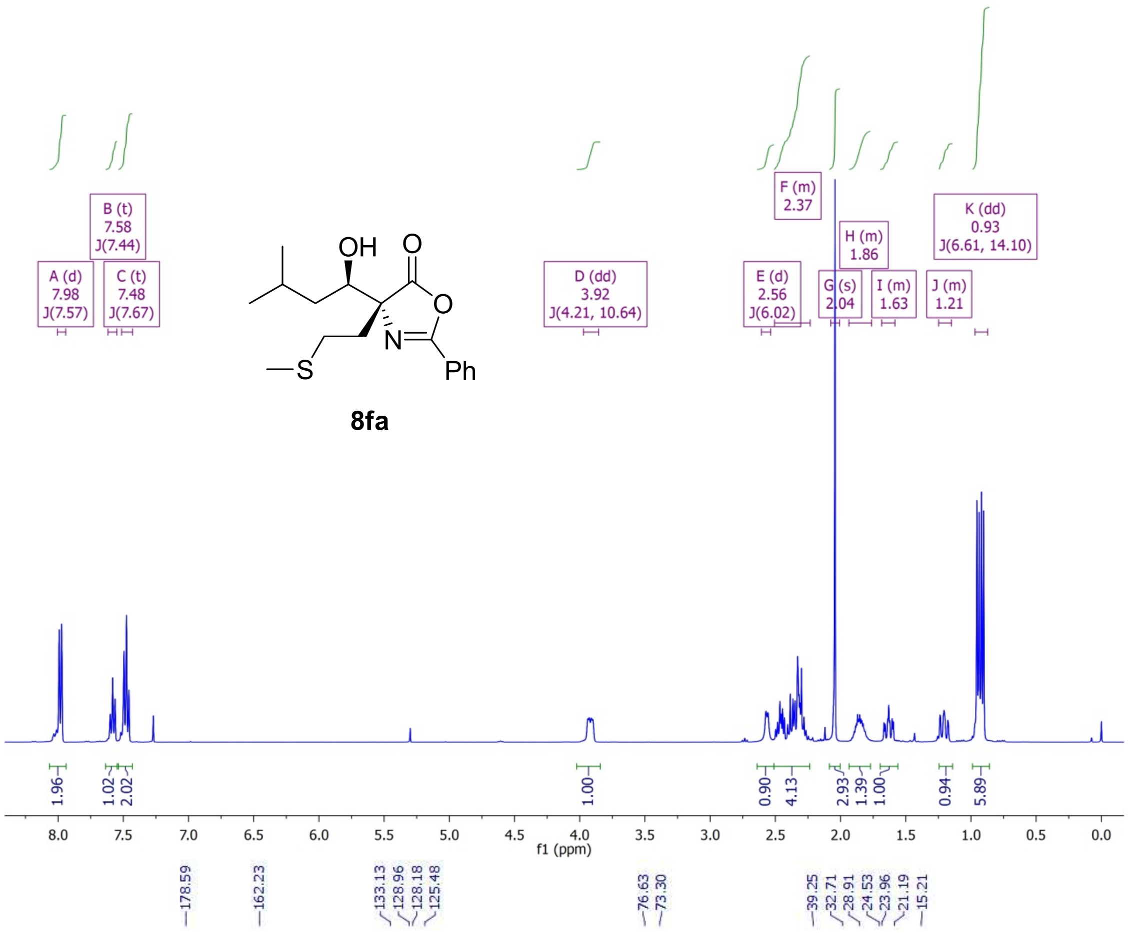

A (d)
7.98
J(7.57)
B (t)
7.58
J(7.44)
C (t)
7.48
J(7.67)
D (dd)
3.92
J(4.21, 10.64)
E (d)
2.56
J(6.02)
F (m)
2.37
G (s)
2.04
H (m)
1.86
I (m)
1.63
J (m)
1.21
K (dd)
0.93
J(6.61, 14.10)
OH
O
O
N
S
Ph
8fa
1.96
1.02
2.02
1.00
0.90
4.13
2.93
1.39
1.00
0.94
5.89
f1 (ppm)

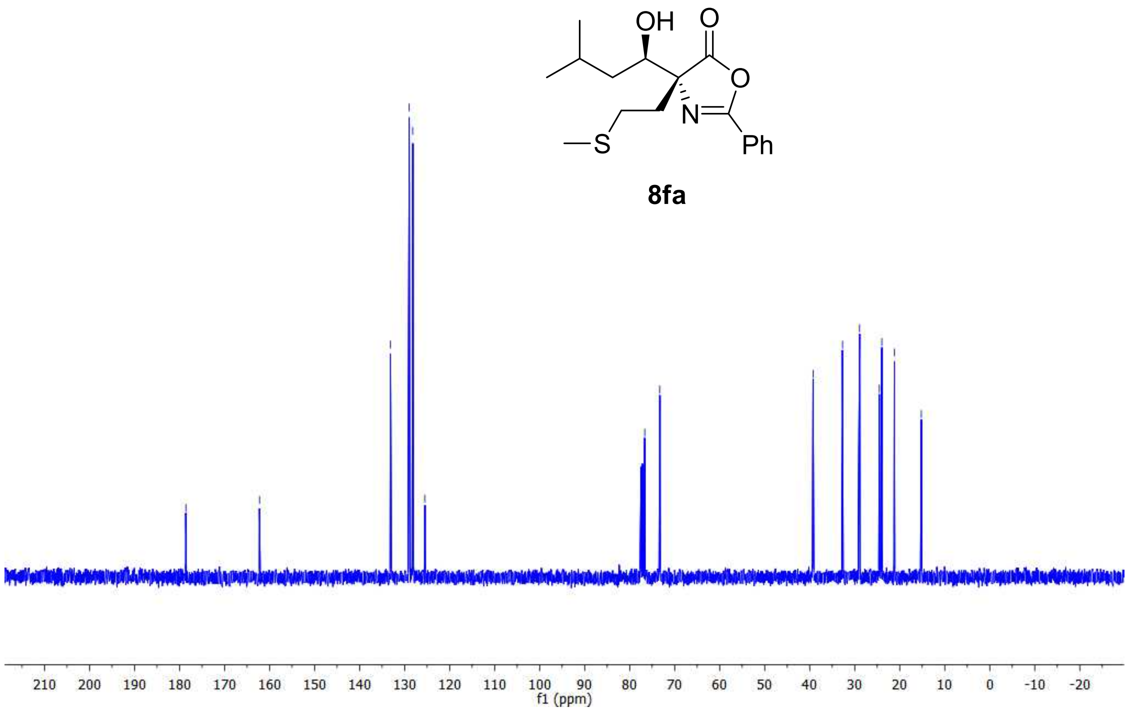

178.59
162.23
133.13
128.96
128.18
125.48
76.63
73.30
39.25
32.71
28.91
24.53
23.96
21.19
15.21
OH
O
O
N
S
Ph
8fa
f1 (ppm)

Data File C:\CHEM32\1\DATA\YZHENG\2-84RAC_3.D
Sample Name: 2-84rac

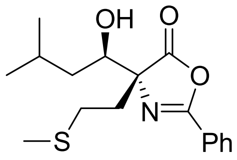

8fa

Racemic

===

```
Acq. Operator   : yang
Acq. Instrument : Instrument 1                    Location :   -
Injection Date  : 1/27/2011 6:18:51 PM
Acq. Method     : C:\CHEM32\1\METHODS\METHOD-1_LC.M
Last changed    : 1/27/2011 6:08:05 PM by yang
                  (modified after loading)
Analysis Method : C:\CHEM32\1\METHODS\WASH.M
Last changed    : 8/31/2011 11:14:37 AM by yang
                  (modified after loading)
Sample Info     : AD-H, Hex:IPA = 96.5:3.5, 1 mL/min, 254 nm, 39 bar, Rig
                  ht
```

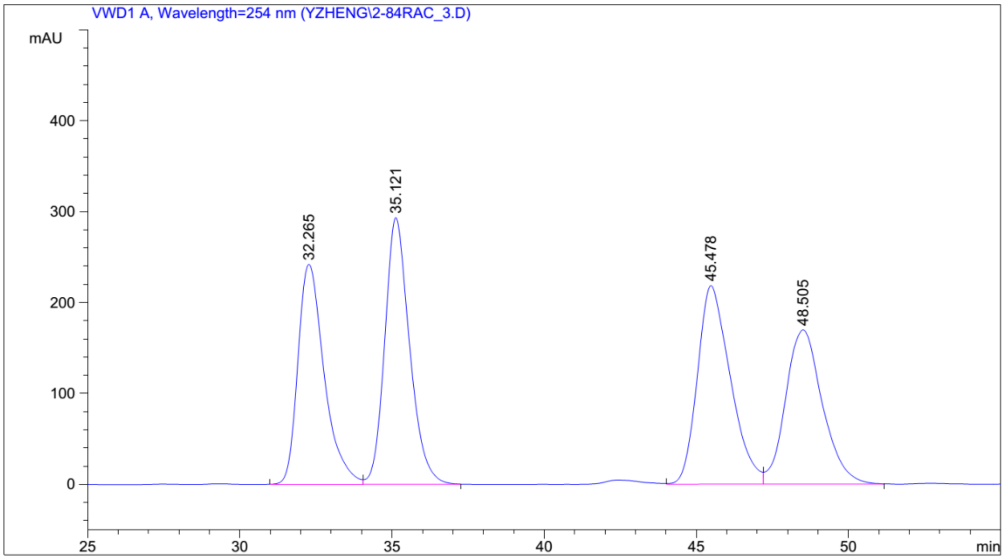

===

Area Percent Report

===

```
Sorted By             :      Signal
Multiplier            :      1.0000
Dilution              :      1.0000
Use Multiplier & Dilution Factor with ISTDs
```

Signal 1: VWD1 A, Wavelength=254 nm

Peak #	RetTime [min]	Type	Width [min]	Area mAU *s	Height [mAU]	Area %
1	32.265	BV	0.9091	1.45334e4	241.92194	23.5229
2	35.121	VB	0.8421	1.65952e4	293.30771	26.8599
3	45.478	VV	1.0786	1.64092e4	218.47810	26.5589
4	48.505	VB	1.2715	1.42463e4	169.54929	23.0582

Totals : 6.17841e4 923.25703

===

*** End of Report ***

Instrument 1 8/31/2011 11:14:50 AM yang   Page   1 of 1

```
Data File C:\CHEM32\1\DATA\YZHENG\3-66.D
 Sample Name: 3-66

    =====================================================================
    Acq. Operator   : yang
    Acq. Instrument : Instrument 1                    Location :   -
    Injection Date  : 8/15/2011 12:54:41 PM
    Acq. Method     : C:\CHEM32\1\METHODS\METHOD-1_LC.M
    Last changed    : 8/15/2011 11:01:25 AM by yang
                      (modified after loading)
    Analysis Method : C:\CHEM32\1\METHODS\WASH.M
    Last changed    : 8/15/2011 5:03:25 PM by yang
                      (modified after loading)
    Sample Info     : AD-H, Hex:IPA = 96.5:3.5, 1 mL/min, 254 nm, 40 bar, Rig
                      ht
```

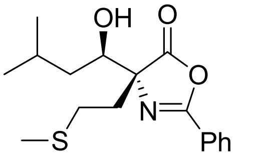

8fa

92% ee, *anti*/*syn* = 97/3

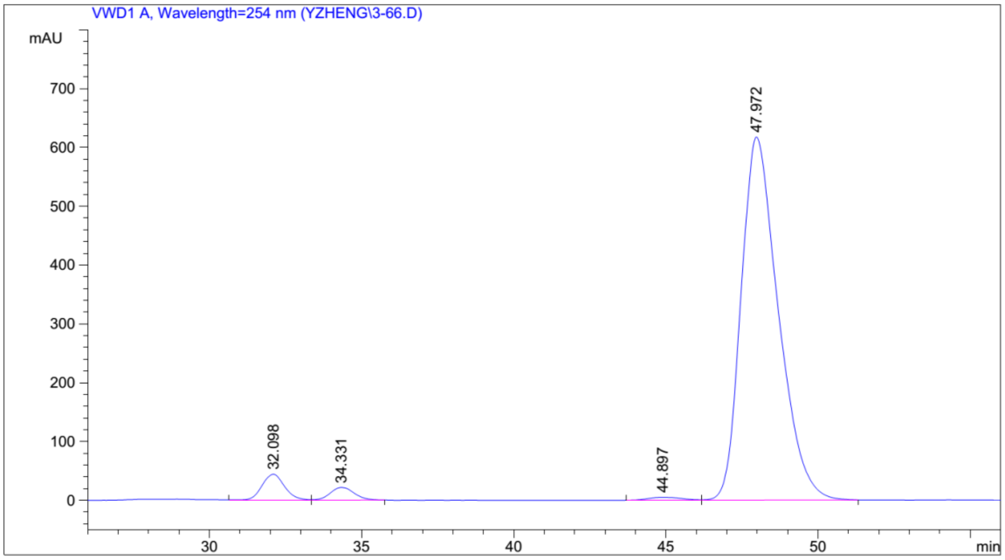


```
    =====================================================================
                             Area Percent Report
    =====================================================================

    Sorted By             :      Signal
    Multiplier            :      1.0000
    Dilution              :      1.0000
    Use Multiplier & Dilution Factor with ISTDs


    Signal 1: VWD1 A, Wavelength=254 nm

    Peak RetTime Type  Width     Area      Height     Area
      #   [min]        [min]  mAU   *s    [mAU   ]      %
    ----|-------|----|-------|----------|----------|--------|
       1  32.098 VV    0.8085 2286.37842   44.11148   4.0707
       2  34.331 VB    0.8557 1195.80103   21.49694   2.1290
       3  44.897 BV    0.9209  370.31412    4.92522   0.6593
       4  47.972 VB    1.2040 5.23148e4   617.45117  93.1410

    Totals :                  5.61673e4   687.98482

    =====================================================================
                              *** End of Report ***
```

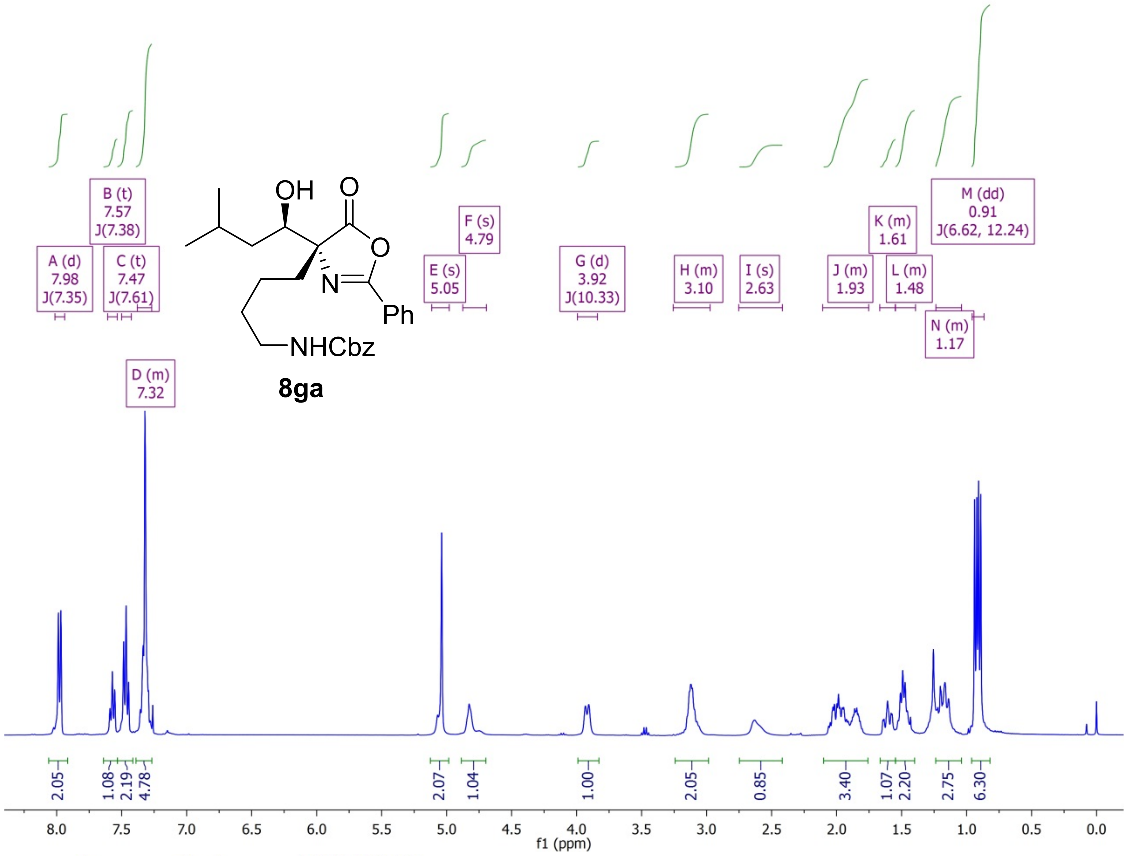

OH
O
O
N
Ph
NHCbz
8ga
A (d) 7.98 J(7.35)
B (t) 7.57 J(7.38)
C (t) 7.47 J(7.61)
D (m) 7.32
E (s) 5.05
F (s) 4.79
G (d) 3.92 J(10.33)
H (m) 3.10
I (s) 2.63
J (m) 1.93
K (m) 1.61
L (m) 1.48
M (dd) 0.91 J(6.62, 12.24)
N (m) 1.17
f1 (ppm)

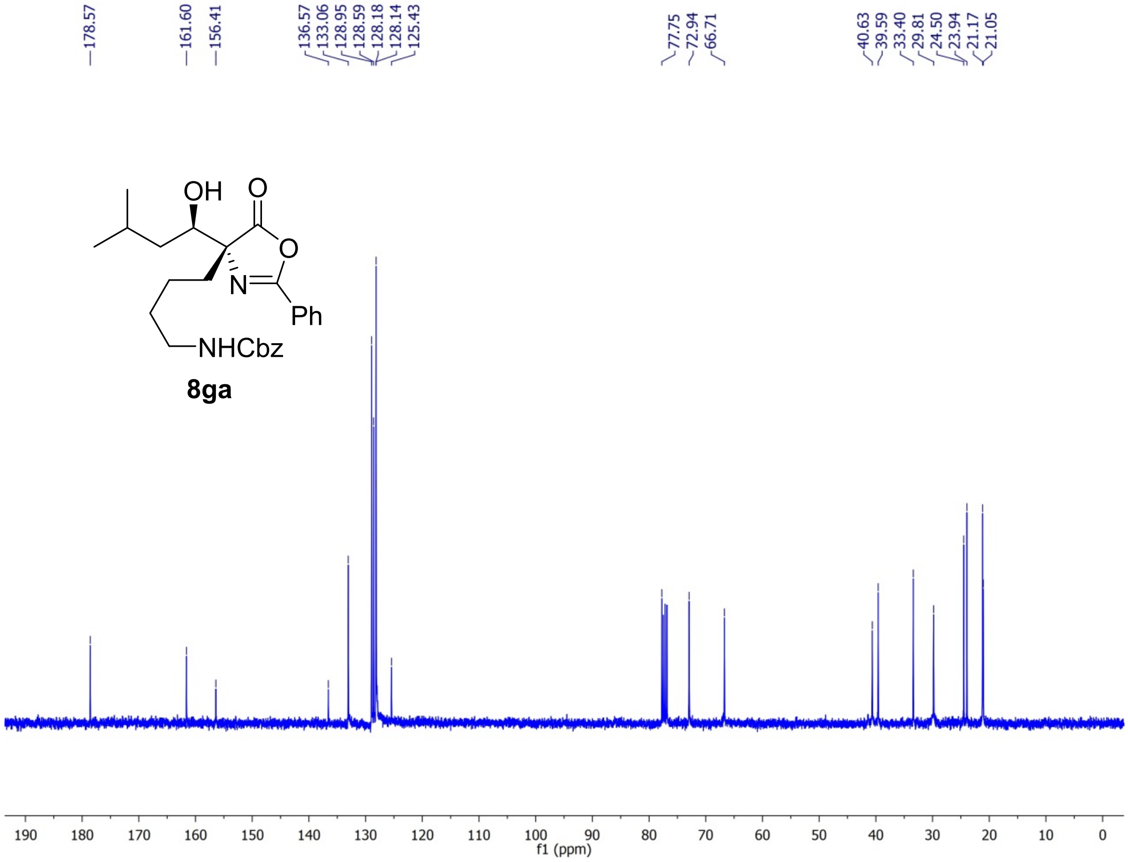

178.57
161.60
156.41
136.57
133.06
128.95
128.59
128.18
128.14
125.43
77.75
72.94
66.71
40.63
39.59
33.40
29.81
24.50
23.94
21.17
21.05
OH
O
O
N
Ph
NHCbz
8ga
f1 (ppm)

Data File C:\CHEM32\1\DATA\YZHENG\3-72RAC_1.D
Sample Name: 2-72rac

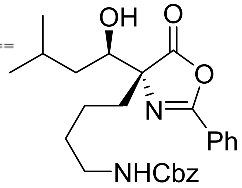

8ga

Racemic

===

Acq. Operator : yang
Acq. Instrument : Instrument 1 Location : -
Injection Date : 8/11/2011 3:44:19 PM
Acq. Method : C:\CHEM32\1\METHODS\METHOD-1_LC.M
Last changed : 8/11/2011 2:59:20 PM by yang
(modified after loading)
Analysis Method : C:\CHEM32\1\METHODS\METHOD-1_LC.M
Last changed : 8/11/2011 6:27:53 PM by yang
(modified after loading)
Sample Info : AD-H, Hex:IPA = 87:13, 1 mL/min, 254 nm, 43 bar, Right

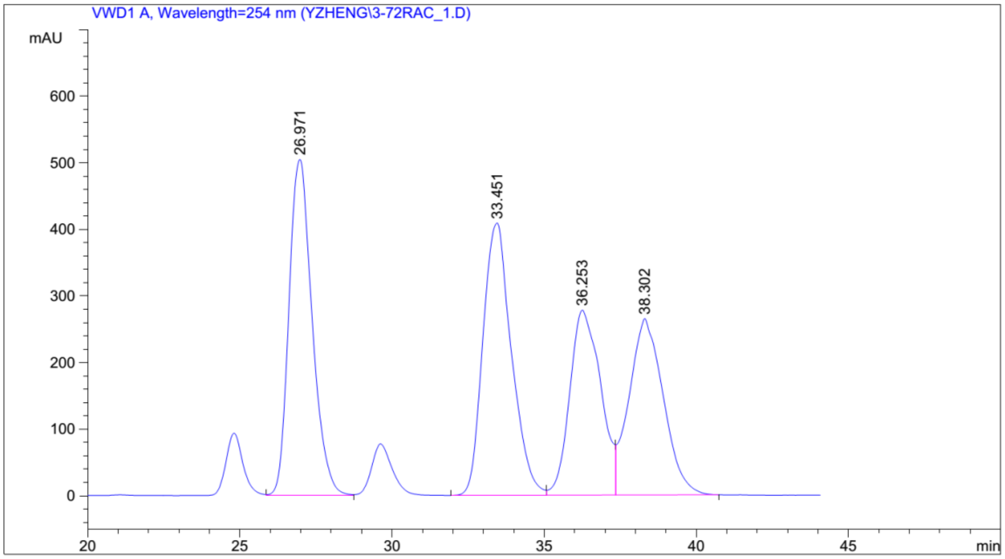

===

Area Percent Report

===

Sorted By : Signal
Multiplier : 1.0000
Dilution : 1.0000
Use Multiplier & Dilution Factor with ISTDs

Signal 1: VWD1 A, Wavelength=254 nm

Peak #	RetTime [min]	Type	Width [min]	Area mAU *s	Height [mAU]	Area %
1	26.971	VV	0.7904	2.67048e4	504.80145	28.5004
2	33.451	BV	1.0009	2.66542e4	408.83020	28.4464
3	36.253	VV	0.9966	1.96091e4	277.86295	20.9276
4	38.302	VB	1.0630	2.07317e4	264.91617	22.1257

Totals : 9.36999e4 1456.41077

===

*** End of Report ***

Instrument 1 8/11/2011 6:27:58 PM yang Page 1 of 1

Data File C:\CHEM32\1\DATA\YZHENG\3-71.D
Sample Name: 3-71

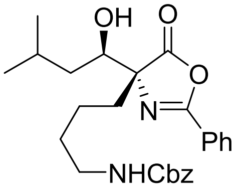

8ga

94% ee, *anti*/*syn* = 97.5/2.5

```
=====================================================================
Acq. Operator   : yang
Acq. Instrument : Instrument 1                    Location :   -
Injection Date  : 8/11/2011 5:17:51 PM
Acq. Method     : C:\CHEM32\1\METHODS\METHOD-1_LC.M
Last changed    : 8/11/2011 4:31:09 PM by yang
                  (modified after loading)
Analysis Method : C:\CHEM32\1\METHODS\METHOD-1_LC.M
Last changed    : 8/11/2011 6:25:54 PM by yang
                  (modified after loading)
Sample Info     : AD-H, Hex:IPA = 87:13, 1 mL/min, 254 nm, 43 bar, Right
```

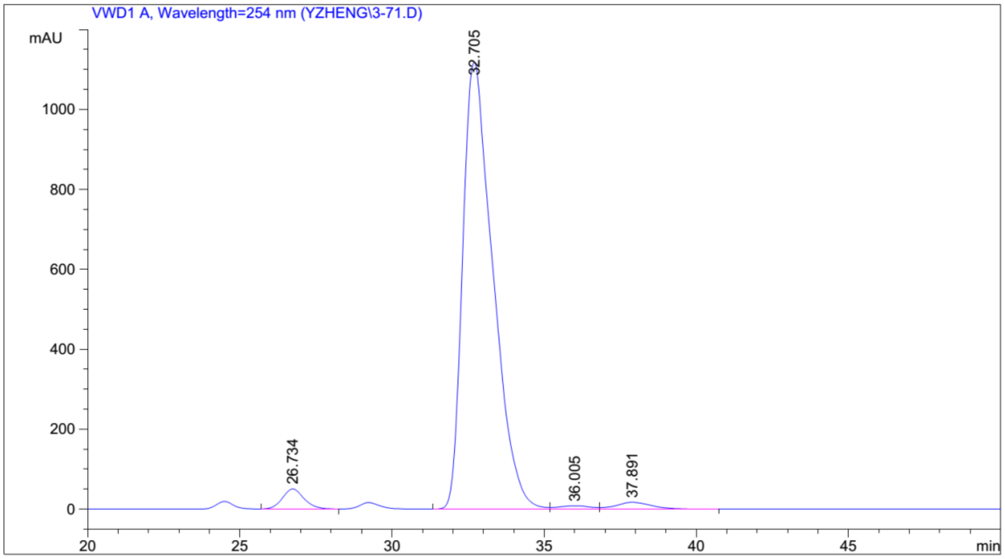


```
=====================================================================
                         Area Percent Report
=====================================================================

Sorted By             :      Signal
Multiplier            :      1.0000
Dilution              :      1.0000
Use Multiplier & Dilution Factor with ISTDs


Signal 1: VWD1 A, Wavelength=254 nm
```

Peak #	RetTime [min]	Type	Width [min]	Area mAU *s	Height [mAU]	Area %
1	26.734	BB	0.7527	2456.97339	50.22921	3.0263
2	32.705	BB	0.9954	7.68489e4	1118.29761	94.6549
3	36.005	BV	0.9143	567.14624	7.90414	0.6986
4	37.891	VB	0.9769	1315.50061	16.92087	1.6203

Totals : 8.11885e4 1193.35183

```
=====================================================================
                         *** End of Report ***
```

Instrument 1 8/11/2011 6:26:12 PM yang   Page   1 of 1

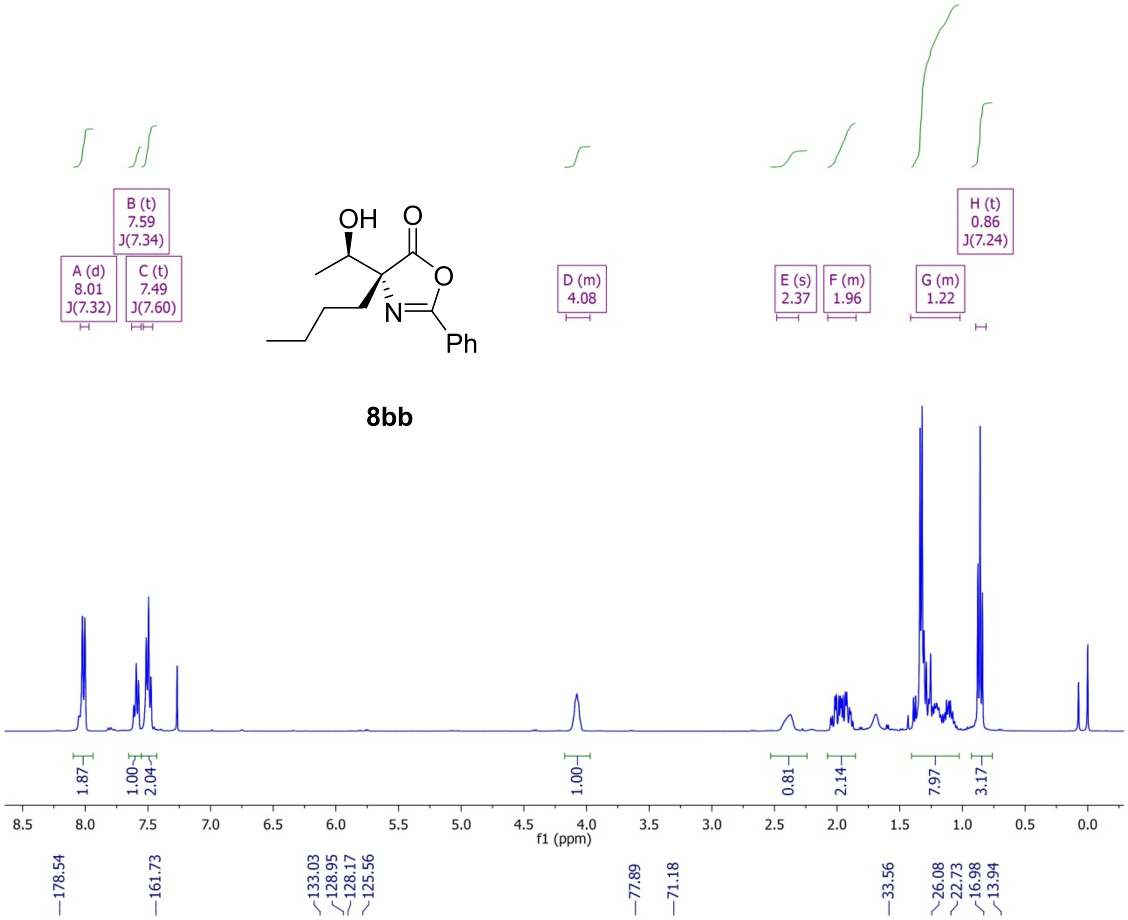
A (d)
8.01
J(7.32)
B (t)
7.59
J(7.34)
C (t)
7.49
J(7.60)
OH
O
O
N
Ph
8bb
D (m)
4.08
E (s)
2.37
F (m)
1.96
G (m)
1.22
H (t)
0.86
J(7.24)
1.87
1.00
2.04
1.00
0.81
2.14
7.97
3.17
f1 (ppm)
178.54
161.73
133.03
128.95
128.17
125.56
77.89
71.18
33.56
26.08
22.73
16.98
13.94

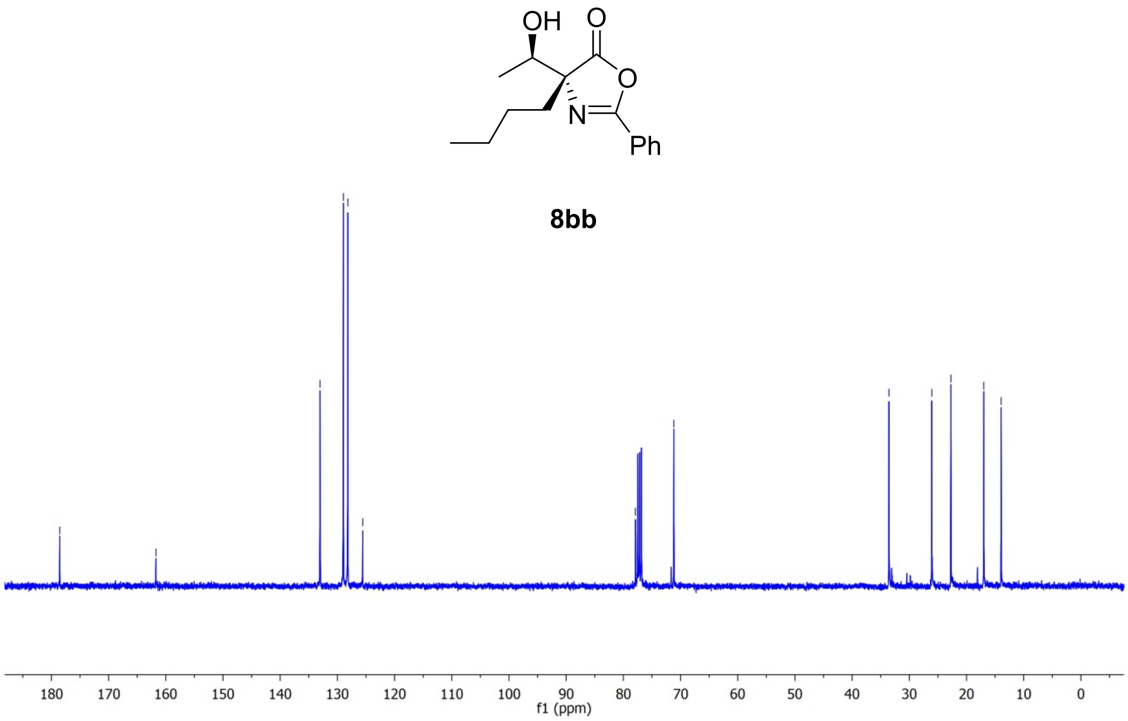
OH
O
O
N
Ph
8bb
f1 (ppm)

Data File C:\CHEM32\1\DATA\YZHENG\3-79RAC.D
Sample Name: 3-79rac

===

Acq. Operator : yang
Acq. Instrument : Instrument 1 Location : -
Injection Date : 8/16/2011 5:35:41 PM
Acq. Method : C:\CHEM32\1\METHODS\METHOD-1_LC.M
Last changed : 8/16/2011 5:15:02 PM by yang
(modified after loading)
Analysis Method : C:\CHEM32\1\METHODS\WASH.M
Last changed : 8/16/2011 10:22:15 PM by yang
(modified after loading)
Sample Info : OJ-H+OJ, Hex:IPA = 96:4, 0.8 mL/min, 254 nm, 51 bar, Right

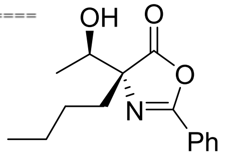

8bb

Racemic

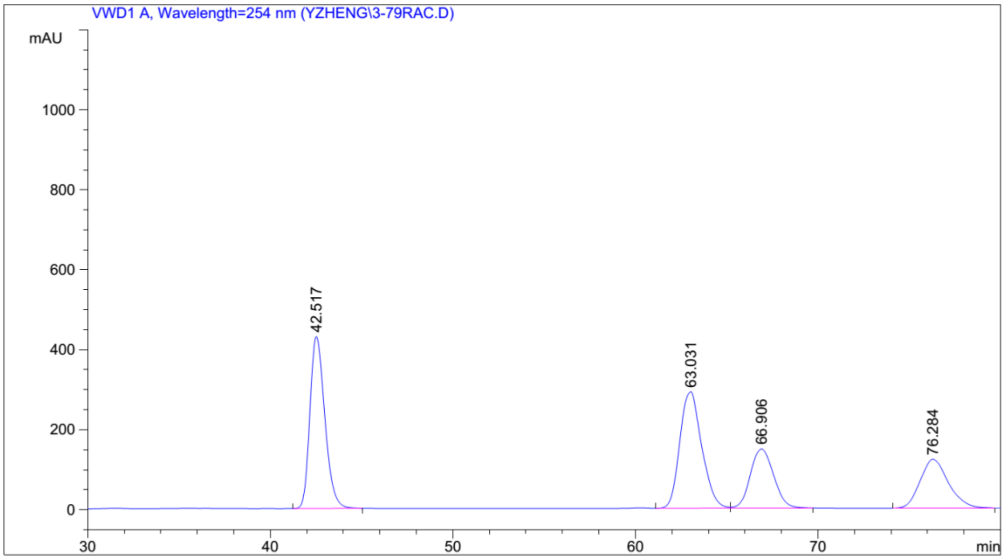

===

Area Percent Report

===

Sorted By : Signal
Multiplier : 1.0000
Dilution : 1.0000
Use Multiplier & Dilution Factor with ISTDs

Signal 1: VWD1 A, Wavelength=254 nm

Peak #	RetTime [min]	Type	Width [min]	Area mAU *s	Height [mAU]	Area %
1	42.517	BB	0.8859	2.44250e4	429.74283	32.5804
2	63.031	VV	1.2815	2.43056e4	291.49854	32.4211
3	66.906	VB	1.4139	1.31029e4	148.06441	17.4778
4	76.284	BB	1.6199	1.31349e4	122.15461	17.5206

Totals : 7.49684e4 991.46038

===

*** End of Report ***

Instrument 1 8/16/2011 10:22:21 PM yang Page 1 of 1

Data File C:\CHEM32\1\DATA\YZHENG\3-79.D
Sample Name: 3-79

===

```
Acq. Operator   : yang
Acq. Instrument : Instrument 1                   Location :   -
Injection Date  : 8/16/2011 8:43:08 PM
Acq. Method     : C:\CHEM32\1\METHODS\METHOD-1_LC.M
Last changed    : 8/16/2011 5:15:02 PM by yang
                  (modified after loading)
Analysis Method : C:\CHEM32\1\METHODS\WASH.M
Last changed    : 8/16/2011 10:21:37 PM by yang
                  (modified after loading)
Sample Info     : OJ-H+OJ, Hex:IPA = 96:4, 0.8 mL/min, 254 nm, 52 bar, Ri
                  ght
```

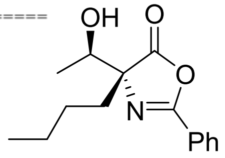

8bb

88% ee, *anti*/*syn* = 90.5/9.5

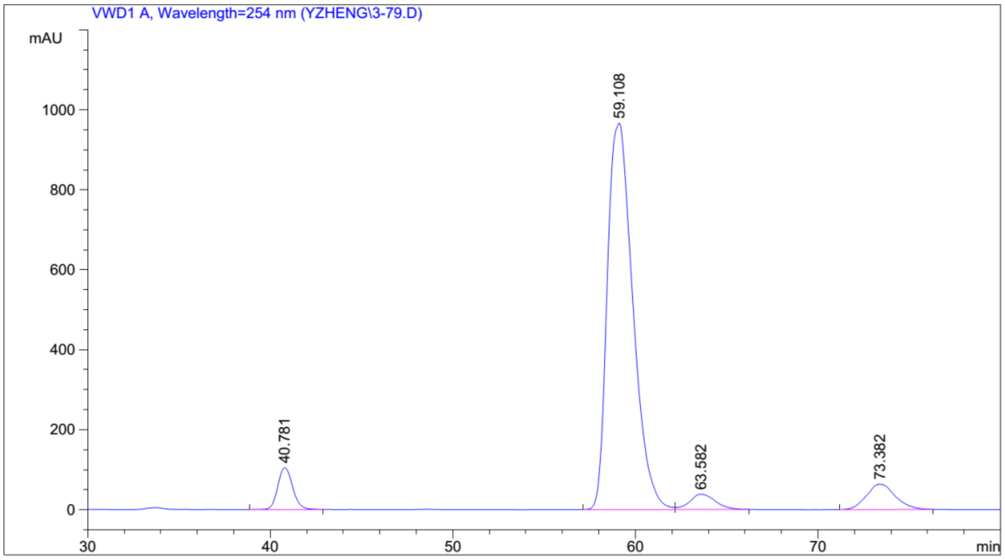

===

Area Percent Report

===

```
Sorted By             :      Signal
Multiplier            :      1.0000
Dilution              :      1.0000
Use Multiplier & Dilution Factor with ISTDs
```

Signal 1: VWD1 A, Wavelength=254 nm

Peak #	RetTime [min]	Type	Width [min]	Area mAU *s	Height [mAU]	Area %
1	40.781	BB	0.8862	5945.25244	104.31563	5.3903
2	59.108	BB	1.3108	9.36414e4	966.55273	84.9013
3	63.582	BB	1.3658	3728.47119	38.25199	3.3805
4	73.382	BB	1.6048	6979.34521	63.24734	6.3279

Totals : 1.10294e5 1172.36769

===

*** End of Report ***

Instrument 1 8/16/2011 10:21:50 PM yang Page 1 of 1

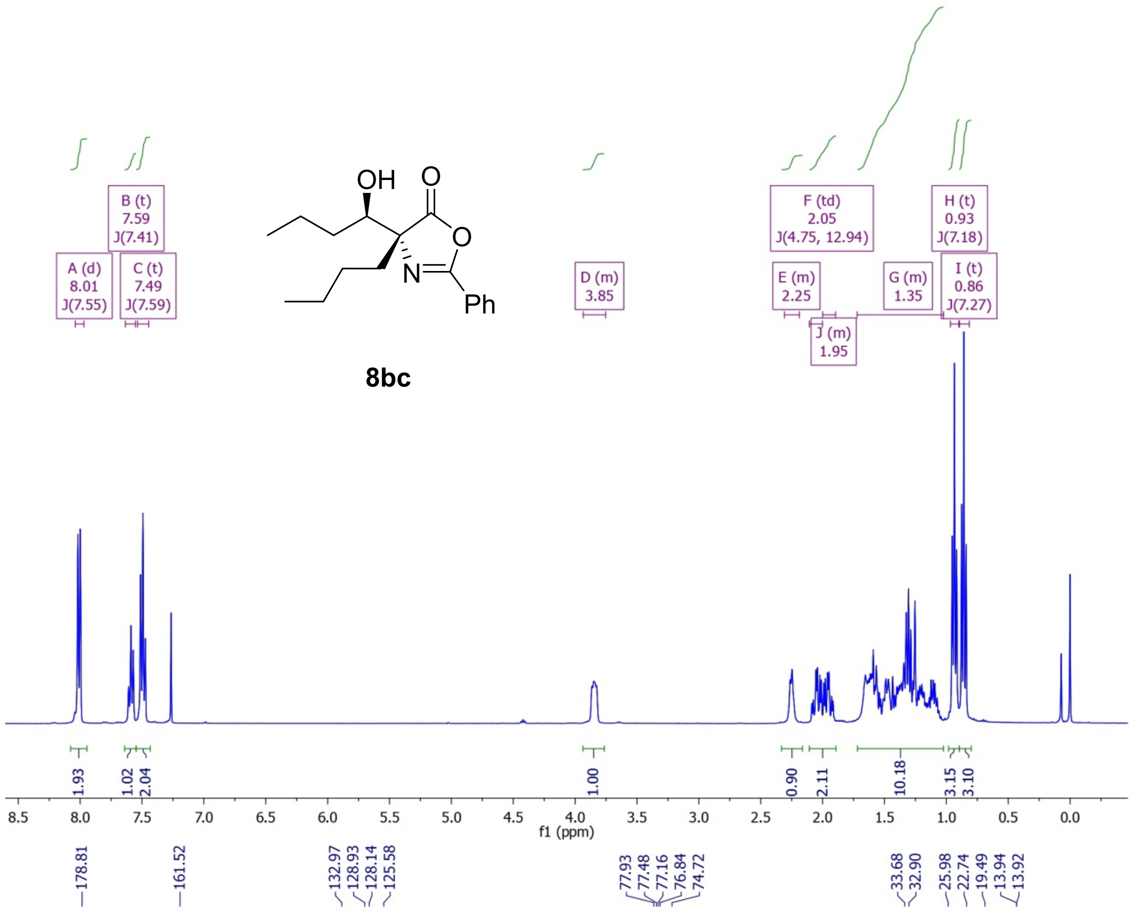
OH
O
O
N
Ph
8bc
A (d)
8.01
J(7.55)
B (t)
7.59
J(7.41)
C (t)
7.49
J(7.59)
D (m)
3.85
E (m)
2.25
F (td)
2.05
J(4.75, 12.94)
J (m)
1.95
G (m)
1.35
H (t)
0.93
J(7.18)
I (t)
0.86
J(7.27)
1.93
1.02
2.04
1.00
0.90
2.11
10.18
3.15
3.10
f1 (ppm)
178.81
161.52
132.97
128.93
128.14
125.58
77.93
77.48
77.16
76.84
74.72
33.68
32.90
25.98
22.74
19.49
13.94
13.92

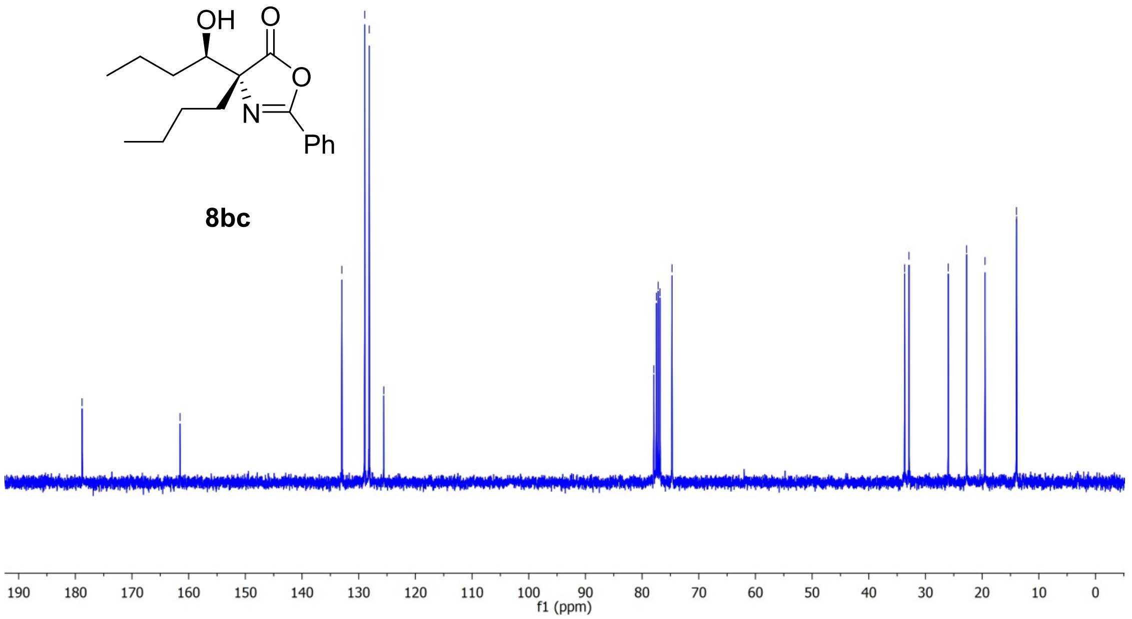
OH
O
O
N
Ph
8bc
f1 (ppm)

Data File C:\CHEM32\1\DATA\YZHENG\3-112RAC_1.D
Sample Name: 3-22

===

Acq. Operator : yang
Acq. Instrument : Instrument 1 Location : -
Injection Date : 10/3/2011 8:02:06 PM
Acq. Method : C:\CHEM32\1\METHODS\YANG.M
Last changed : 10/3/2011 7:27:20 PM by yang
(modified after loading)
Analysis Method : C:\CHEM32\1\METHODS\YANG.M
Last changed : 10/4/2011 12:55:14 PM by yang
(modified after loading)
Sample Info : AD-H+AD, Hex:IPA = 97.2:2.8, 0.8 mL/min, 254 nm, 43 bar , Right

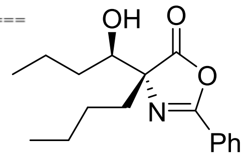

8bc

Racemic

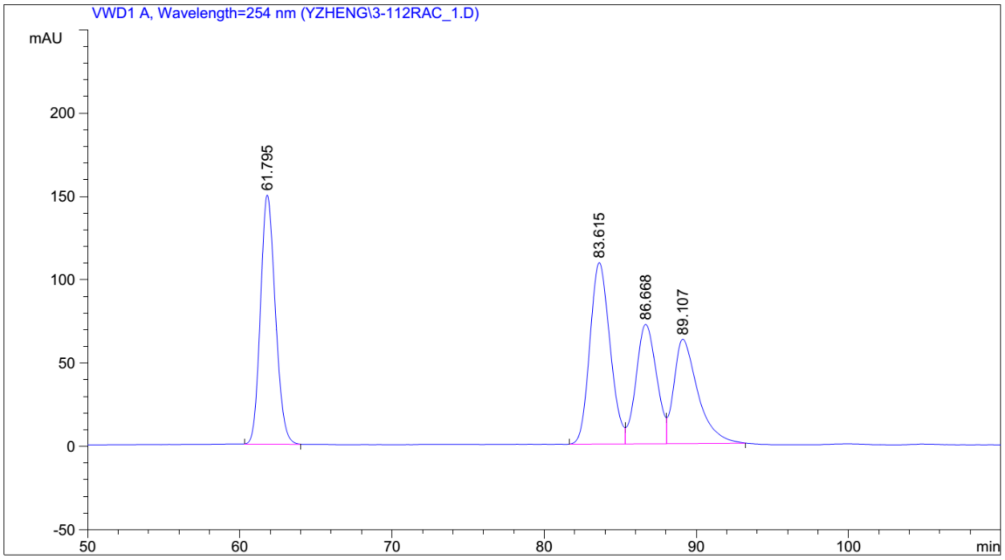

===

Area Percent Report

===

Sorted By : Signal
Multiplier : 1.0000
Dilution : 1.0000
Use Multiplier & Dilution Factor with ISTDs

Signal 1: VWD1 A, Wavelength=254 nm

Peak #	RetTime [min]	Type	Width [min]	Area mAU *s	Height [mAU]	Area %
1	61.795	BB	1.0586	1.01515e4	149.51230	30.1216
2	83.615	BV	1.4019	9997.66309	108.86904	29.6651
3	86.668	VV	1.4206	6694.03369	71.64605	19.8626
4	89.107	VB	1.5286	6858.53320	62.67318	20.3507
Totals :				3.37017e4	392.70057	

===

*** End of Report ***

Data File C:\CHEM32\1\DATA\YZHENG\3-111_1.D
Sample Name: 3-111

===

Acq. Operator : yang
Acq. Instrument : Instrument 1 Location : -
Injection Date : 10/4/2011 1:48:56 PM
Acq. Method : C:\CHEM32\1\METHODS\YANG.M
Last changed : 10/4/2011 1:08:48 PM by yang
(modified after loading)
Analysis Method : C:\CHEM32\1\METHODS\YANG.M
Last changed : 10/4/2011 3:52:38 PM by yang
(modified after loading)
Sample Info : AD-H+AD, Hex:IPA = 97.2:2.8, 0.8 mL/min, 254 nm, 43 bar
, Right

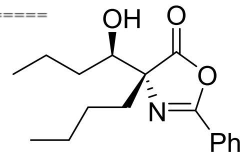

8bc

94% ee, *anti*/*syn* = 97.5/2.5

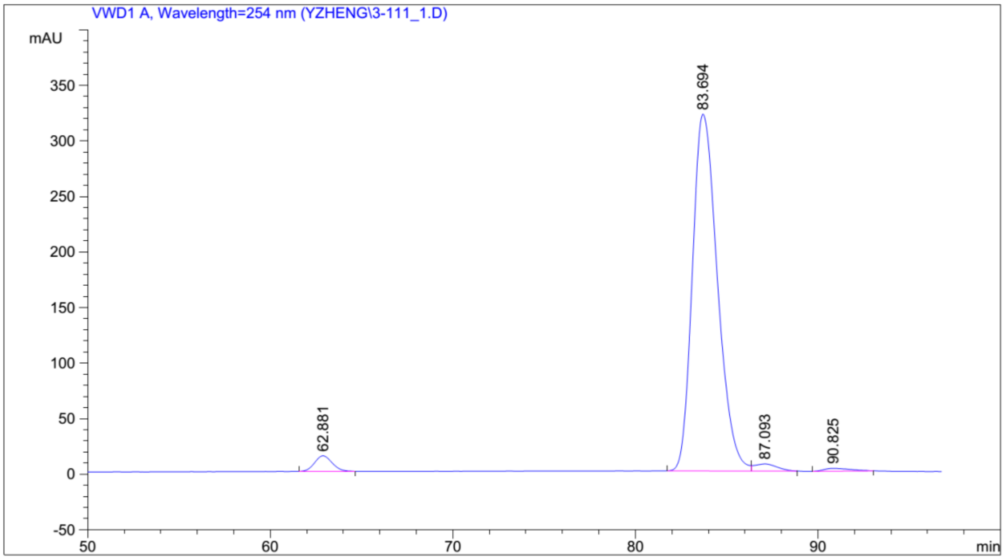

===

Area Percent Report

===

Sorted By : Signal
Multiplier : 1.0000
Dilution : 1.0000
Use Multiplier & Dilution Factor with ISTDs

Signal 1: VWD1 A, Wavelength=254 nm

Peak #	RetTime [min]	Type	Width [min]	Area mAU *s	Height [mAU]	Area %
1	62.881	BB	1.0241	947.42053	14.12772	2.9253
2	83.694	BB	1.4483	3.06197e4	321.25366	94.5426
3	87.093	BB	1.0888	557.07318	6.46186	1.7200
4	90.825	BB	1.1914	263.00912	2.59781	0.8121
Totals :				3.23872e4	344.44105	

===

*** End of Report ***

Data File C:\CHEM32\1\DATA\YZHENG\3-112_1.D
Sample Name: 3-22

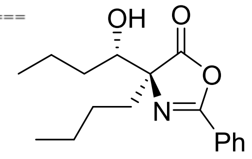

ent-**8bc**

-92% ee, *anti*/*syn* = 97/3

===

```
Acq. Operator   : yang
Acq. Instrument : Instrument 1                    Location :  -
Injection Date  : 10/3/2011 5:01:00 PM
Acq. Method     : C:\CHEM32\1\METHODS\YANG.M
Last changed    : 10/3/2011 4:37:38 PM by yang
                  (modified after loading)
Analysis Method : C:\CHEM32\1\METHODS\YANG.M
Last changed    : 10/4/2011 12:48:26 PM by yang
                  (modified after loading)
Sample Info     : AD-H+AD, Hex:IPA = 97:3, 0.8 mL/min, 254 nm, 43 bar, Ri
                  ght
```

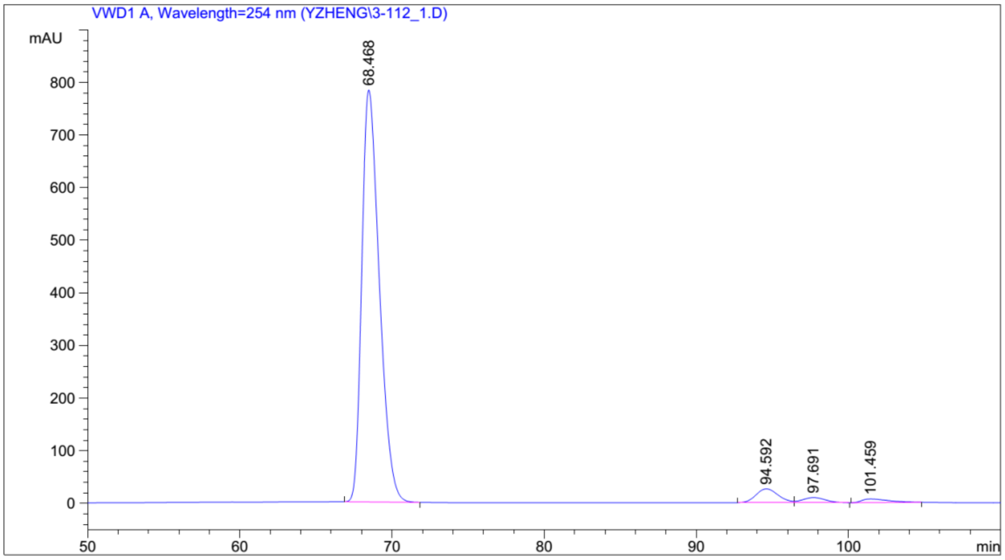

===

Area Percent Report

===

```
Sorted By             :      Signal
Multiplier            :      1.0000
Dilution              :      1.0000
Use Multiplier & Dilution Factor with ISTDs
```

Signal 1: VWD1 A, Wavelength=254 nm

Peak #	RetTime [min]	Type	Width [min]	Area mAU *s	Height [mAU]	Area %
1	68.468	BB	1.2534	6.26077e4	782.81177	93.2501
2	94.592	BV	1.5157	2681.97754	26.33486	3.9946
3	97.691	VB	1.3537	1016.94580	9.79685	1.5147
4	101.459	BB	1.5890	832.90808	6.82155	1.2406

Totals : 6.71396e4 825.76503

===

*** End of Report ***

Instrument 1 10/4/2011 12:52:19 PM yang Page 1 of 1

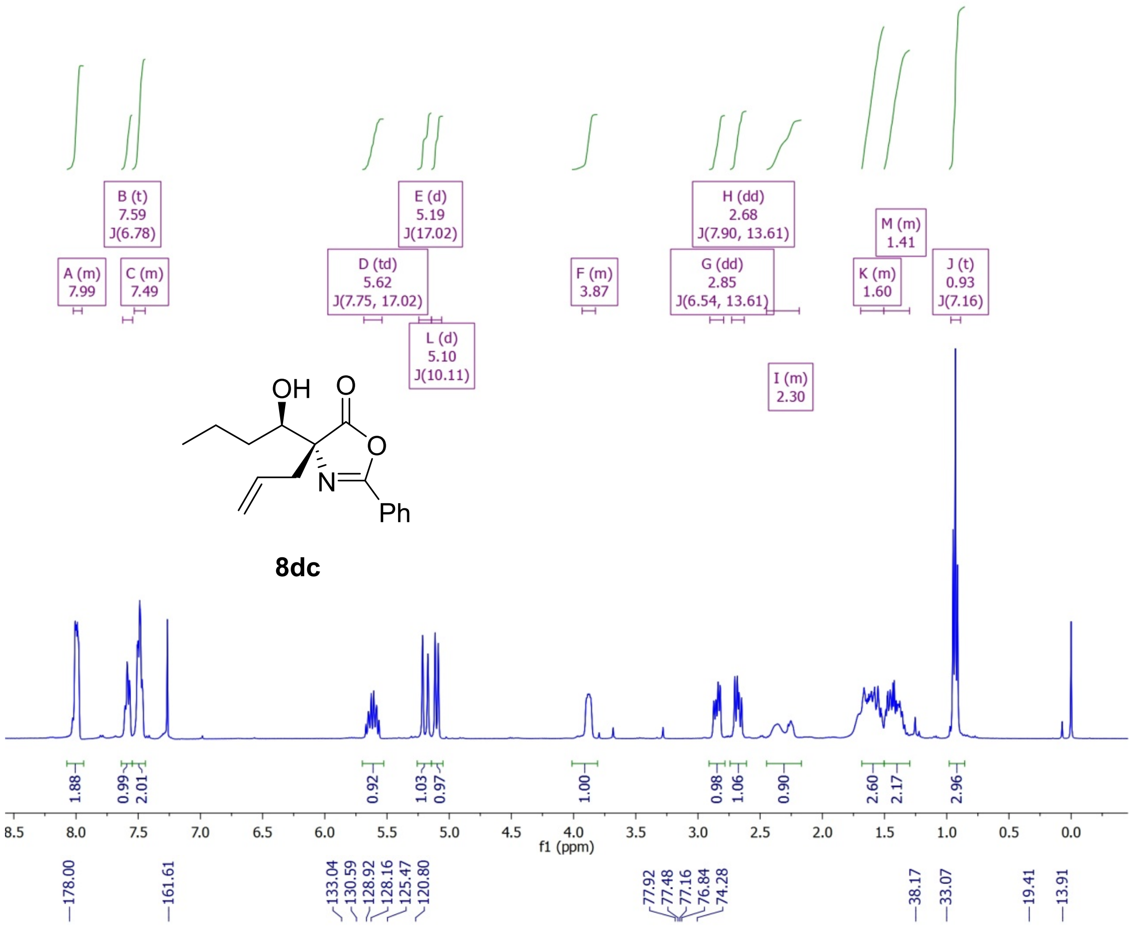
OH
O
O
N
Ph
8dc
A (m) 7.99
B (t) 7.59 J(6.78)
C (m) 7.49
D (td) 5.62 J(7.75, 17.02)
E (d) 5.19 J(17.02)
L (d) 5.10 J(10.11)
F (m) 3.87
G (dd) 2.85 J(6.54, 13.61)
H (dd) 2.68 J(7.90, 13.61)
I (m) 2.30
K (m) 1.60
M (m) 1.41
J (t) 0.93 J(7.16)
1.88
0.99
2.01
0.92
1.03
0.97
1.00
0.98
1.06
0.90
2.60
2.17
2.96
f1 (ppm)
178.00
161.61
133.04
130.59
128.92
128.16
125.47
120.80
77.92
77.48
77.16
76.84
74.28
38.17
33.07
19.41
13.91

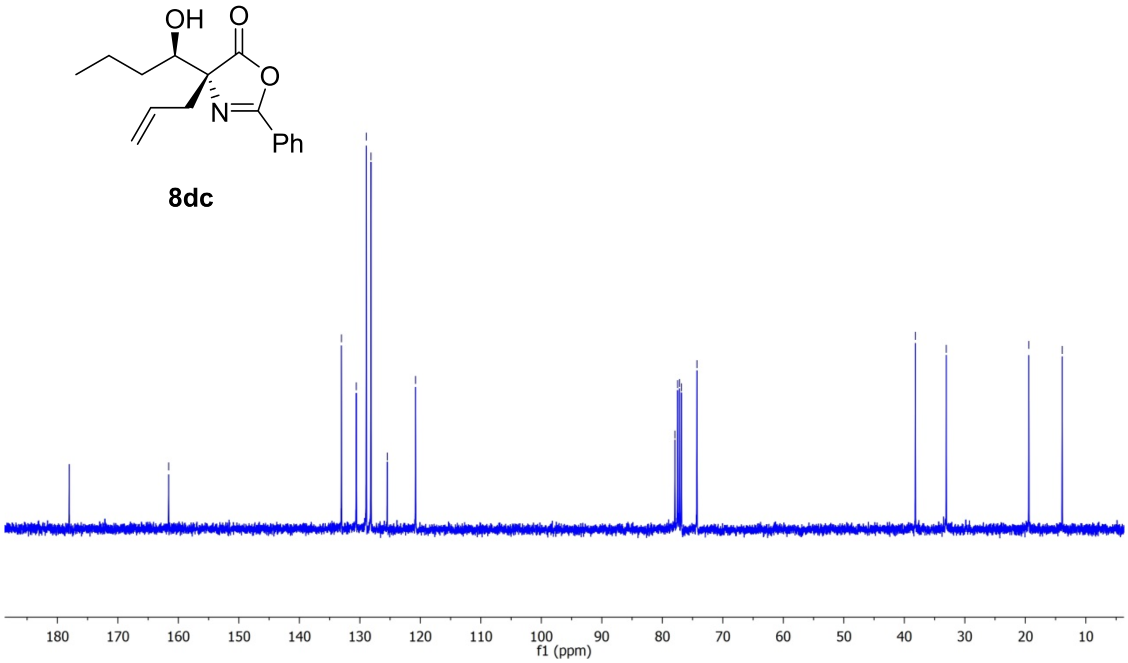
OH
O
O
N
Ph
8dc
f1 (ppm)

Data File C:\CHEM32\1\DATA\YZHENG\3-24RAC_1.D
Sample Name: 3-24rac

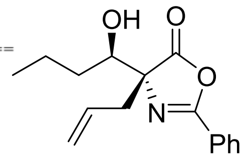

8dc
Racemic

===

Acq. Operator : yang
Acq. Instrument : Instrument 1 Location : -
Injection Date : 6/28/2011 10:49:47 PM
Acq. Method : C:\CHEM32\1\METHODS\METHOD-1_LC.M
Last changed : 4/2/2011 5:29:05 PM by jhl
Analysis Method : C:\CHEM32\1\METHODS\WASH.M
Last changed : 8/10/2011 11:23:50 AM by jhl
(modified after loading)
Sample Info : OJ-H, Hex:IPA = 97:3, 1 mL/min, 254 nm, 39 bar, Right

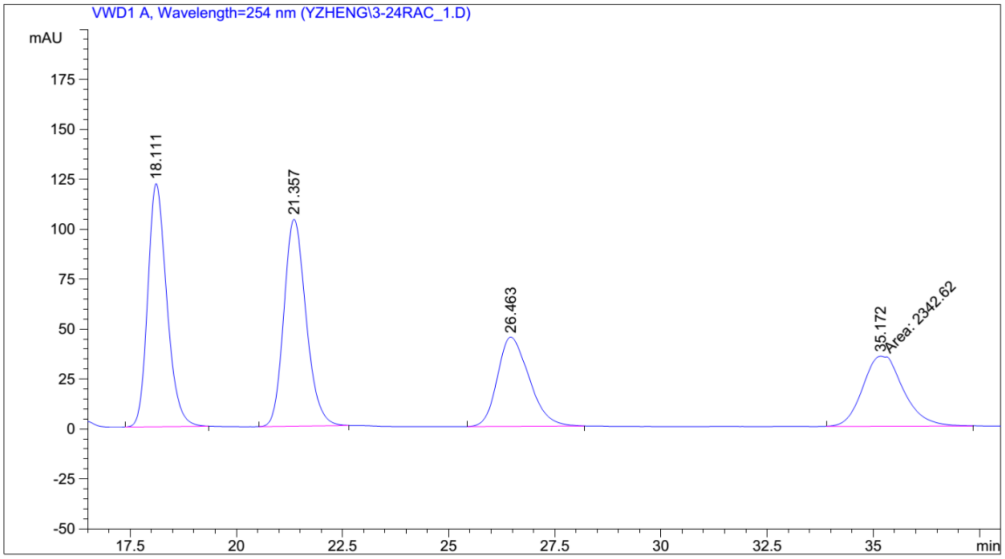

===

Area Percent Report

===

Sorted By : Signal
Multiplier : 1.0000
Dilution : 1.0000
Use Multiplier & Dilution Factor with ISTDs

Signal 1: VWD1 A, Wavelength=254 nm

Peak #	RetTime [min]	Type	Width [min]	Area mAU *s	Height [mAU]	Area %
1	18.111	BB	0.4802	3796.21582	121.75442	31.0119
2	21.357	BB	0.5592	3776.37964	103.59920	30.8499
3	26.463	BB	0.7773	2325.93457	44.70980	19.0010
4	35.172	MM	1.1125	2342.61719	35.09529	19.1372
Totals :				1.22411e4	305.15870	

===

*** End of Report ***

```
Data File C:\CHEM32\1\DATA\YZHENG\3-23_1.D
 Sample Name: 3-23

    =====================================================================
    Acq. Operator   : yang
    Acq. Instrument : Instrument 1                  Location : -
    Injection Date  : 6/28/2011 11:44:45 PM
    Acq. Method     : C:\CHEM32\1\METHODS\METHOD-1_LC.M
    Last changed    : 4/2/2011 5:29:05 PM by jhl
    Analysis Method : C:\CHEM32\1\METHODS\WASH.M
    Last changed    : 8/10/2011 11:22:00 AM by jhl
                      (modified after loading)
    Sample Info     : OJ-H, Hex:IPA = 97:3, 1 mL/min, 254 nm, 39 bar, Right
```

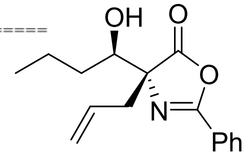

8dc

94% ee, *anti*/*syn* = 97.5/2.5

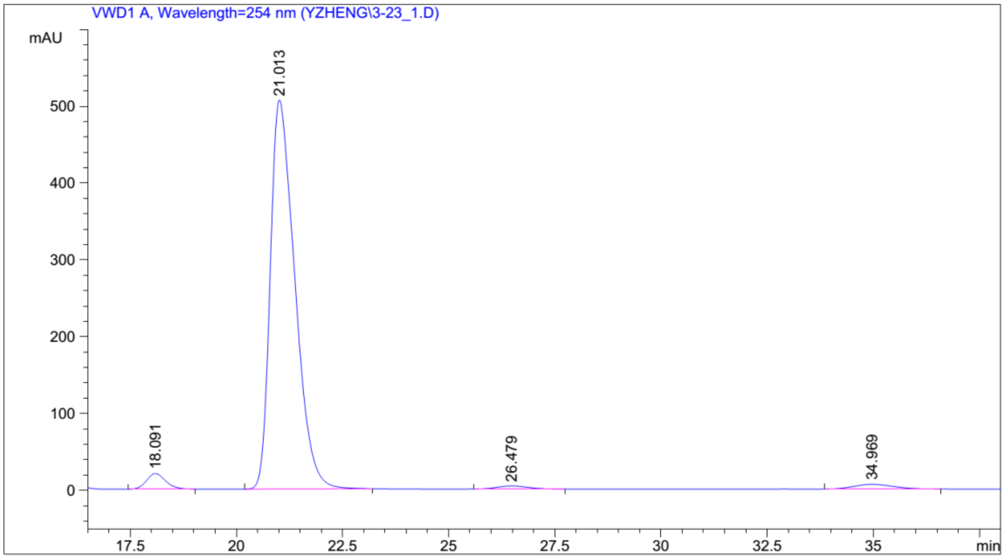


```
    =====================================================================
                             Area Percent Report
    =====================================================================

    Sorted By             :      Signal
    Multiplier            :      1.0000
    Dilution              :      1.0000
    Use Multiplier & Dilution Factor with ISTDs


    Signal 1: VWD1 A, Wavelength=254 nm
```

Peak #	RetTime [min]	Type	Width [min]	Area mAU *s	Height [mAU]	Area %
1	18.091	BB	0.4825	631.86621	20.30002	2.9376
2	21.013	BB	0.5856	2.02909e4	506.16669	94.3347
3	26.479	BB	0.7396	197.53523	3.95765	0.9184
4	34.969	BB	0.8847	389.17010	6.14300	1.8093

```
    Totals :                  2.15094e4   536.56736

    =====================================================================
                             *** End of Report ***
```

Instrument 1 8/10/2011 11:22:08 AM jhl   Page 1 of 1

Data File C:\CHEM32\1\DATA\YZHENG\3-96.D
Sample Name: 3-96

===

Acq. Operator : yang
Acq. Instrument : Instrument 1 Location : -
Injection Date : 9/14/2011 4:14:53 PM
Acq. Method : C:\CHEM32\1\METHODS\YANG.M
Last changed : 9/14/2011 3:30:28 PM by yang
Analysis Method : C:\CHEM32\1\METHODS\YANG.M
Last changed : 9/23/2011 3:13:05 PM by yang
(modified after loading)
Sample Info : OJ-H, Hex:IPA = 97:3, 1 mL/min, 254 nm, 39 bar, Right

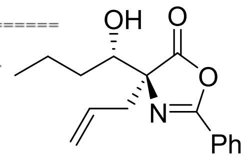

8dc-*ent*

-92% ee, *anti*/*syn* = 97/3

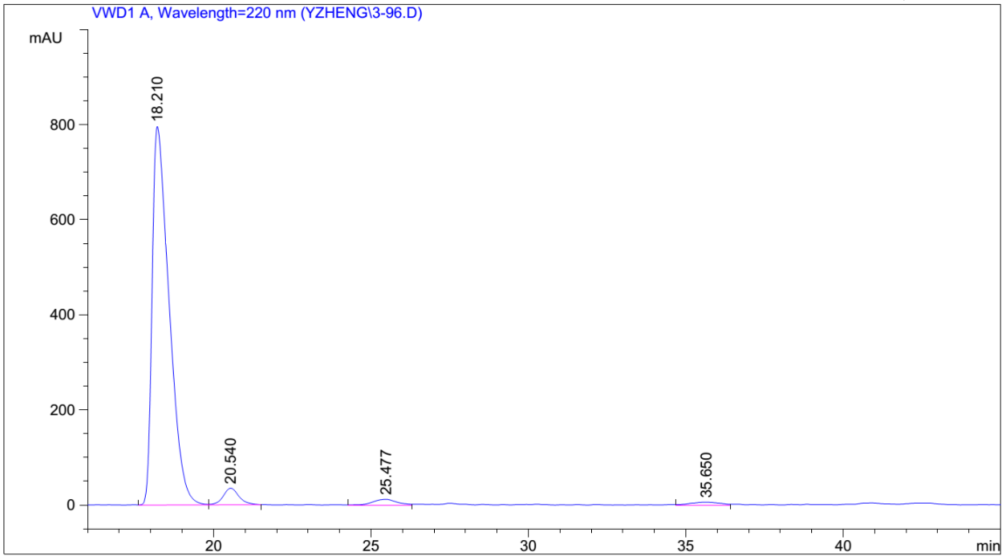

===

Area Percent Report

===

Sorted By : Signal
Multiplier : 1.0000
Dilution : 1.0000
Use Multiplier & Dilution Factor with ISTDs

Signal 1: VWD1 A, Wavelength=220 nm

Peak #	RetTime [min]	Type	Width [min]	Area mAU *s	Height [mAU]	Area %
1	18.210	VV	0.5513	2.97630e4	795.91199	92.9582
2	20.540	VB	0.5288	1218.60913	34.97573	3.8061
3	25.477	VV	0.8019	641.35376	11.95811	2.0031
4	35.650	VV	0.8521	394.65192	5.97526	1.2326

Totals : 3.20176e4 848.82110

===

*** End of Report ***

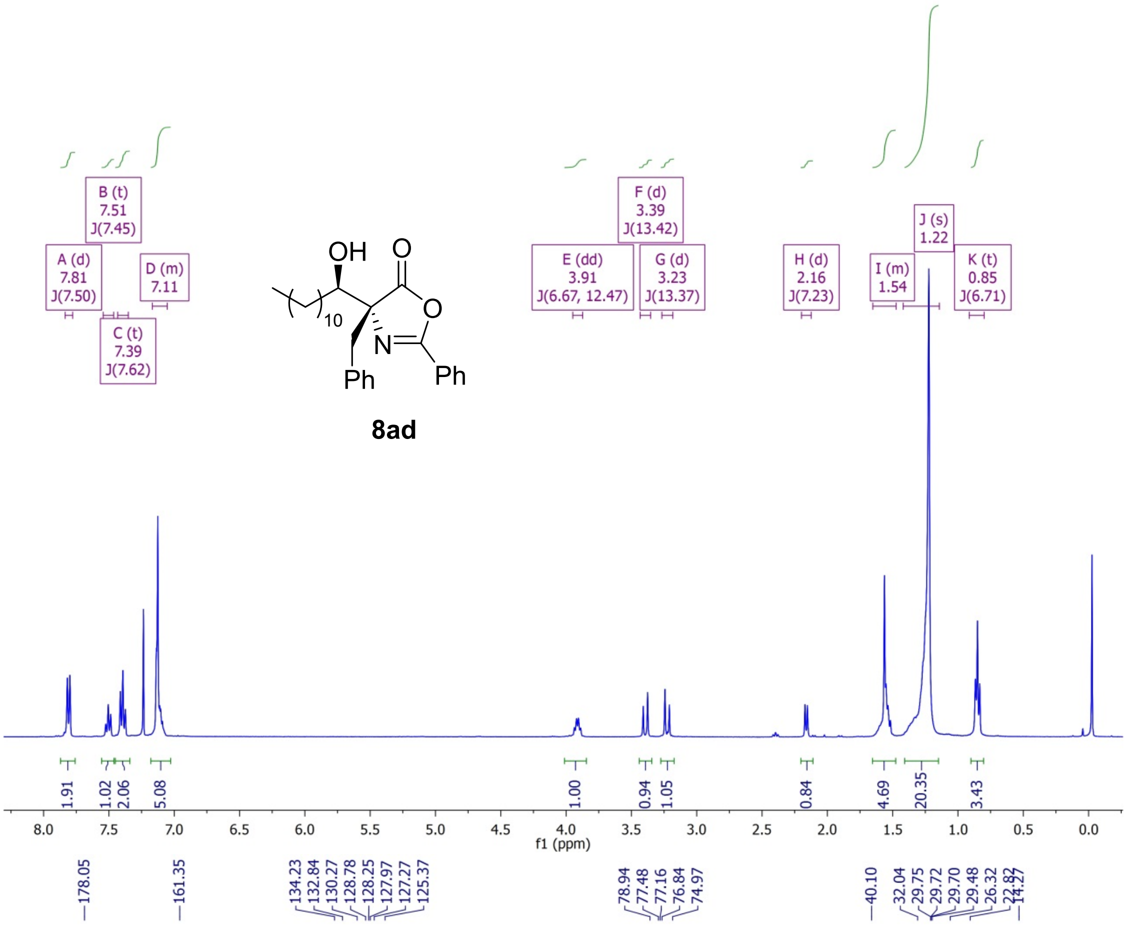
A (d) 7.81 J(7.50)
B (t) 7.51 J(7.45)
C (t) 7.39 J(7.62)
D (m) 7.11
E (dd) 3.91 J(6.67, 12.47)
F (d) 3.39 J(13.42)
G (d) 3.23 J(13.37)
H (d) 2.16 J(7.23)
I (m) 1.54
J (s) 1.22
K (t) 0.85 J(6.71)
OH
O
O
N
10
Ph
Ph
8ad
1.91
1.02
2.06
5.08
1.00
0.94
1.05
0.84
4.69
20.35
3.43
f1 (ppm)
178.05
161.35
134.23
132.84
130.27
128.78
128.25
127.97
127.27
125.37
78.94
77.48
77.16
76.84
74.97
40.10
32.04
29.75
29.72
29.70
29.48
26.32
22.82
14.27

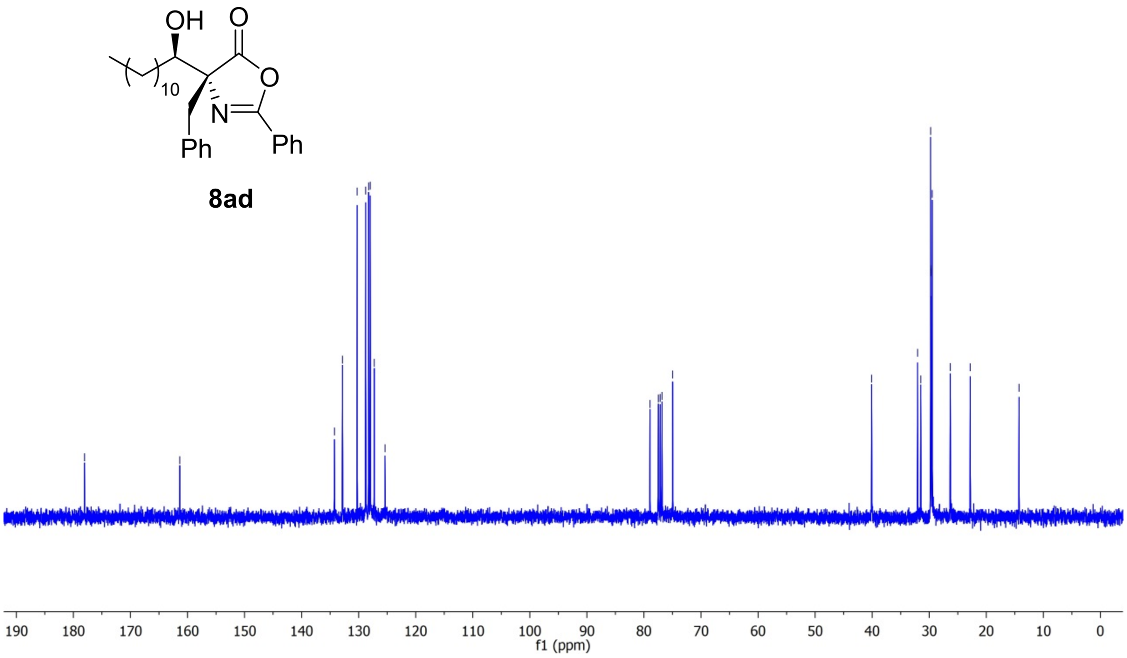
OH
O
O
N
10
Ph
Ph
8ad
f1 (ppm)

Data File C:\CHEM32\1\DATA\YZHENG\3-65RAC_3.D
Sample Name: 3-65rac

===

Acq. Operator : yang
Acq. Instrument : Instrument 1 Location : -
Injection Date : 8/3/2011 4:49:15 PM
Acq. Method : C:\CHEM32\1\METHODS\METHOD-1_LC.M
Last changed : 8/3/2011 4:22:01 PM by yang
(modified after loading)
Analysis Method : C:\CHEM32\1\METHODS\WASH.M
Last changed : 8/10/2011 11:17:35 AM by jhl
(modified after loading)
Sample Info : AS-H, Hex:IPA = 96.5:3.5, 1.0 mL/min, 254 nm, 44 bar, R
ight

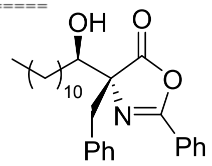

8ad
Racemic

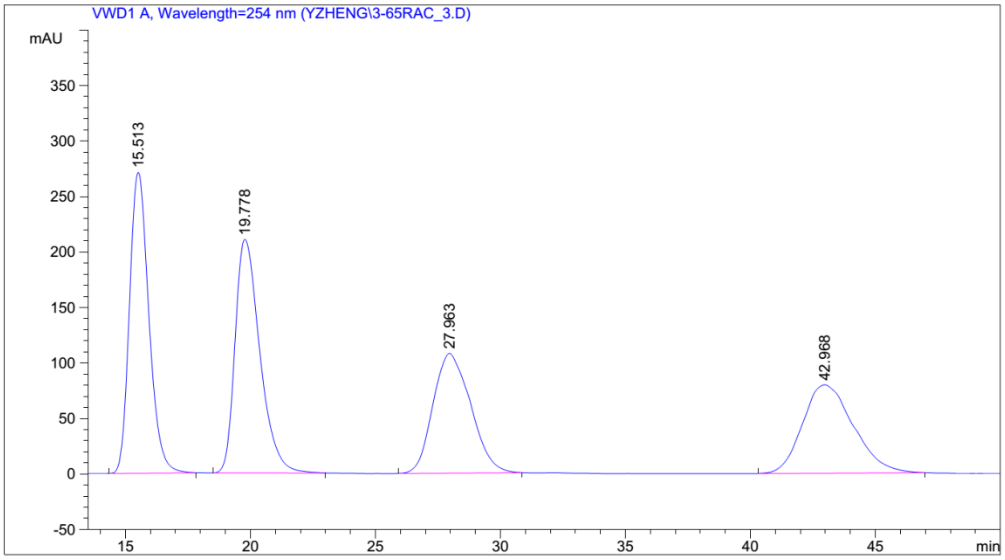

===
Area Percent Report
===

Sorted By : Signal
Multiplier : 1.0000
Dilution : 1.0000
Use Multiplier & Dilution Factor with ISTDs

Signal 1: VWD1 A, Wavelength=254 nm

Peak #	RetTime [min]	Type	Width [min]	Area mAU *s	Height [mAU]	Area %
1	15.513	BB	0.8414	1.45775e4	271.07748	28.2494
2	19.778	BB	1.0537	1.44936e4	210.49646	28.0867
3	27.963	BB	1.5207	1.12952e4	107.95037	21.8887
4	42.968	BB	1.9230	1.12367e4	79.62708	21.7752

Totals : 5.16030e4 669.15139

===
*** End of Report ***

Instrument 1 8/10/2011 11:17:40 AM jhl Page 1 of 1

Data File C:\CHEM32\1\DATA\YZHENG\3-64.D
Sample Name: 3-64

===

Acq. Operator : yang
Acq. Instrument : Instrument 1 Location : -
Injection Date : 8/3/2011 10:57:06 AM
Acq. Method : C:\CHEM32\1\METHODS\METHOD-1_LC.M
Last changed : 8/2/2011 5:34:32 PM by yang
(modified after loading)
Analysis Method : C:\CHEM32\1\METHODS\WASH.M
Last changed : 8/10/2011 11:16:24 AM by jhl
(modified after loading)
Sample Info : AS-H, Hex:IPA = 96.5:3.5, 1.0 mL/min, 254 nm, 44 bar, Right

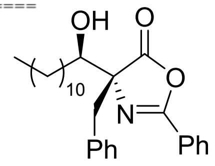

8ad

92% ee, *anti*/*syn* = 95.5/4.5

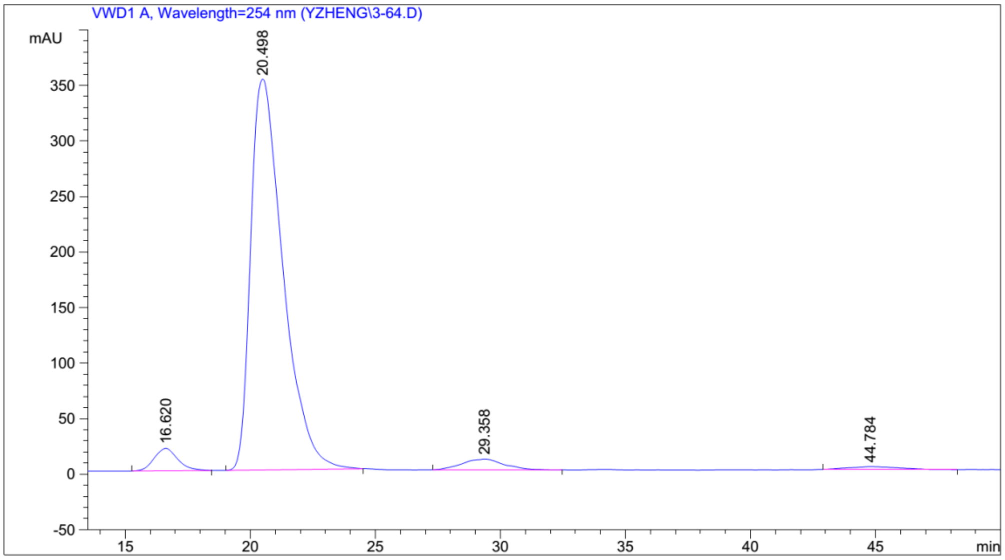

===
Area Percent Report
===

Sorted By : Signal
Multiplier : 1.0000
Dilution : 1.0000
Use Multiplier & Dilution Factor with ISTDs

Signal 1: VWD1 A, Wavelength=254 nm

Peak #	RetTime [min]	Type	Width [min]	Area mAU *s	Height [mAU]	Area %
1	16.620	BB	1.0042	1316.72168	20.18391	3.8245
2	20.498	BB	1.3238	3.15790e4	351.84460	91.7241
3	29.358	BB	1.5167	1124.23438	9.63799	3.2654
4	44.784	BB	1.7626	408.27173	2.79471	1.1859

Totals : 3.44282e4 384.46122

===
*** End of Report ***

Instrument 1 8/10/2011 11:16:28 AM jhl Page 1 of 1

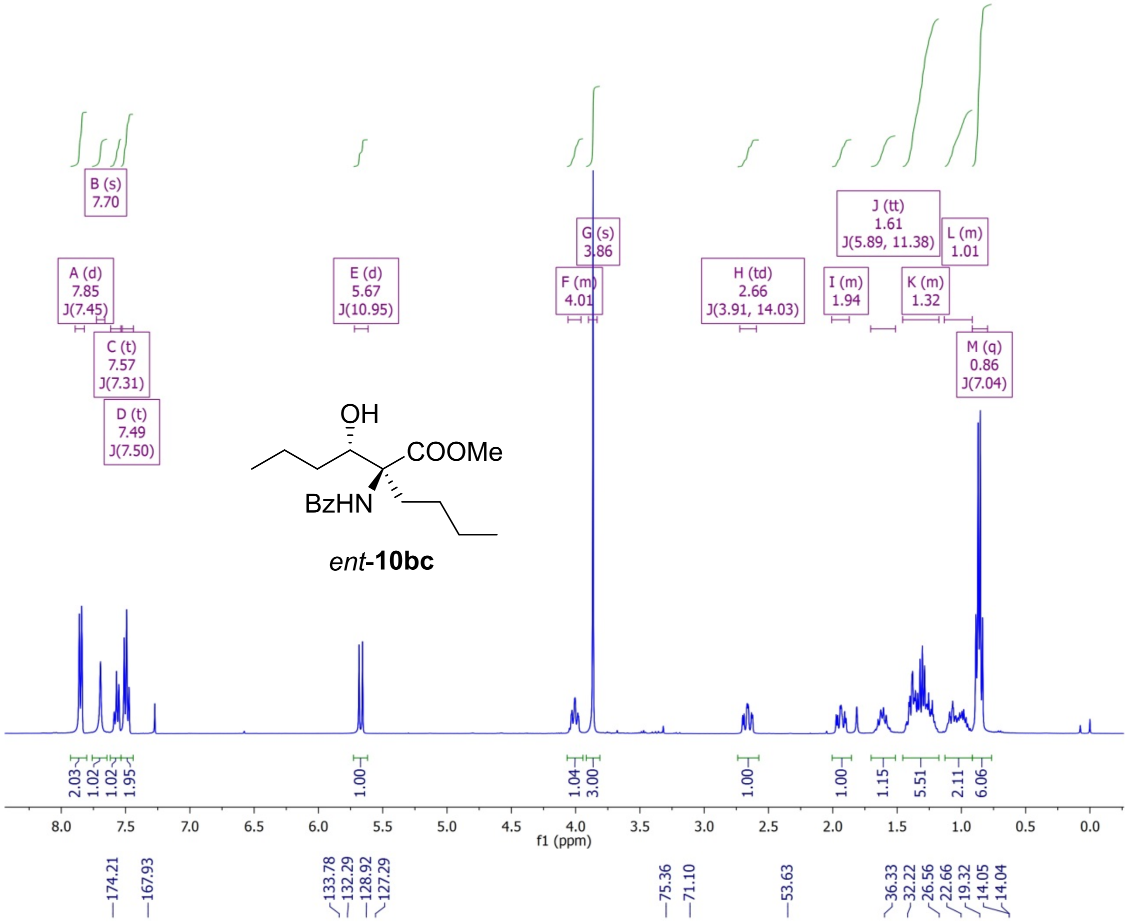
A (d)
7.85
J(7.45)
B (s)
7.70
C (t)
7.57
J(7.31)
D (t)
7.49
J(7.50)
E (d)
5.67
J(10.95)
F (m)
4.01
G (s)
3.86
H (td)
2.66
J(3.91, 14.03)
I (m)
1.94
J (tt)
1.61
J(5.89, 11.38)
K (m)
1.32
L (m)
1.01
M (q)
0.86
J(7.04)
OH
COOMe
BzHN
ent-10bc
2.03
1.02
1.02
1.95
1.00
1.04
3.00
1.00
1.00
1.15
5.51
2.11
6.06
f1 (ppm)
174.21
167.93
133.78
132.29
128.92
127.29
75.36
71.10
53.63
36.33
32.22
26.56
22.66
19.32
14.05
14.04

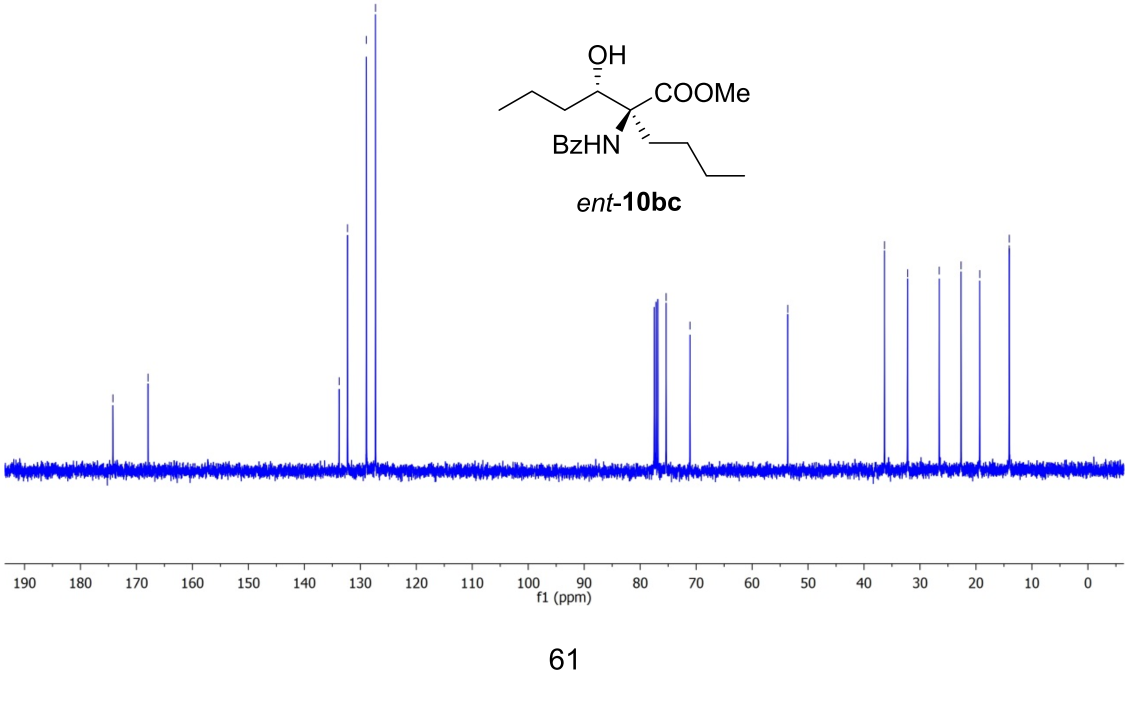
OH
COOMe
BzHN
ent-10bc
f1 (ppm)

Data File C:\CHEM32\1\DATA\YZHENG\3-135_1.D
Sample Name: 3-135rac

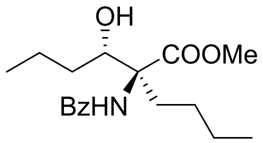

ent-**10bc**

Racemic

===

Acq. Operator : yang
Acq. Instrument : Instrument 1 Location : -
Injection Date : 10/20/2011 11:34:21 AM
Acq. Method : C:\CHEM32\1\METHODS\YANG.M
Last changed : 10/20/2011 11:08:32 AM by yang
(modified after loading)
Analysis Method : C:\CHEM32\1\METHODS\WASH.M
Last changed : 10/26/2011 10:41:01 AM by jhl
(modified after loading)
Sample Info : AD-H, Hex:IPA = 90:10, 1.0 mL/min, 254 nm, 42 bar, Righ
t

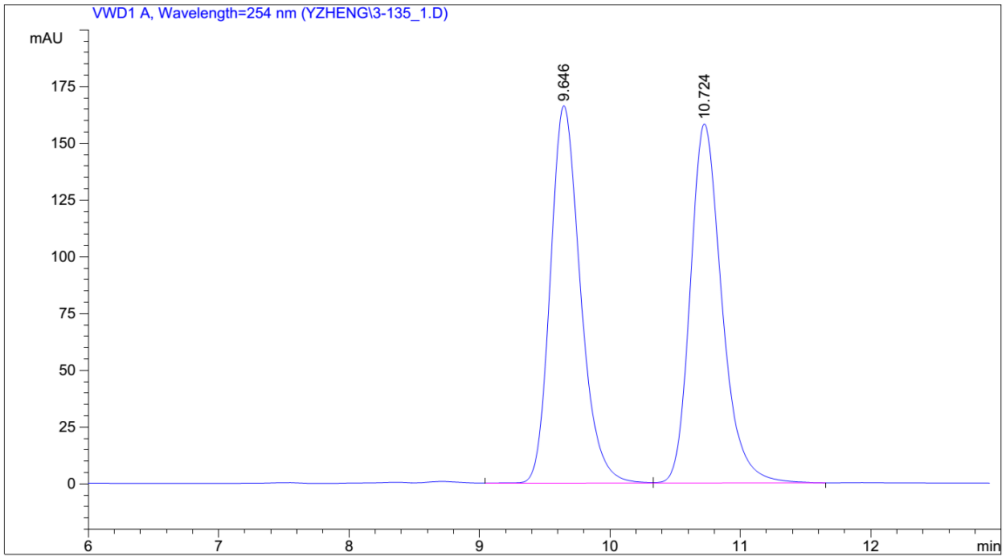

===

Area Percent Report

===

Sorted By : Signal
Multiplier : 1.0000
Dilution : 1.0000
Use Multiplier & Dilution Factor with ISTDs

Signal 1: VWD1 A, Wavelength=254 nm

Peak #	RetTime [min]	Type	Width [min]	Area mAU *s	Height [mAU]	Area %
1	9.646	VV	0.2451	2663.87866	166.34001	50.0445
2	10.724	VB	0.2572	2659.14600	158.23003	49.9555

Totals : 5323.02466 324.57004

===

*** End of Report ***

Instrument 1 10/26/2011 10:41:14 AM jhl Page 1 of 1

Data File C:\CHEM32\1\DATA\YZHENG\3-128_1.D
Sample Name: 3-128

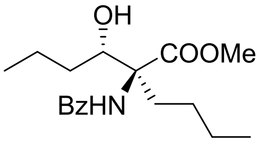

ent-**10bc**

-92% ee

===

Acq. Operator : yang
Acq. Instrument : Instrument 1 Location : -
Injection Date : 10/20/2011 12:12:47 PM
Acq. Method : C:\CHEM32\1\METHODS\YANG.M
Last changed : 10/20/2011 11:08:32 AM by yang
(modified after loading)
Analysis Method : C:\CHEM32\1\METHODS\WASH.M
Last changed : 10/26/2011 10:39:21 AM by jhl
(modified after loading)
Sample Info : AD-H, Hex:IPA = 90:10, 1.0 mL/min, 254 nm, 42 bar, Righ
t

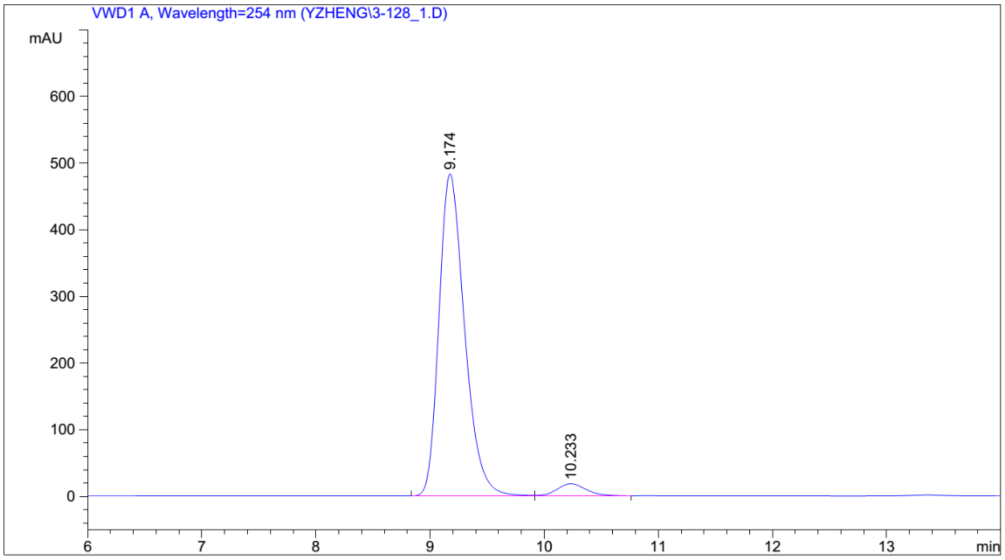

===
Area Percent Report
===

Sorted By : Signal
Multiplier : 1.0000
Dilution : 1.0000
Use Multiplier & Dilution Factor with ISTDs

Signal 1: VWD1 A, Wavelength=254 nm

Peak #	RetTime [min]	Type	Width [min]	Area mAU *s	Height [mAU]	Area %
1	9.174	BV	0.2400	7529.78955	483.25226	95.9212
2	10.233	VB	0.2699	320.18448	18.27895	4.0788

Totals : 7849.97403 501.53121

===
*** End of Report ***

Instrument 1 10/26/2011 10:39:44 AM jhl Page 1 of 1

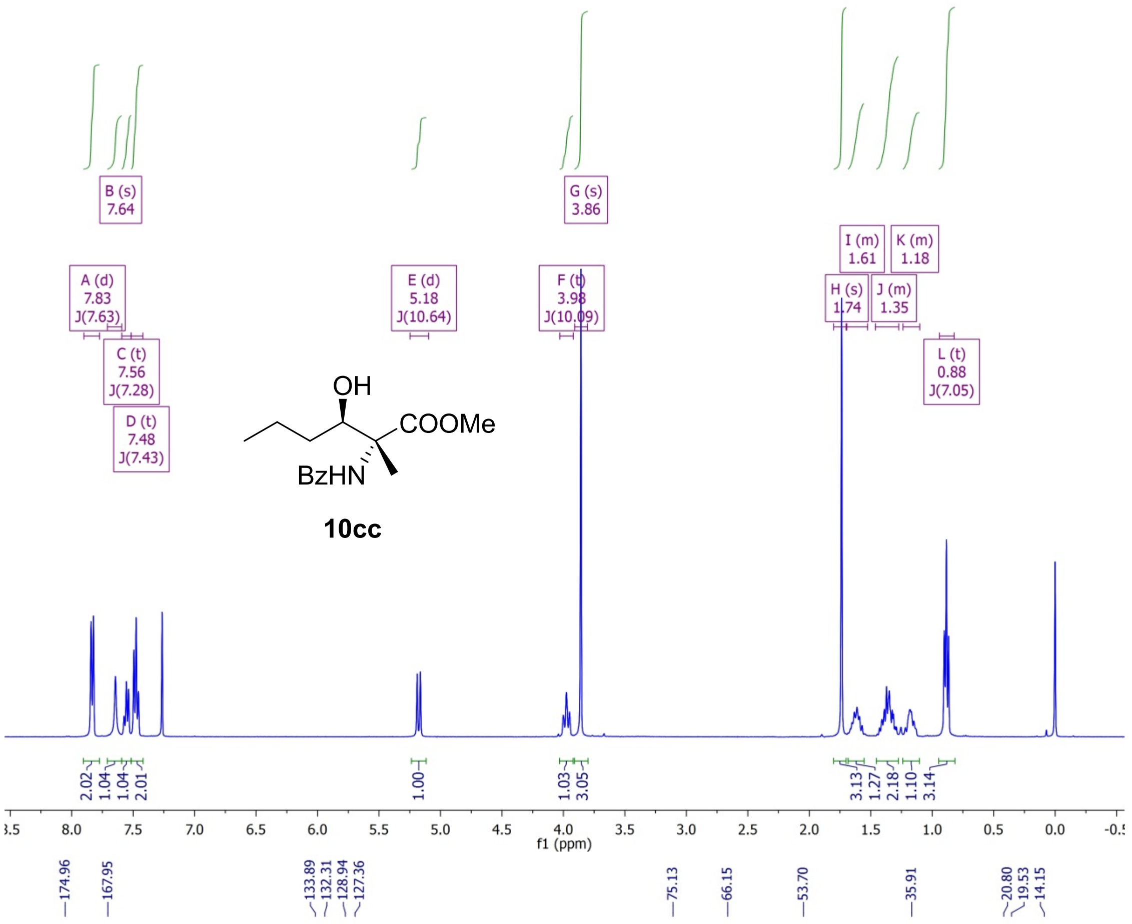

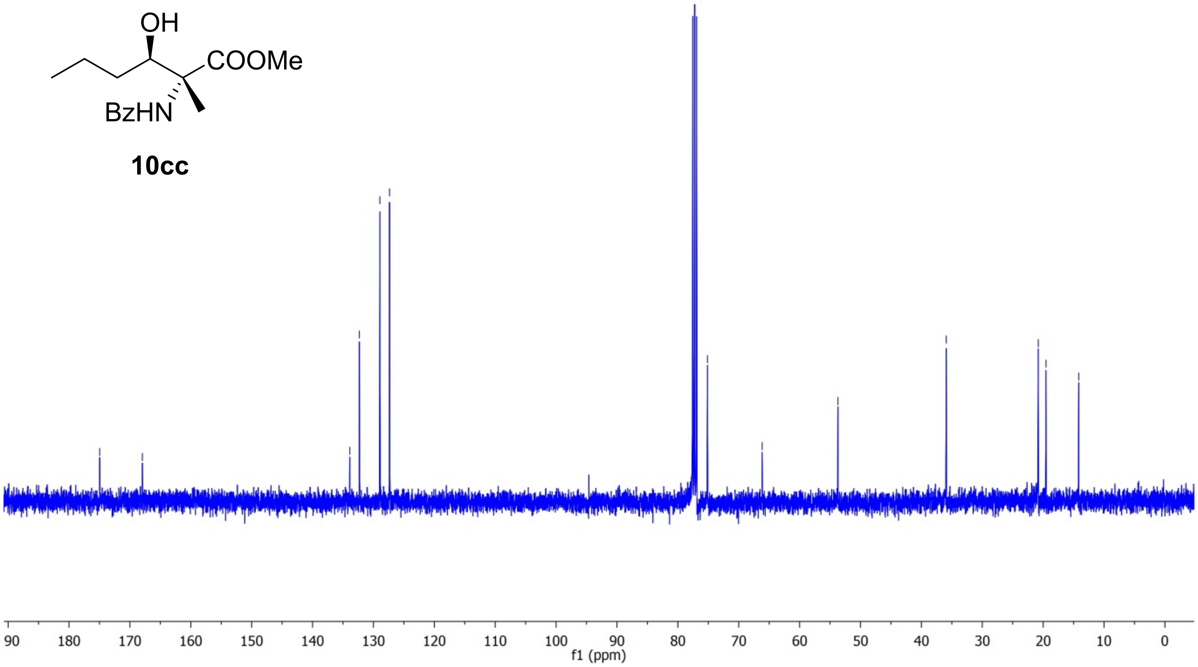

Data File C:\CHEM32\1\DATA\YZHENG\3-150U_1.D
Sample Name: 3-150U

===
Acq. Operator   : yang
Acq. Instrument : Instrument 1   Location : -
Injection Date : 2/22/2012 10:59:30 AM
Acq. Method   : C:\CHEM32\1\METHODS\YANG.M
Last changed   : 2/22/2012 10:21:20 AM by yang
                  (modified after loading)
Analysis Method : C:\CHEM32\1\METHODS\YANG.M
Last changed   : 2/22/2012 12:36:15 PM by yang
                  (modified after loading)
Sample Info   : AD-H, Hex:IPA = 90:10, 1.0 mL/min, 254 nm, 41 bar, Righ
                  t

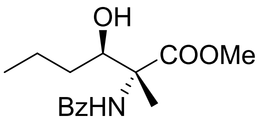

10cc

Racemic

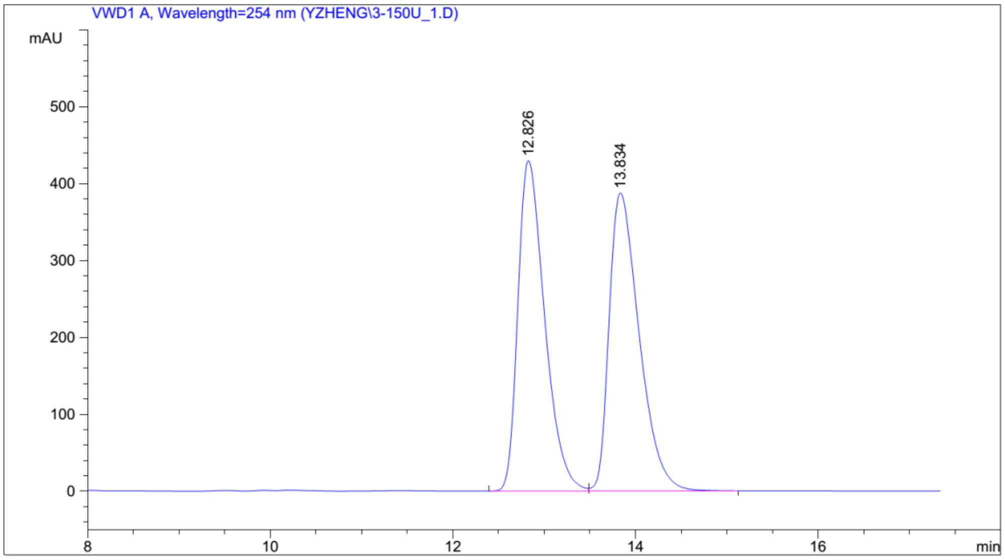

===
Area Percent Report
===

Sorted By   :   Signal
Multiplier   :   1.0000
Dilution   :   1.0000
Use Multiplier & Dilution Factor with ISTDs

Signal 1: VWD1 A, Wavelength=254 nm

Peak #	RetTime [min]	Type	Width [min]	Area mAU *s	Height [mAU]	Area %
1	12.826	BV	0.3132	8744.77539	429.55450	49.7204
2	13.834	VB	0.3506	8843.12598	387.57666	50.2796

Totals :   1.75879e4   817.13116

===
*** End of Report ***

Data File C:\CHEM32\1\DATA\YZHENG\3-153_1.D
Sample Name: 3-153

===

Acq. Operator : yang
Acq. Instrument : Instrument 1 Location : -
Injection Date : 2/22/2012 11:19:36 AM
Acq. Method : C:\CHEM32\1\METHODS\YANG.M
Last changed : 2/22/2012 10:21:20 AM by yang
(modified after loading)
Analysis Method : C:\CHEM32\1\METHODS\YANG.M
Last changed : 2/22/2012 12:35:10 PM by yang
(modified after loading)
Sample Info : AD-H, Hex:IPA = 90:10, 1.0 mL/min, 254 nm, 41 bar, Righ
t

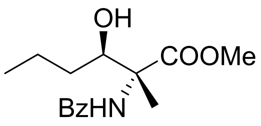

10cc

95% ee

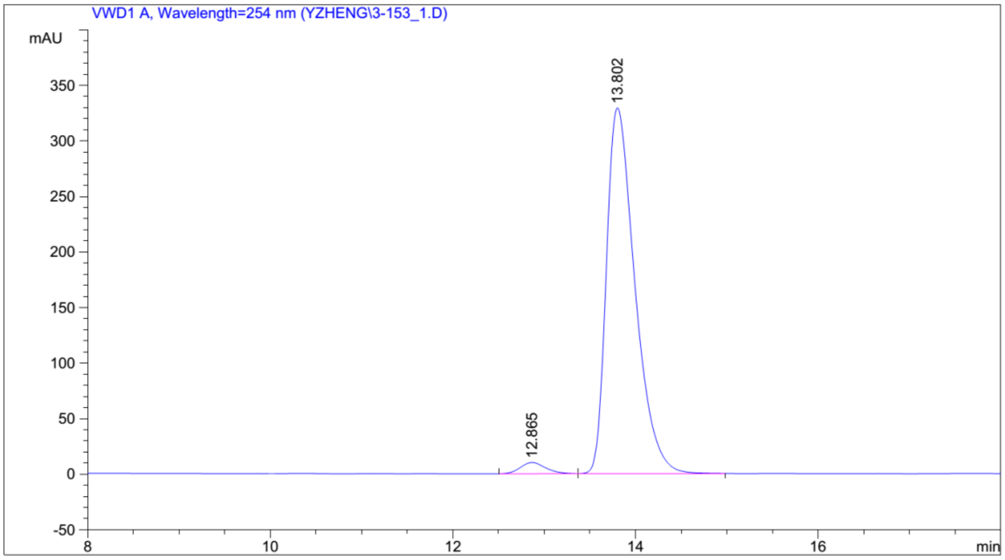

===

Area Percent Report

===

Sorted By : Signal
Multiplier : 1.0000
Dilution : 1.0000
Use Multiplier & Dilution Factor with ISTDs

Signal 1: VWD1 A, Wavelength=254 nm

Peak #	RetTime [min]	Type	Width [min]	Area mAU *s	Height [mAU]	Area %
1	12.865	BV	0.2930	194.25142	10.15565	2.6504
2	13.802	VB	0.3328	7134.79004	329.33212	97.3496

Totals : 7329.04146 339.48777

===

*** End of Report ***

Instrument 1 2/22/2012 12:35:38 PM yang Page 1 of 1

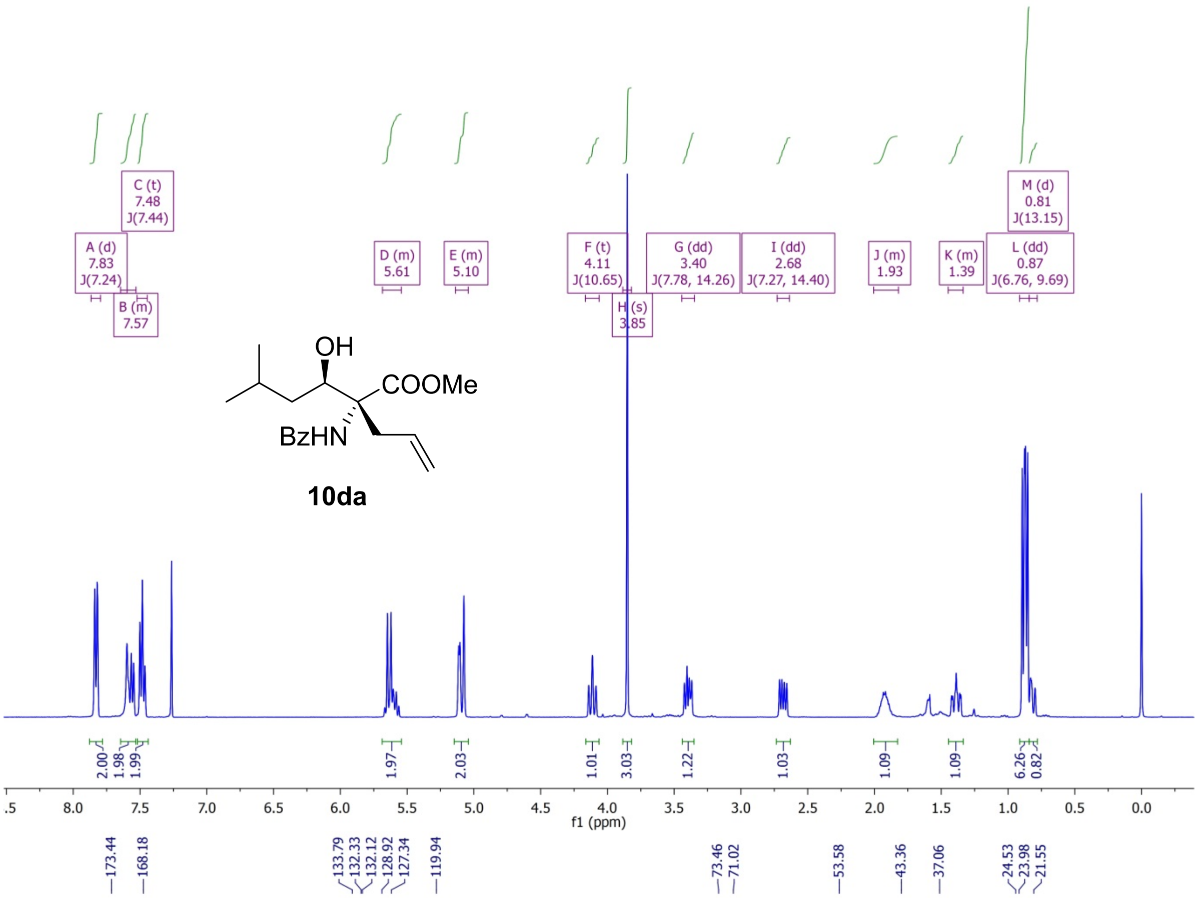

A (d) 7.83 J(7.24)
B (m) 7.57
C (t) 7.48 J(7.44)
D (m) 5.61
E (m) 5.10
F (t) 4.11 J(10.65)
H (s) 3.85
G (dd) 3.40 J(7.78, 14.26)
I (dd) 2.68 J(7.27, 14.40)
J (m) 1.93
K (m) 1.39
L (dd) 0.87 J(6.76, 9.69)
M (d) 0.81 J(13.15)
OH
COOMe
BzHN
10da
2.00 1.98 1.99 1.97 2.03 1.01 3.03 1.22 1.03 1.09 1.09 6.26 0.82
f1 (ppm)
173.44 168.18 133.79 132.33 132.12 128.92 127.34 119.94 73.46 71.02 53.58 43.36 37.06 24.53 23.98 21.55

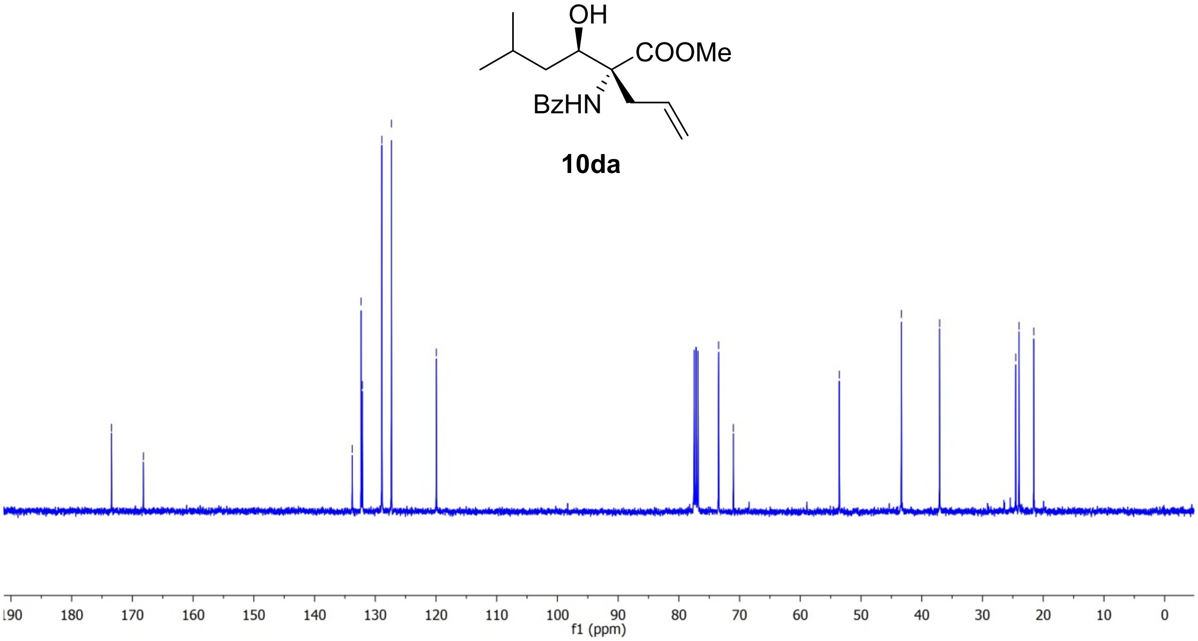

OH
COOMe
BzHN
10da
f1 (ppm)

Data File C:\CHEM32\1\DATA\YZHENG\4-22U_1.D
Sample Name: 4-22U

===

Acq. Operator : yang
Acq. Instrument : Instrument 1 Location : -
Injection Date : 2/21/2012 4:31:25 PM
Acq. Method : C:\CHEM32\1\METHODS\YANG.M
Last changed : 2/21/2012 3:23:10 PM by jhl
(modified after loading)
Analysis Method : C:\CHEM32\1\METHODS\YANG.M
Last changed : 2/22/2012 12:37:27 PM by yang
(modified after loading)
Sample Info : AD-H, Hex:IPA = 90:10, 1.0 mL/min, 254 nm, 41 bar, Right

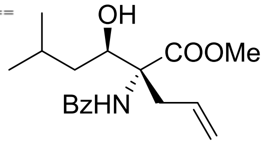

10da

Racemic

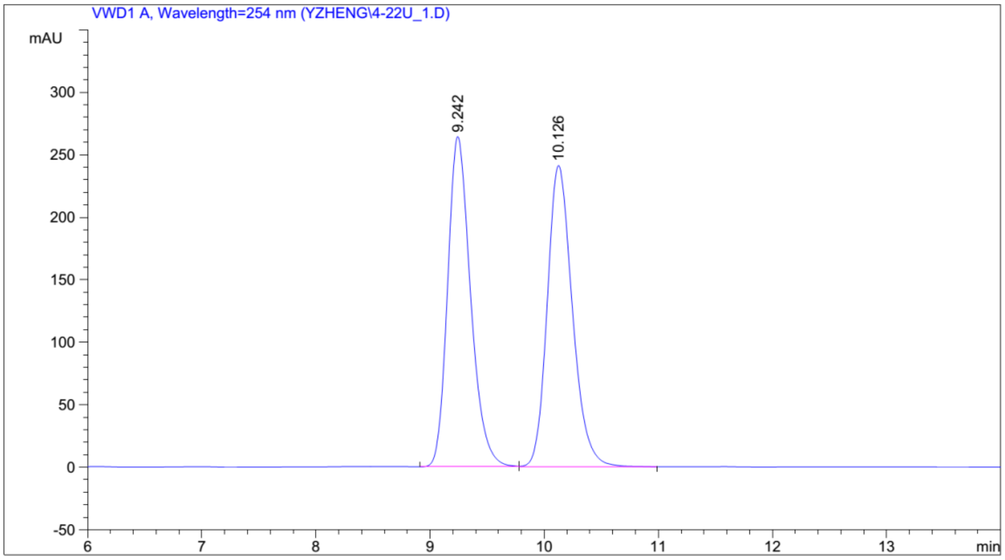

===

Area Percent Report

===

Sorted By : Signal
Multiplier : 1.0000
Dilution : 1.0000
Use Multiplier & Dilution Factor with ISTDs

Signal 1: VWD1 A, Wavelength=254 nm

Peak #	RetTime [min]	Type	Width [min]	Area mAU *s	Height [mAU]	Area %
1	9.242	VV	0.2142	3697.71021	264.33917	50.0371
2	10.126	VB	0.2354	3692.22241	241.17551	49.9629

Totals : 7389.93262 505.51468

===

*** End of Report ***

Data File C:\CHEM32\1\DATA\YZHENG\4-21_1.D
Sample Name: 4-22U

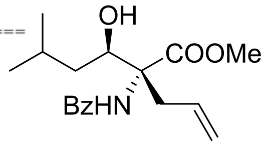

10da

95% ee

```
=====================================================================
Acq. Operator   : yang
Acq. Instrument : Instrument 1                    Location :  -
Injection Date  : 2/21/2012 4:48:08 PM
Acq. Method     : C:\CHEM32\1\METHODS\YANG.M
Last changed    : 2/21/2012 3:23:10 PM by jhl
                  (modified after loading)
Analysis Method : C:\CHEM32\1\METHODS\YANG.M
Last changed    : 2/22/2012 12:38:09 PM by yang
                  (modified after loading)
Sample Info     : AD-H, Hex:IPA = 90:10, 1.0 mL/min, 254 nm, 41 bar, Righ
                  t
```

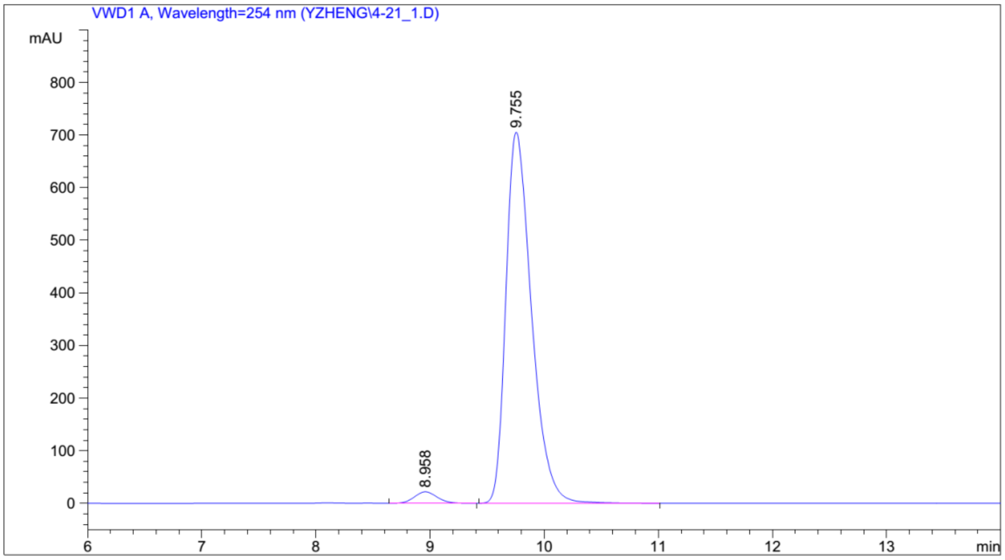


```
=====================================================================
                         Area Percent Report
=====================================================================

Sorted By             :      Signal
Multiplier            :      1.0000
Dilution              :      1.0000
Use Multiplier & Dilution Factor with ISTDs


Signal 1: VWD1 A, Wavelength=254 nm

Peak RetTime Type  Width     Area      Height     Area
  #   [min]        [min]  mAU   *s    [mAU   ]     %
----|-------|----|-------|----------|----------|--------|
   1   8.958 VB    0.2151  306.92242   22.00724   2.7158
   2   9.755 BB    0.2401 1.09945e4  705.41644  97.2842

Totals :                  1.13014e4  727.42368

=====================================================================
                         *** End of Report ***
```

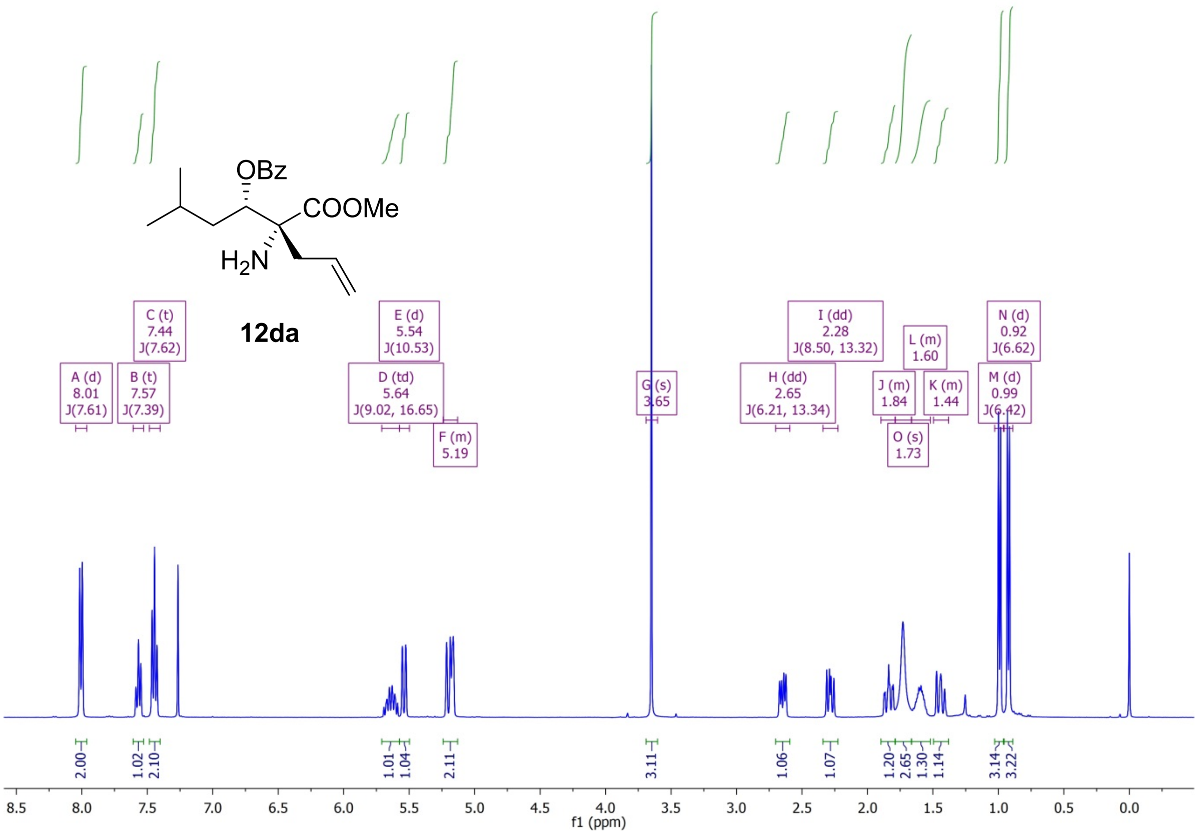
OBz
COOMe
H2N
12da
A (d) 8.01 J(7.61)
B (t) 7.57 J(7.39)
C (t) 7.44 J(7.62)
D (td) 5.64 J(9.02, 16.65)
E (d) 5.54 J(10.53)
F (m) 5.19
G (s) 3.65
H (dd) 2.65 J(6.21, 13.34)
I (dd) 2.28 J(8.50, 13.32)
J (m) 1.84
O (s) 1.73
L (m) 1.60
K (m) 1.44
M (d) 0.99 J(6.42)
N (d) 0.92 J(6.62)
2.00
1.02
2.10
1.01
1.04
2.11
3.11
1.06
1.07
1.20
2.65
1.30
1.14
3.14
3.22
f1 (ppm)

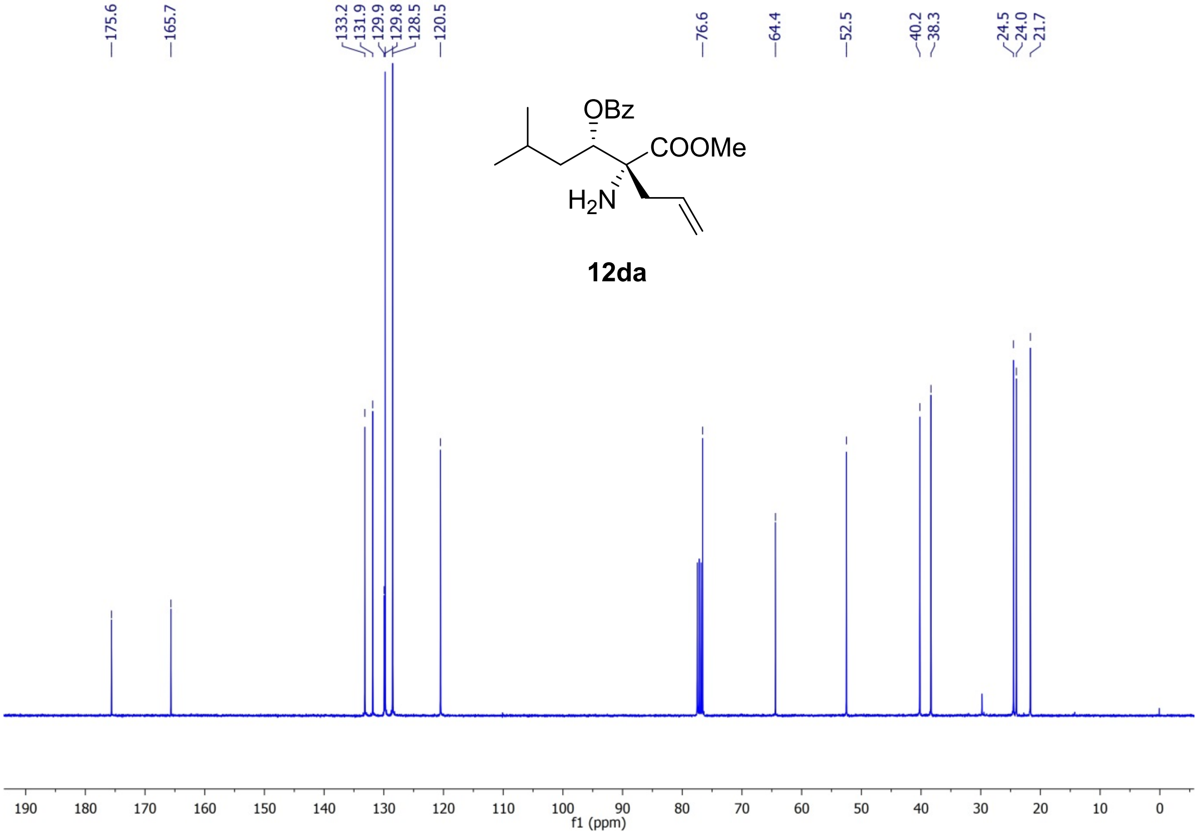
175.6
165.7
133.2
131.9
129.9
129.8
128.5
120.5
76.6
64.4
52.5
40.2
38.3
24.5
24.0
21.7
OBz
COOMe
H2N
12da
f1 (ppm)

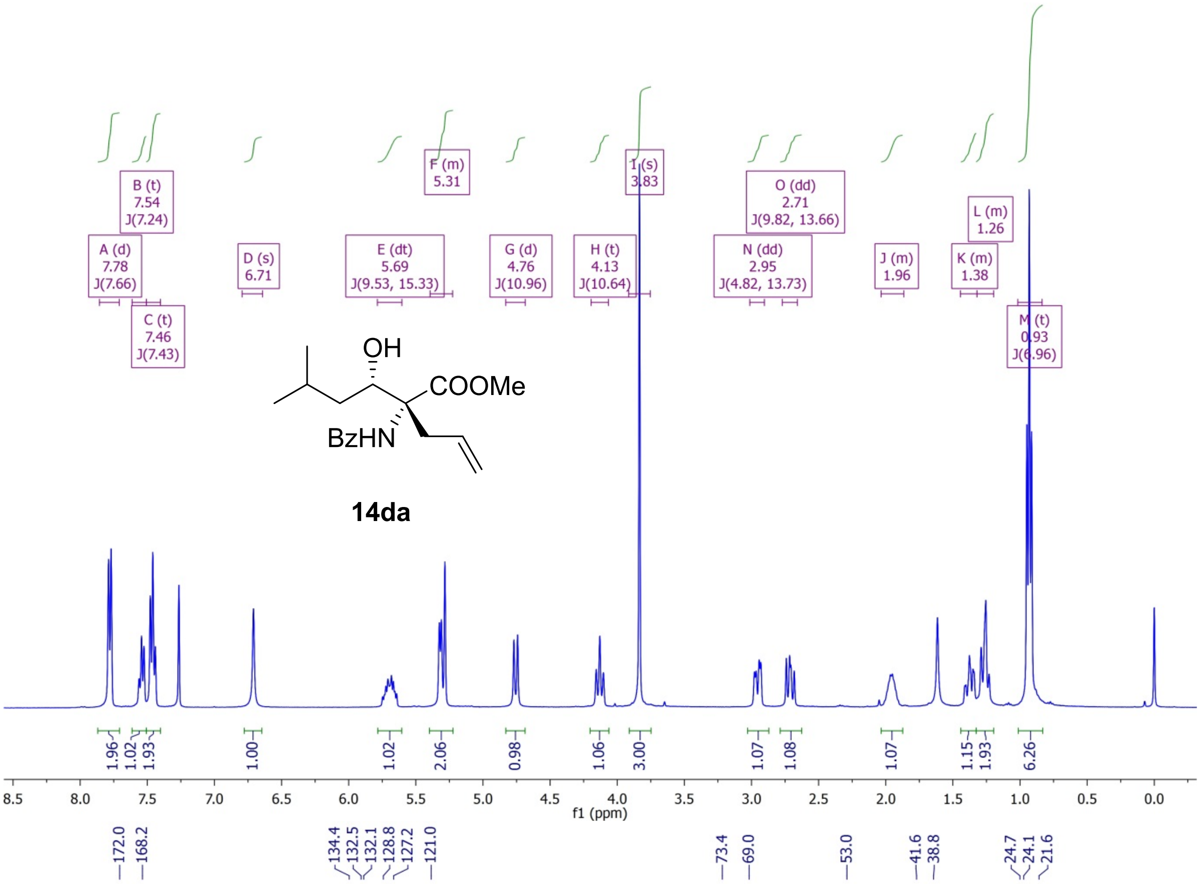

A (d) 7.78 J(7.66)
B (t) 7.54 J(7.24)
C (t) 7.46 J(7.43)
D (s) 6.71
E (dt) 5.69 J(9.53, 15.33)
F (m) 5.31
G (d) 4.76 J(10.96)
H (t) 4.13 J(10.64)
I (s) 3.83
N (dd) 2.95 J(4.82, 13.73)
O (dd) 2.71 J(9.82, 13.66)
J (m) 1.96
K (m) 1.38
L (m) 1.26
M (t) 0.93 J(6.96)
OH
COOMe
BzHN
14da
1.96 1.02 1.93 1.00 1.02 2.06 0.98 1.06 3.00 1.07 1.08 1.07 1.15 1.93 6.26
f1 (ppm)
172.0 168.2 134.4 132.5 132.1 128.8 127.2 121.0 73.4 69.0 53.0 41.6 38.8 24.7 24.1 21.6

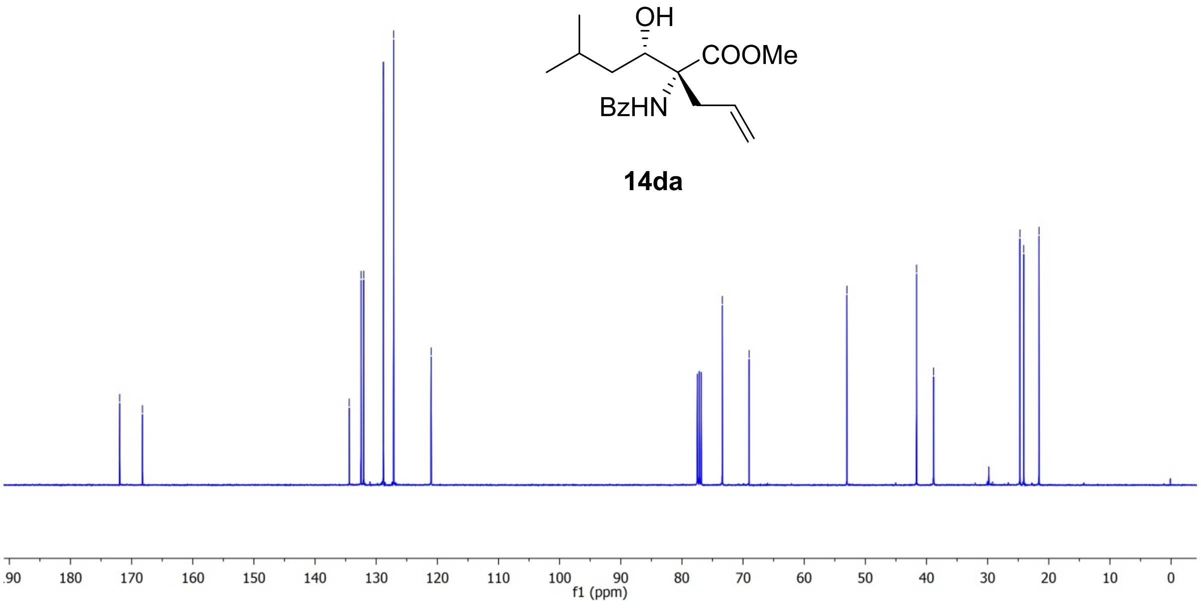

OH
COOMe
BzHN
14da
f1 (ppm)

Data File C:\HPCHEM\1\DATA\YZHENG\4-39.D
Sample Name: 4-39

===

Acq. Operator : YANG
Acq. Instrument : Deng Lab LC Location : Vial 21
Injection Date : 10/3/2014 3:36:36 PM
Inj Volume : 20 µl
Acq. Method : C:\HPCHEM\1\METHODS\METHOD 1.M
Last changed : 10/3/2014 3:25:09 PM by YANG
(modified after loading)
Analysis Method : C:\HPCHEM\1\METHODS\METHOD 1.M
Last changed : 10/4/2014 3:59:26 PM by CHAO
(modified after loading)
Sample Info : AD-H, Hex/IPA=90/10, 1.0 mL/min, 254nm, 47 bar, 25 C, 1
eft

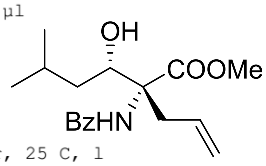

14da, 95% ee

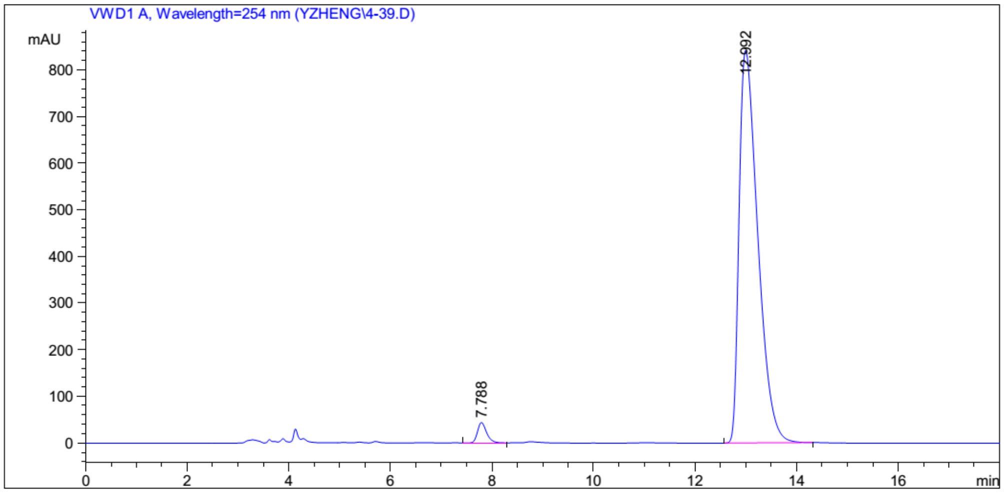

===
Fraction Information
===
Fraction collection off
===
No Fractions found.
===
===
Area Percent Report
===

Sorted By : Signal
Multiplier : 1.0000
Dilution : 1.0000
Use Multiplier & Dilution Factor with ISTDs

Signal 1: VWD1 A, Wavelength=254 nm

Peak #	RetTime [min]	Type	Width [min]	Area mAU *s	Height [mAU]	Area %
1	7.788	VV	0.1921	560.22424	44.05143	2.5360
2	12.992	BB	0.3944	2.15309e4	842.17053	97.4640

Totals : 2.20912e4 886.22196

Data File C:\HPCHEM\1\DATA\YZHENG\4-54.D
Sample Name: 4-54

===

Acq. Operator : YANG
Acq. Instrument : Deng Lab LC Location : Vial 21
Injection Date : 10/4/2014 4:20:21 PM
Inj Volume : 20 µl
Acq. Method : C:\HPCHEM\1\METHODS\METHOD 1.M
Last changed : 10/4/2014 4:02:24 PM by YANG
(modified after loading)
Analysis Method : C:\HPCHEM\1\METHODS\METHOD 1.M
Last changed : 10/4/2014 4:38:43 PM by CHAO
(modified after loading)
Sample Info : AD-H, Hex/IPA=90/10, 1.0 mL/min, 254 nm, 47 bar, 25 C, left

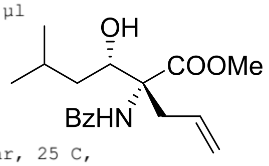

14da, Racemic

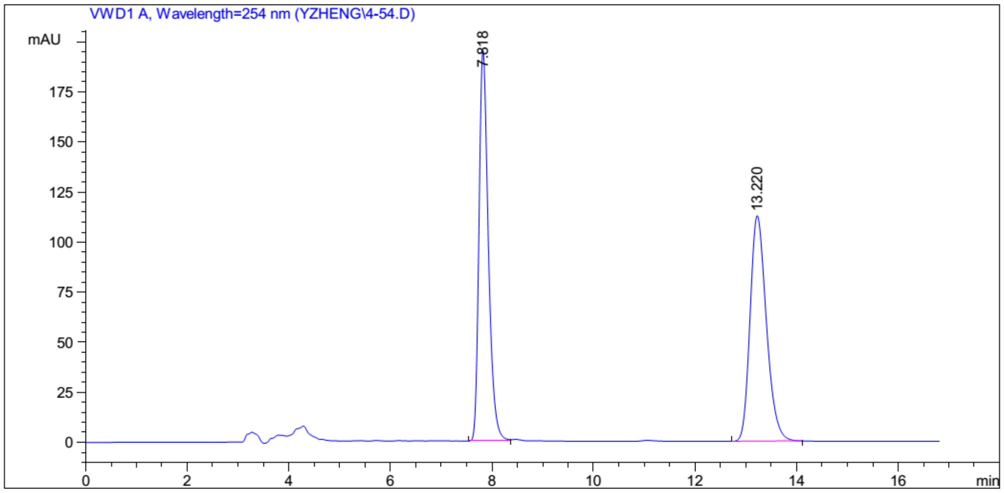

===

Fraction Information

===

Fraction collection off

===

No Fractions found.

===

===

Area Percent Report

===

Sorted By : Signal
Multiplier : 1.0000
Dilution : 1.0000
Use Multiplier & Dilution Factor with ISTDs

Signal 1: VWD1 A, Wavelength=254 nm

Peak #	RetTime [min]	Type	Width [min]	Area mAU *s	Height [mAU]	Area %
1	7.818	BV	0.1944	2497.13208	195.17357	49.8004
2	13.220	BB	0.3436	2517.15137	112.67712	50.1996

Totals : 5014.28345 307.85069

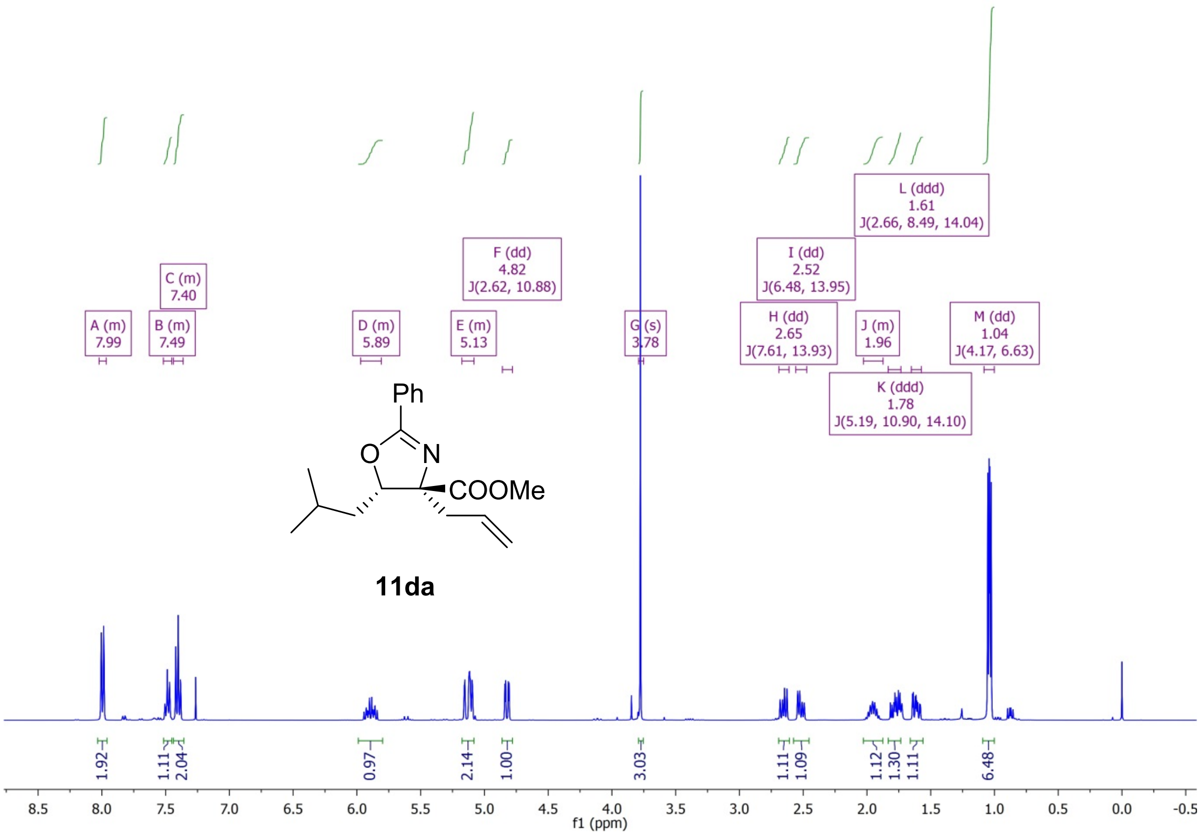
A (m)
7.99
B (m)
7.49
C (m)
7.40
D (m)
5.89
E (m)
5.13
F (dd)
4.82
J(2.62, 10.88)
G (s)
3.78
H (dd)
2.65
J(7.61, 13.93)
I (dd)
2.52
J(6.48, 13.95)
J (m)
1.96
K (ddd)
1.78
J(5.19, 10.90, 14.10)
L (ddd)
1.61
J(2.66, 8.49, 14.04)
M (dd)
1.04
J(4.17, 6.63)
Ph
O
N
COOMe
11da
1.92
1.11
2.04
0.97
2.14
1.00
3.03
1.11
1.09
1.12
1.30
1.11
6.48
f1 (ppm)

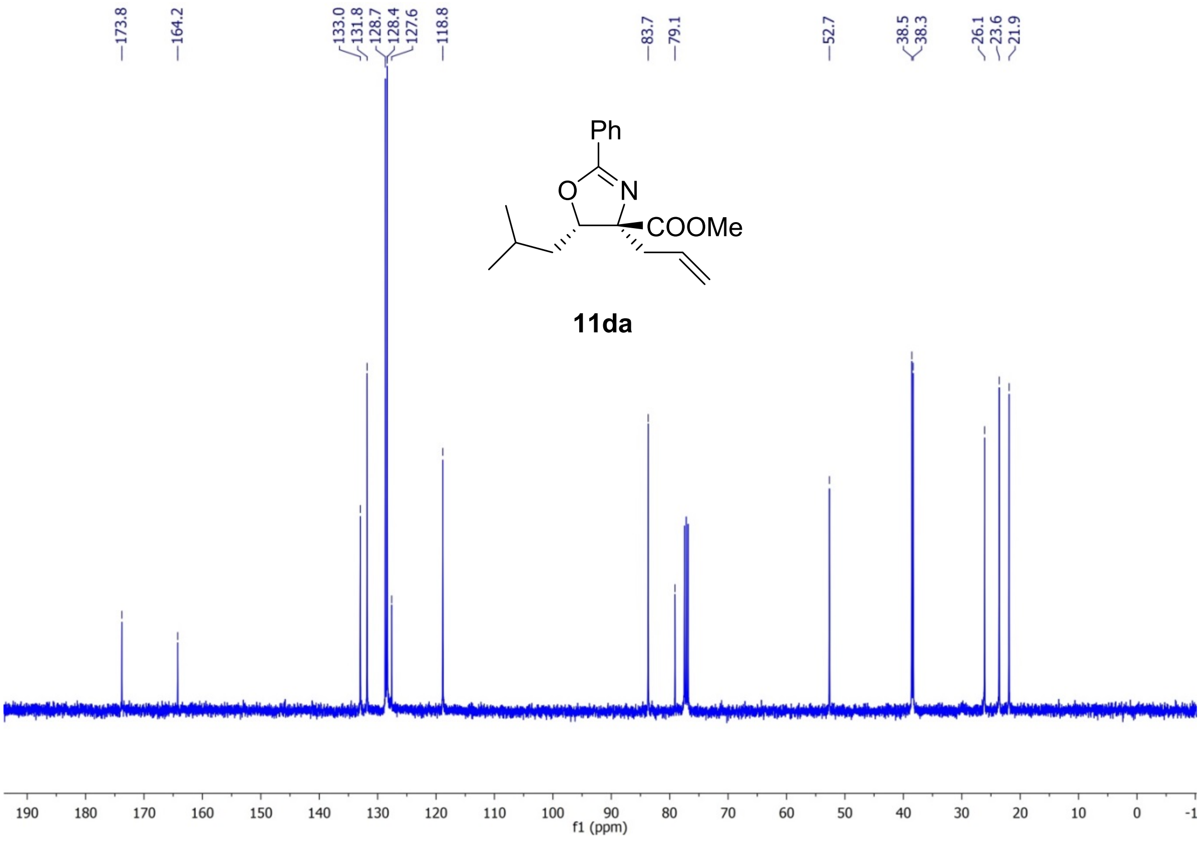
173.8
164.2
133.0
131.8
128.7
128.4
127.6
118.8
83.7
79.1
52.7
38.5
38.3
26.1
23.6
21.9
Ph
O
N
COOMe
11da
f1 (ppm)

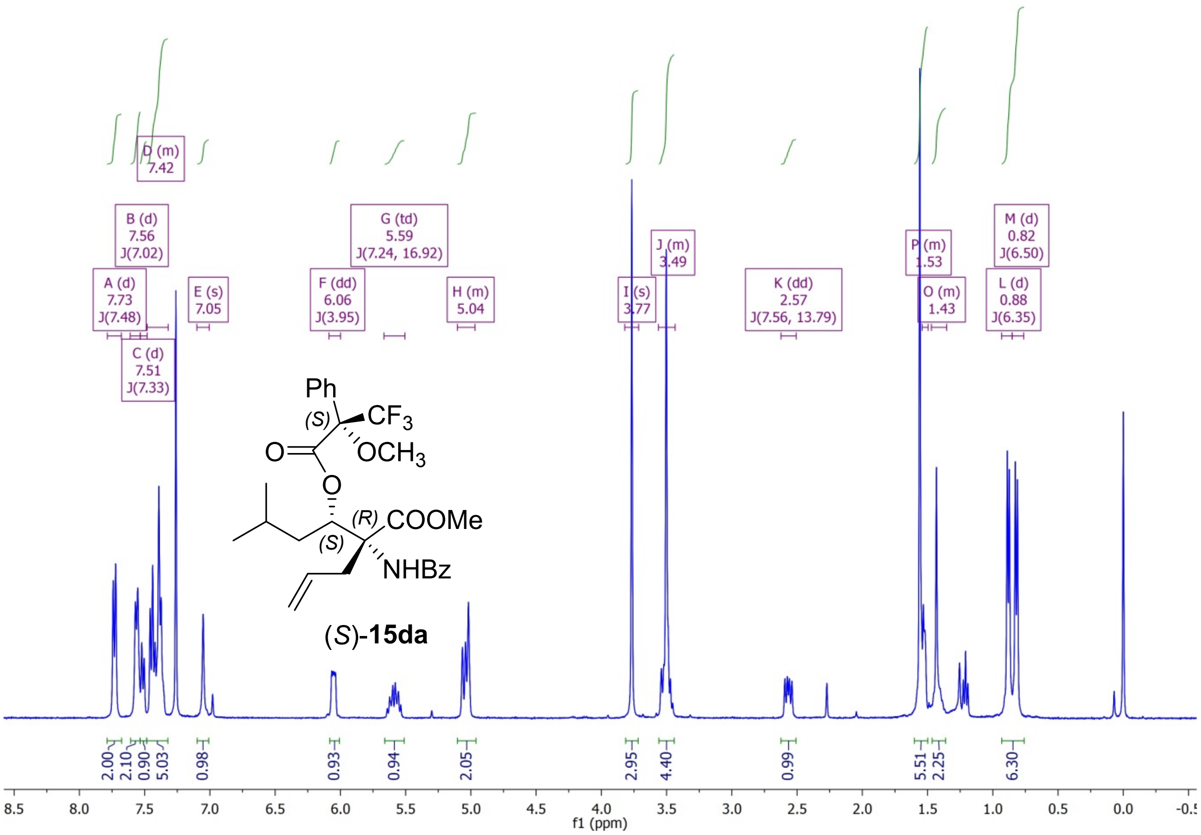
A (d)
7.73
J(7.48)
B (d)
7.56
J(7.02)
C (d)
7.51
J(7.33)
D (m)
7.42
E (s)
7.05
F (dd)
6.06
J(3.95)
G (td)
5.59
J(7.24, 16.92)
H (m)
5.04
I (s)
3.77
J (m)
3.49
K (dd)
2.57
J(7.56, 13.79)
P (m)
1.53
O (m)
1.43
M (d)
0.82
J(6.50)
L (d)
0.88
J(6.35)
Ph
(S)
CF3
O
OCH3
O
(R)
COOMe
(S)
NHBz
(S)-15da
2.00
2.10
0.90
5.03
0.98
0.93
0.94
2.05
2.95
4.40
0.99
5.51
2.25
6.30
f1 (ppm)

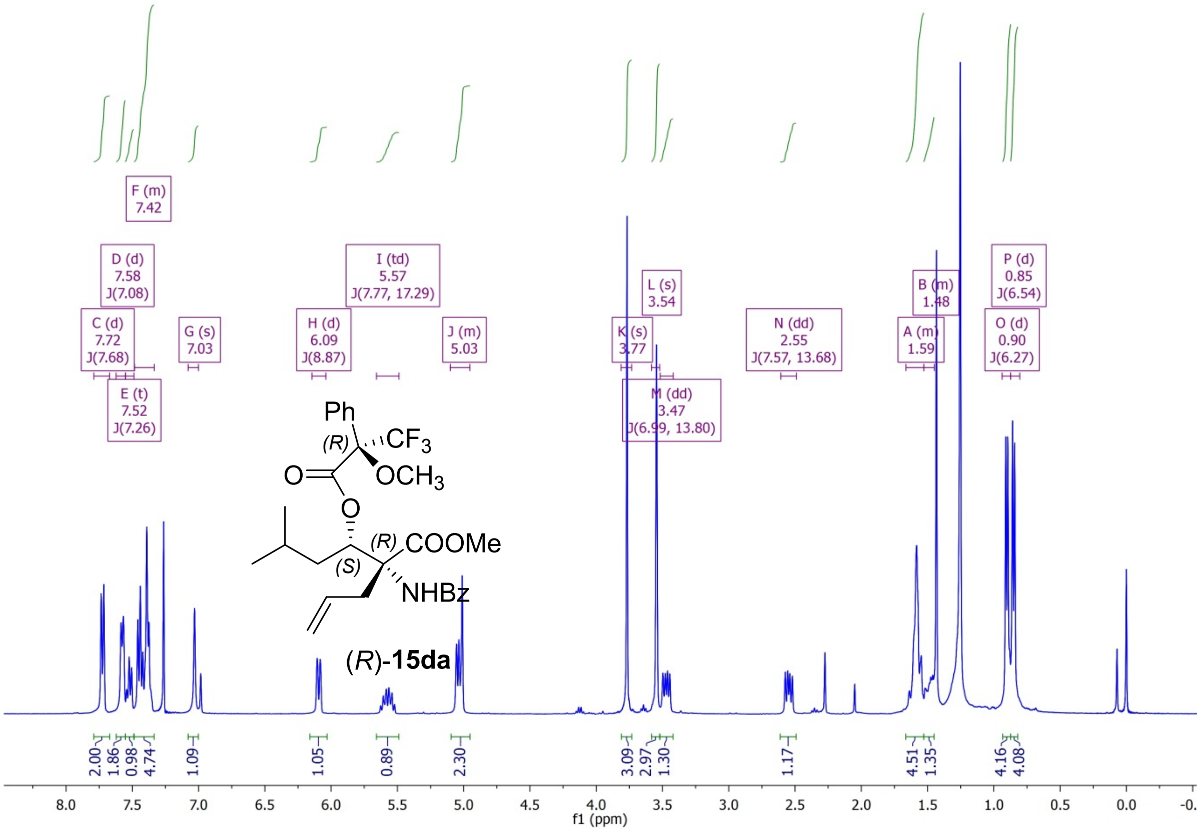
F (m)
7.42
D (d)
7.58
J(7.08)
C (d)
7.72
J(7.68)
E (t)
7.52
J(7.26)
G (s)
7.03
H (d)
6.09
J(8.87)
I (td)
5.57
J(7.77, 17.29)
J (m)
5.03
K (s)
3.77
L (s)
3.54
M (dd)
3.47
J(6.99, 13.80)
N (dd)
2.55
J(7.57, 13.68)
A (m)
1.59
B (m)
1.48
P (d)
0.85
J(6.54)
O (d)
0.90
J(6.27)
Ph
(R)
CF3
O
OCH3
O
(R)
COOMe
(S)
NHBz
(R)-15da
2.00
1.86
0.98
4.74
1.09
1.05
0.89
2.30
3.09
2.97
1.30
1.17
4.51
1.35
4.16
4.08
f1 (ppm)
